# Metabolome analysis of 20 taxonomically related benzylisoquinoline alkaloid-producing plants

**DOI:** 10.1186/s12870-015-0594-2

**Published:** 2015-09-15

**Authors:** Jillian M. Hagel, Rupasri Mandal, Beomsoo Han, Jun Han, Donald R. Dinsmore, Christoph H. Borchers, David S. Wishart, Peter J. Facchini

**Affiliations:** Department of Biological Sciences, University of Calgary, Calgary, AB T2N 1 N4 Canada; Department of Biological Sciences, University of Alberta, Edmonton, AB T6G 2E9 Canada; University of Victoria-Genome BC Proteomics Centre, University of Victoria, Victoria, BC V8Z 7X8 Canada

## Abstract

**Background:**

Recent progress toward the elucidation of benzylisoquinoline alkaloid (BIA) metabolism has focused on a small number of model plant species. Current understanding of BIA metabolism in plants such as opium poppy, which accumulates important pharmacological agents such as codeine and morphine, has relied on a combination of genomics and metabolomics to facilitate gene discovery. Metabolomics studies provide important insight into the primary biochemical networks underpinning specialized metabolism, and serve as a key resource for metabolic engineering, gene discovery, and elucidation of governing regulatory mechanisms. Beyond model plants, few broad-scope metabolomics reports are available for the vast number of plant species known to produce an estimated 2500 structurally diverse BIAs, many of which exhibit promising medicinal properties.

**Results:**

We applied a multi-platform approach incorporating four different analytical methods to examine 20 non-model, BIA-accumulating plant species. Plants representing four families in the Ranunculales were chosen based on reported BIA content, taxonomic distribution and importance in modern/traditional medicine. One-dimensional ^1^H NMR-based profiling quantified 91 metabolites and revealed significant species- and tissue-specific variation in sugar, amino acid and organic acid content. Mono- and disaccharide sugars were generally lower in roots and rhizomes compared with stems, and a variety of metabolites distinguished callus tissue from intact plant organs. Direct flow infusion tandem mass spectrometry provided a broad survey of 110 lipid derivatives including phosphatidylcholines and acylcarnitines, and high-performance liquid chromatography coupled with UV detection quantified 15 phenolic compounds including flavonoids, benzoic acid derivatives and hydroxycinnamic acids. Ultra-performance liquid chromatography coupled with high-resolution Fourier transform mass spectrometry generated extensive mass lists for all species, which were mined for metabolites putatively corresponding to BIAs. Different alkaloids profiles, including both ubiquitous and potentially rare compounds, were observed.

**Conclusions:**

Extensive metabolite profiling combining multiple analytical platforms enabled a more complete picture of overall metabolism occurring in selected plant species. This study represents the first time a metabolomics approach has been applied to most of these species, despite their importance in modern and traditional medicine. Coupled with genomics data, these metabolomics resources serve as a key resource for the investigation of BIA biosynthesis in non-model plant species.

**Electronic supplementary material:**

The online version of this article (doi:10.1186/s12870-015-0594-2) contains supplementary material, which is available to authorized users.

## Background

Metabolomics, generally defined as the measurement of all metabolites in a given system under particular conditions [45], is a key functional genomics tool with widespread applications ranging from genotype discrimination to pathology phenotyping and natural product discovery. Compared with animals, plants represent a special problem for metabolomics studies as they contain a remarkably large number (>200,000 compounds) and wide variety of metabolites [42]. Plants accumulate a plethora of specialized metabolites, many which possess potent pharmacological activities. Prominent examples include compounds of the benzylisoquinoline alkaloid (BIA) class, which occur abundantly in the order Ranunculales, particularly within Papaveraceae, Ranunculaceae, Berberidaceae and Menispermaceae families (Additional file 1). Although BIAs share a common biosynthetic origin beginning with tyrosine, branched biosynthetic pathways – many which remain unresolved at the biochemical and genetic levels – lead to the formation of diverse molecular structures (Additional file 2). Targeted study of alkaloid content has been performed for many BIA-accumulating plants, especially those with importance in modern or traditional medicinal and cultural practices [17, 51]. The emergence of increasingly sophisticated analytical platforms has supported high-resolution mass spectrometry (MS)-based BIA profiling of *Hydrastis canadensis* [29, 30], opium poppy (*Papaver somniferum*; [49]) and *Corydalis* species [27]. Hyphenated techniques such as liquid chromatography (LC)-NMR and LC-MS-NMR have been applied to alkaloid analyses of *Eschscholzia californica* [14] and *Nandina domestica* [26] cell cultures.

Only a limited number of reports have attempted to define the biochemical networks underpinning alkaloid biosynthesis. Broad-scope metabolomics encompassing both primary and secondary metabolism has been largely restricted to model plant species, including opium poppy, a longstanding model system for the study of BIA metabolism. ^1^H NMR was used to identify and quantify 34 root and 21 latex metabolites in opium poppy plants [18]. Similarly, 42 diverse metabolites were monitored in opium poppy cell cultures using NMR, revealing extensive reprogramming of primary and secondary metabolism following induction with fungal elicitor [69]. NMR-based metabolomics was reported for *Chelidonium majus*, although capacity for compound identification was limited [43]. Compound identification similarly restricted a more detailed profile of opium poppy cell cultures analyzed using FT-ICR-MS, although the occurrence of 992 distinct analytes was confirmed [70]. Technical advances made since these reports have improved the capacity for compound identification. Analyses of animal metabolomes now boast routine identification and quantification of hundreds metabolites (e.g. human urine, >400 metabolites; [6]) and progress is mirrored in plant metabolomics studies [54]. Key strategies now include the integrated use of multiple extraction procedures and analytical platforms to improve metabolic coverage and reduce bias. For example, separate extractions with water, alcohol, and organic solvents can yield different metabolite profiles [62]. Proton NMR of aqueous extracts remains a field standard in terms of the number and structural diversity of compounds identified and quantified, and a variety of biochemical databases and secondary analysis tools are now available [5, 61]. MS-based approaches complement NMR by offering enhanced spectral resolution and sensitivity for the analysis of low-abundance compounds [50] while numerous open-source and commercial software packages aid downstream analysis [53]. Choices concerning sample fractionation (i.e. type of chromatography), ion generation, and MS analyzer type [e.g. triple quadropole, time-of-flight (TOF), Orbitrap, Ion Cyclotron Resonance (ICR)] impact the nature of the resulting datasets. Both NMR and MS-based platforms are well suited for chemometric methods such as principal component analysis (PCA) and hierarchical clustering, which are often required to derive biologically relevant conclusions from complex datasets [63].

**Fig. 1:**
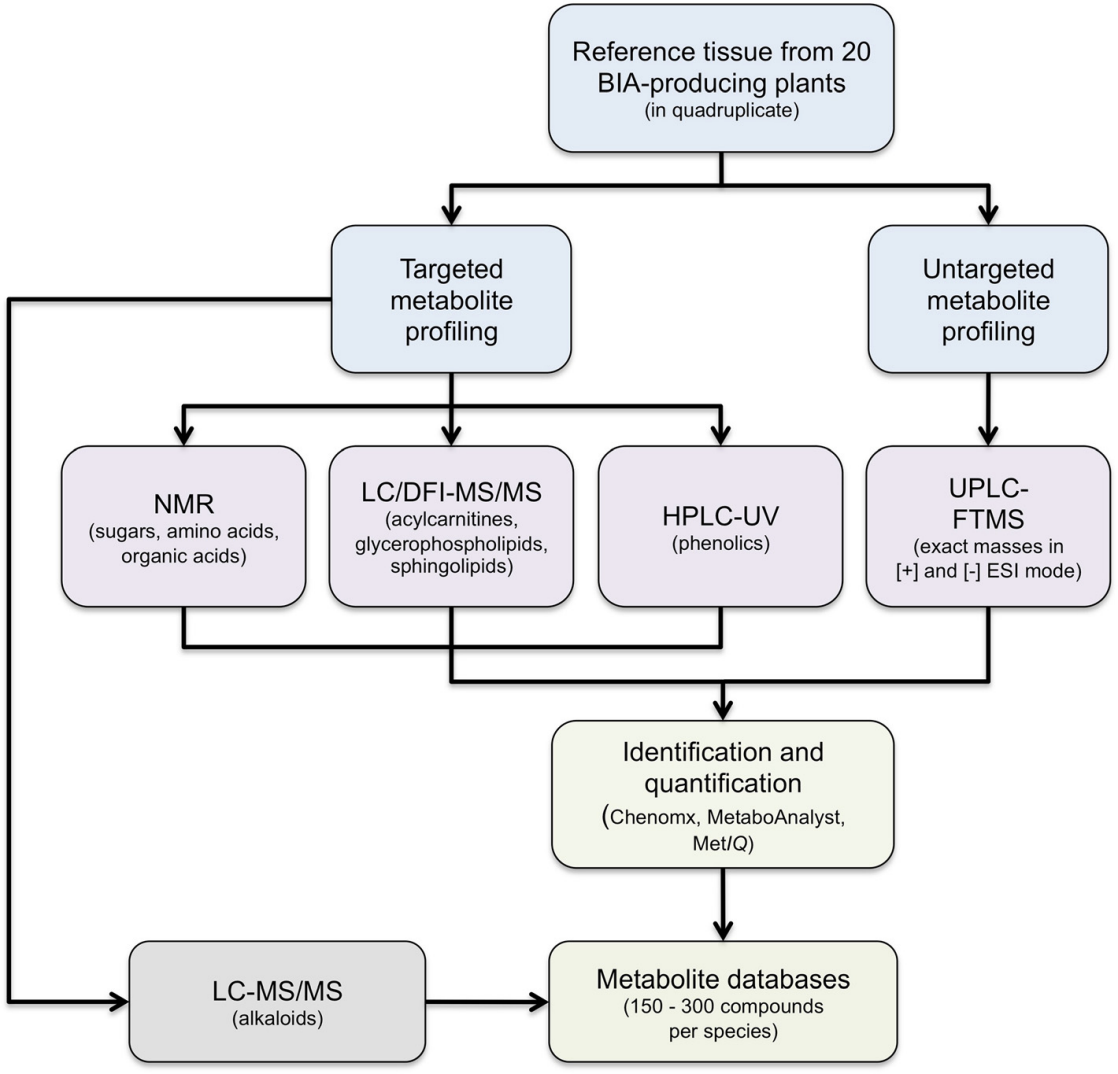
Metabolomics workflow used in this study. Five different analytical platforms (purple boxes) were employed to analyze plant tissues derived from 20 BIA-accumulating species. Metabolite classes measured using targeted methods are indicated. Identification and quantification were achieved using software packages (NMR, LC/DI-MS/MS) or manually (HPLC-UV, UPLC-FTMS). Untargeted UPLC-FTMS (+)-mode data was mined for exact masses corresponding to alkaloids. Targeted analysis using triple quadrupole LC-MS/MS was performed to acquire additional identifying information. Abbreviations: BIA, benzylisoquinoline alkaloid; NMR, nuclear magnetic resonance; LC, liquid chromatography; DFI, direct flow injection; MS, mass spectrometry; MS/MS, tandem mass spectrometry; HPLC, high-performance liquid chromatography; UV, ultraviolet light absorption spectroscopy; UPLC, ultra performance liquid chromatography; FTMS, Fourier transform mass spectrometry; ESI, electrospray ionization

We designed a multi-platform approach incorporating four different analysis methods to acquire a more complete view of the biochemical networks operating in non-model, BIA-accumulating plants (Fig. 1). This study represents the first time a metabolomics approach has been applied to most of these species, despite their importance in modern and traditional medicine [17]. The large data collections reported herein serve as a key resource for (i) research of the biochemical mechanisms governing alkaloid metabolism, (ii) novel gene discovery, and (iii) future metabolic engineering efforts. Metabolite and transcript resource development has greatly expedited novel gene discovery in model systems such as opium poppy, permitting the near-complete elucidation of several major BIA pathways. In tandem with our accompanying transcriptome analysis [19], the goal of this work was to establish equivalent resources for plants displaying distinct and unexplored BIA profiles.

## Results and discussion

### Species and tissue selection for alkaloid structural diversity and enrichment

Twenty plant species were chosen for metabolomics analysis based primarily on reported alkaloid accumulation profiles, as determined by relevant literature. Other factors included taxonomic distribution, tissue availability, and uses in traditional medicine or cultural practices (signaling potential presence of pharmacologically active BIAs).

Focus was placed on Ranunculales members belonging to one of four families: Papaveraceae (8 species), Ranunculaceae (4 species), Berberidaceae (4 species), and Menispermaceae (4 species) (Table 1). Although BIAs have been reported in diverse angiosperm taxa, they occur most commonly in these four families [17]. Many different BIA structural subtypes occur in the Ranunculales, including twelve shown in Additional file 2. In an effort to maximize diversity in terms of alkaloid types, we selected plants reportedly rich in a wide variety of different BIAs. For example, *Corydalis* and *Papaver* species are known to accumulate a plethora of different BIAs, including morphinan, protoberberine, benzophenanthridine, aporphine, pavine and phthalideisoquinoline alkaloids [24, 25]. *Menispermum canadense* produces highly unusual acutumine alkaloids, in addition to bisbenzylisoquinoline alkaloids more typical of Menispermaceae family members [10]. Diversity was also captured within Berberidaceae and Ranunculaceae families; *Thalictrum* species, for example, produce oxobenzylisoquinoline alkaloids, secoisoquinoline derivatives, and differentially substituted aporphine molecules [2, 46, 56]. Roots, stems or rhizomes were selected for metabolomics profiling based on literature sources indicating accumulation sites of BIAs. This was a key consideration as BIA content can vary considerably in different organs. For example, sanguinarine in *Sanguinaria canadensis* occurs at concentrations ten- and one thousand-fold lower in roots and aerial organs (leaves, flowers), respectively, compared with the accumulation in rhizomes [8]. Plant-specific circumstances (e.g. uses in medicine, conservation status) were secondary considerations when selecting species and organ type. For instance, *Hydrastis canadensis* is important for traditional medicine among certain First Nations of Canada, and owing to modern overharvesting practices is now regarded as a threatened species [3]. These circumstances, together with a distinct, phthlideisoquinoline-rich BIA profile [29] led to the selection of *H. canadensis* a target plant. In three species, callus culture was used in place of differentiated organs (Table 1). As an alternative to intact plants, cell cultures have been used for more than three decades as biosynthetic models and alkaloid production systems [67]. The inclusion of callus in this study permitted general comparisons between metabolomes of intact plants versus cell cultures.

**Table 1:** Details of plant species selected for metabolomics analysis

#	Species	Abbrev.	Common name	Family (Tribe)	Organ/tissue
1	*Argenome mexicana*	AME	Mexican Prickly Poppy	Papaveraceae (Papaveroideae)	Stem
2	*Chelidonium majus*	CMA	Greater Celandine	Papaveraceae (Papaveroideae)	Stem
3	*Papaver bracteatum*	PBR	Persian Poppy	Papaveraceae (Papaveroideae)	Stem
4	*Stylophorum diphyllum*	SDI	Celandine Poppy	Papaveraceae (Papaveroideae)	Stem
5	*Sanguinaria canadensis*	SCA	Bloodroot	Papaveraceae (Papaveroideae)	Rhizome
6	*Eschscholzia californica*	ECA	California Poppy	Papaveraceae (Papaveroideae)	Root
7	*Glaucium flavum*	GFL	Yellow Horn Poppy	Papaveraceae (Papaveroideae)	Root
8	*Corydalis chelanthifolia*	CCH	Ferny Fumewort	Papaveraceae (Fumarioideae)	Root
9	*Hydrastis canadensis*	HCA	Goldenseal	Ranunculaceae	Rhizome
10	*Nigella sativa*	NSA	Black Cumin	Ranunculaceae	Root
11	*Thalictrum flavum*	TFL	Meadow Rue	Ranunculaceae	Root
12	*Xanthorhiza simplicissima*	XSI	Yellowroot	Ranunculaceae	Root
13	*Mahonia aquifolium*	MAQ	Oregon Grape	Berberidaceae	Bark
14	*Berberis thunbergii*	BTH	Japanese Barberry	Berberidaceae	Root
15	*Jeffersonia diphylla*	JDI	Rheumatism Root	Berberidaceae	Root
16	*Nandina domestica*	NDO	Sacred Bamboo	Berberidaceae	Root
17	*Menispermum canadense*	MCA	Canadian Moonseed	Menispermaceae	Rhizome
18	*Cocculus trilobus*	CTR	Korean Moonseed	Menispermaceae	Callus
19	*Tinospora cordifolia*	TCO	Heartleaf Moonseed	Menispermaceae	Callus
20	*Cissampelos mucronata*	CMU	Abuta	Menispermaceae	Callus

### ^1^H NMR profiling highlights species- and tissue-specific variation in sugar, amino acid and organic acid content

**Fig. 2:**
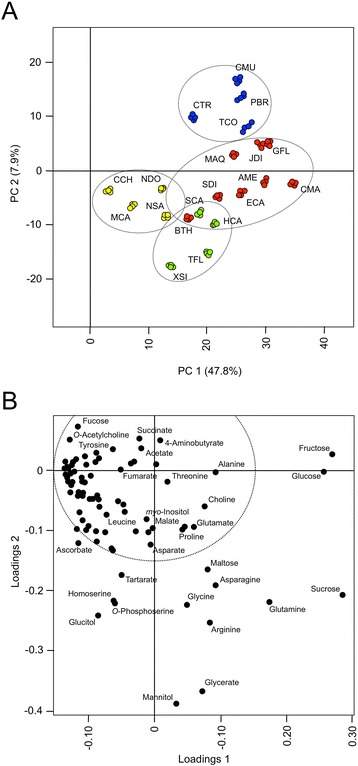
Two-dimensional principal component analysis (PCA) of metabolite quantities obtained using NMR-based profiling. Results are presented as scores (**a**) and loadings (**b**) plots. The percent variance accounted for by each principal component (PC) is indicated. For the scores plot, each dot represents a one of four replicates analyzed per plant species. Areas enclosed by 95 % confidence ellipses, containing dots of the same color, define statistically significant class separations [34]. Species abbreviations are defined in Table 1. Loadings representing individual metabolites are shown as black dots (**b**). Metabolite names are indicated for loadings contributing more to variance. A complete listing of loadings data is found in Additional file 16

**Fig. 3:**
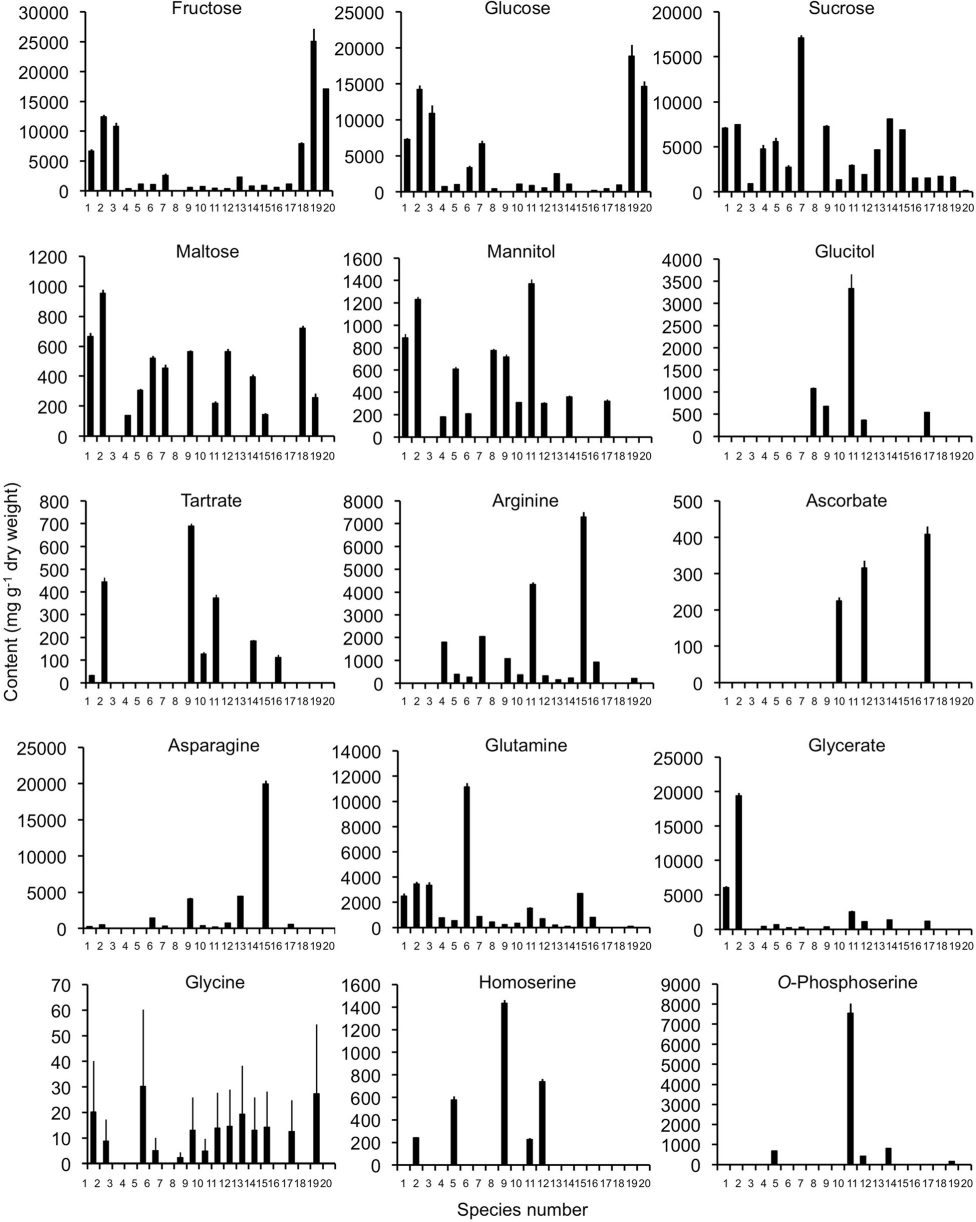
Individual metabolite quantities as determined by NMR analysis for 20 BIA-accumulating plant species. Plant species are designated by number, as defined in Table 1. Averages ± SD were calculated using 4 replicates per species

Metabolite surveying by NMR was expected to favor sugars, amino acids and organic acids, as these constitute the most abundant, water-soluble primary metabolites found in plants [62]. Most benzylisoquinoline alkaloids are sparingly soluble in pure water and extract more efficiently with alcohol or water-alcohol mixtures [18, 40]. To place focus on primary metabolites, plant extractions for NMR were performed using water, whereas methanol extraction enriched alkaloid content prior to UPLC-FTMS. Chenomx NMR Suite 7.0 was used for NMR spectral analysis owing to its capacity for targeted identification and quantification of >300 metabolites in complex samples (http://www.chenomx.com). Standard spectra for benzylisoquinoline alkaloids are not yet represented in Chenomx libraries; this reality, in addition to poor solubility in pure water, precluded alkaloid structural analysis. Nonetheless, a total of 91 metabolites were identified and quantified using one-dimensional (1D) ^1^H NMR and Chenomx NMR Suite support. Additional file 3 lists the compounds detected and their abundances in each of 4 replicates performed for every plant species. Metabolite quantities formed the basis for principal component analysis (PCA) using MetaboAnalyst v. 2.0 [65]. PCA is an unsupervised method used to summarize large datasets as more easily interpreted principal component (PC) scores. The goal of PCA is to account for as much variance in the data as possible using the smallest number of PCs [11, 23]. Typically, 2- or 3-dimensional scores plots are sufficient for meaningful interpretation of metabolomics data. Accompanying loadings plots are used to interpret the patterns of scores plots. In particular, loadings plots can reveal the metabolites (termed ‘loadings’ in this context) that contribute most to the variance between samples. Figure 2a and b illustrate PCA scores and loadings plots, respectively, for NMR-based metabolite quantities. The most abundant metabolites detected by NMR were sugars, sugar acids and alcohols, and various amino acids including glutamine, asparagine and arginine (Additional file 3). However, metabolite quantities varied dramatically between species, forming a basis for overall variance. For instance, loadings corresponding to fructose, glucose and sucrose are important contributors to variance along PC1 (Fig. 2b), which distinguished sugar-rich *Chelidonium majus* (CMA), *Argemone mexicana* (AME), and *Glaucium flavum* (GFL) from sugar-depleted *Corydalis chelanthifolia* (CCH) tissues (Fig. 2a). Individual plots for these and other metabolites contributing to variance are shown in Fig. 3. Whereas CMA roots were depleted of saccharides, a number of other metabolites such as glucarate and arabinitol were found exclusively in these tissues. Polyols such as arabinitol are mainly associated with the fungal kingdom [33] and can be elevated in root ectomycorrhizae relative to free-living fungi [35]. Saccharide depletion and arabinitol enrichment in CCH roots could reflect the presence of symbiotic fungi. An evaluation of associated transcriptomics data for CCH roots [19] revealed the presence of non-plant sequences with high homology to DNA of uncultured rhizosphere isolates and other unidentified fungi, bacteria and protists. While it is clear that various microorganisms were associated with CCH root at the time of RNA extraction, BLAST results were insufficient to ascertain the nature of any plant-microbe interactions, if such relationships were present.

Callus cultures were distinguished from most other plant tissues, particularly along PC2 (Fig. 2a). This result was partially explained by low abundances of certain amino acids (e.g. glutamine, arginine), glycerate and mannitol (Fig. 2b, Fig. 3, Additional file 3). Similar results were found for *Papaver bracteatum* (PBR) stem tissues, highlighted by clustering of PBR with *Cocculus trilobus* (CTR), *Cissampelos mucronata* (CMU) and *Tinospora cordifolia* (TCO) data (Fig. 2a). With some exceptions, mono- and disaccharide sugars were generally lower in roots and rhizomes compared with stems, perhaps reflecting the presence of lignified secondary xylem in these tissues.

### DFI-MS/MS reveals ubiquitous presence of key membrane lipids and acylcarnitines

To detect less polar metabolites including long-chain acylcarnitines, glycerophospholipids and sphingolipids, extraction was performed using organic solvents in preparation for direct flow injection (DFI)-MS/MS analysis. A kit-based, targeted, quantitative approach using Direct Flow Injection (DFI) with tandem mass spectrometry (MS/MS) was used for detection and quantification of carnitines, phospho- and sphingolipids. In total, 110 metabolites were detected and quantified in the 20 BIA-accumulating plant species. Detected compounds belonged to one of three classes of lipids distinguished by the *O*- or *N*-linked head group: acylcarnitines, glycerolipids and sphingolipids. Compound identities and abundances are listed in Additional file 4. Phosphatidylcholine (PC) molecules exhibiting partially desaturated, *O*-linked diacyl (aa) chains of variable length totaling 34 or 36 carbons (sum of both acyl chains) were by far the most abundant metabolites. In plants and animals, PC lipids represent major components of plasma and mitochondrial membranes and endoplasmic reticulum [57]. C34 or C36 diacyl (aa) lipids with one or more double bonds (e.g. PC aa C34:1, PC aa C34:2, etc.) ranged up to 1000-fold more abundant than equivalent PC lipids exhibiting shorter chain lengths. These results reflect PC content of *Arabidopsis* [39] and were not surprising, as fatty acids 16 or 18 carbons in length generally constitute the bulk of fatty acids synthesized in the chloroplast stroma prior to further derivitization. Diacyl PC lipids were more abundant than mixed-chain (i.e. alkyl-ester or ‘ae’) and single-chain (i.e. ‘lyso’) lipids. For example, the average level of PC aa C34:2 was 37-fold greater than PC ae C34:2. Phosphatidylglycerol (PG) lipids, which are specific to thylakoid membrane, and glycolipids, which predominate the chloroplast envelope and thylakoid membrane, were not measured using the AbsoluteIDQ p150 kit. Despite the large compound library accessible using this kit, areas of application are generally animal-focused and certain plant-specific metabolites are not yet represented (http://www.biocrates.com/products/research-products/absoluteidq-p150-kit). Twenty-eight acylcarnitine metabolites were measured, in addition to 5 sphingolipids. Compared with animals, plant acylcarnitines are present at low levels and little is known regarding their biological role, aside from perceived involvement in fatty acid metabolism [7, 44]. PCA indicated that PC levels were important contributors to variance along PC1, which distinguished roots of *Berberis thunbergii* (BTH), *Xanthorhiza simplicissima* (XSI) and *Corydalis chelanthifolia* (CCH) from tissues exhibiting higher levels of several PC classes (Additional file 5). Additionally, the combined contribution of PCs and acylcarnitines to variance along PC2 appeared to distinguish *Eschscholzia californica* (ECA) from other species. Further investigation regarding the biological role of plant acylcarnitines will help explain these observations.

### Phenolic content suggests impact of environmental factors

A HPLC-UV-based method targeting plant phenolics was used to identify and quantify 15 metabolites including flavonoids, benzoic acid derivatives and hydroxycinnamic acids (Additional file 6). With the exception of the two benzoic acid derivatives (i.e. syringic and gallic acid) all phenolic compounds examined in this study derive from the phenylpropanoid pathway. Phenylpropanoids comprise nearly 20 % of total carbon in the terrestrial biosphere [66] and more than 7000 different phenylpropanoid compounds are found in plants [60], where they function as pigments, cell wall components, scent compounds and signaling molecules. Despite the abundance of these compounds and their ubiquitous presence in plants, occurrence and quantity of phenolic compounds can vary dramatically from species to species, and also within single species depending on environmental, developmental, or genetic factors. For example, substantial quantitative differences in various flavonoids were reported for seeds of different *Arabidopsis thaliana* accessions, indicating that even minor changes in genetic background can influence polyphenol content [47]. Both quantitative and qualitative differences in phenolic content were observed in the present study, and a clear distinction was made between cultured tissue and intact plants. PCA defined callus of CMU, TCO and CTR from roots and stems of other species along PC1, which accounted for >40 % of the observed variance in the data (Additional file 7A). Callus cultures were low or lacking in a number of phenolic compounds, including luteolin and kaemferol, which were comparatively abundant in root and stem tissue (Additional file 8) and contributed to variance along PC1 (Additional file 7B). Luteolin and kaemferol are flavonoids with photoprotective function and are known to accumulate in response to sun irradiance and UV stress [1, 20]. Further, flavonoids are enriched in root cortical tissues, likely providing protection against biotic and abiotic stresses [38]. Callus, which was grown under sterile conditions in the dark, could be lower in these compounds owing to an absence of elicitation by light or other environmental stresses.

The abundance of several compounds varied widely between species. For example, quercetin levels were >35 mg g^−1^ dry weight in *Stylophorum diphyllum* stem but barely detectable (<1 mg g^−1^) in several other species. Although phenolic accumulation profiles are influenced by genetic factors, it is possible that the metabolite content of these plants, which were cultivated in a variety of different locations, both outdoors and in greenhouses, was directly impacted by environmental factors. Coumarin, which was generally low in stem tissues, was abundant in the roots of several species, particularly *Corydalis chelanthifolia* (CCH) (Additional file 8). CCH roots were also abundant in cinnamic and syringic acids compared with other species. Fungal challenge is known to cause cinnamic and syringic acids secretion by roots [28]. It is possible that CCH roots, and possibly others examined in this study, were exposed to microbial challenge prior to analysis.

### UPLC-FTMS analysis distinguishes callus from BIA-rich plant tissues

Untargeted UPLC-FTMS profiling was expected to generate extensive mass lists corresponding to a wide variety of metabolites, including alkaloids. Analysis performed in positive ion mode detected an average of 412 compounds, listing up to >1200 distinct masses (e.g. for *Eschscholzia californica*), each assigned their own chromatographic retention time (R_t_) (Additional file 9). In contrast, analysis performed in negative ion mode yielded an average of 799 compounds per species, with a maximum of 1767 masses for *E. californica* (Additional file 10). Although the large number of metabolites detectable by MS-based approaches is important for untargeted applications such as biomarker discovery and pathology diagnostics, compound identification remains a challenge [22]. Nonetheless, MS-based methods are important to the analysis of alkaloids, which are often present in low abundance. To gain insight into the BIA content of the 20 selected plant species, we mined the positive ion mode datasets (Additional file 9) and identified exact masses corresponding to empirical formulae of known alkaloids. These masses and associated information (predicted atomic compositions, R_t_ and putative BIA assignments) were compiled in a separate file (Additional file 11) representing a condensed, alkaloid-specific listing. Since structural isomerism is common among BIAs, many compounds share the same empirical formula and therefore share identical masses. In such cases of ambiguity, masses were assigned not to a single BIA, but groups of alkaloids sharing the same empirical formula (i.e. ‘mass groups’). The list of known BIAs (organized by mass group) used to mine the positive mode dataset is found in Additional file 12.

**Fig. 4:**
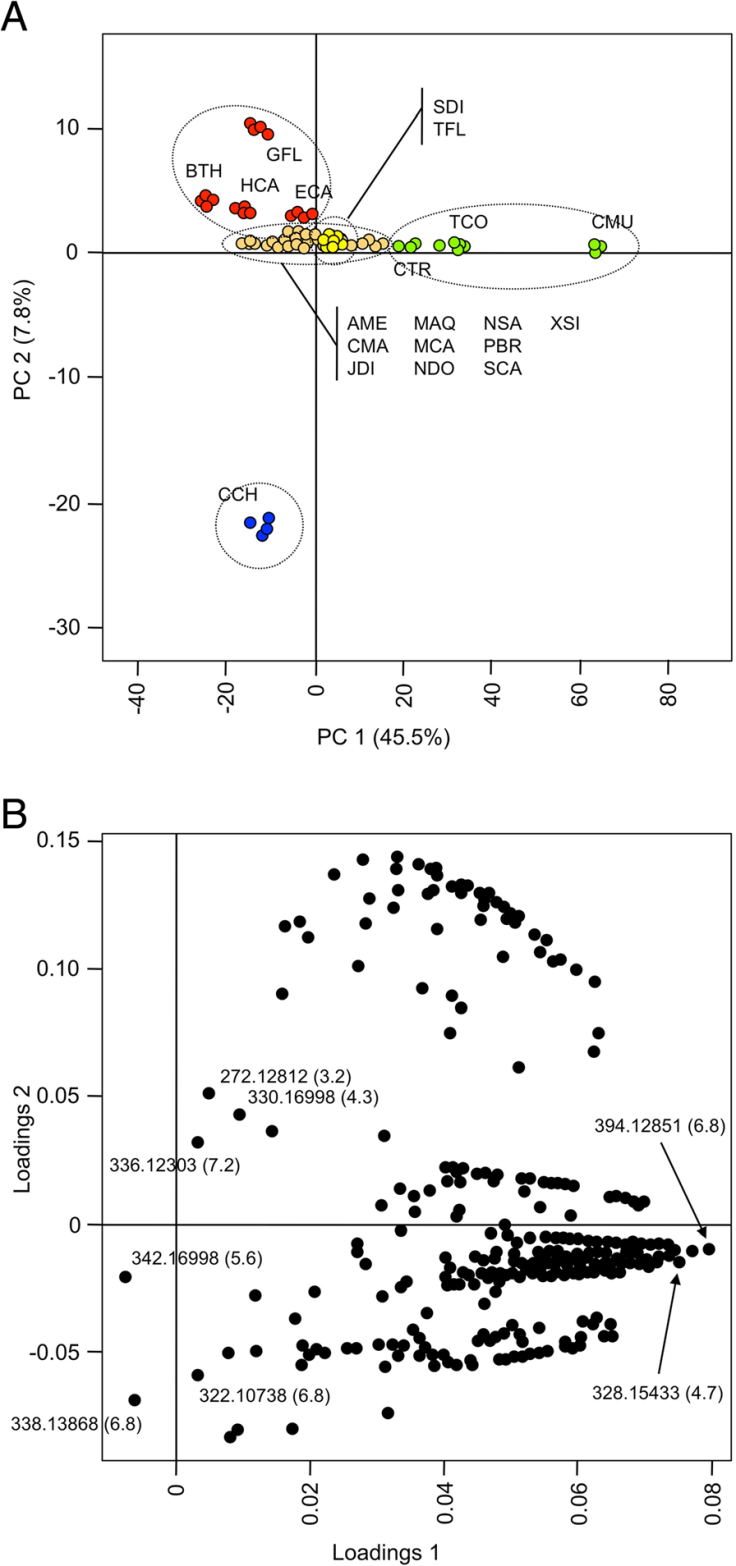
Two-dimensional principal component analysis (PCA) of relative abundances (ion counts) of ion masses (*m*/*z*) detected using UPLC-FTMS. Only masses corresponding to those expected for alkaloids, extracted from positive mode data, are included. Results are presented as scores (**a**) and loadings (**b**) plots. The percent variance accounted for by each principal component (PC) is indicated. For the scores plot, each dot represents a one of four replicates analyzed per plant species. Areas enclosed by 95 % confidence ellipses, containing dots of the same color, define statistically significant class separations [34]. Species abbreviations are defined in Table 1. Loadings representing individual masses are shown as black dots (**b**). Masses and putative identities, where applicable, are shown for select loadings. A complete listing of loadings data is found in Additional file 16

**Fig. 5:**
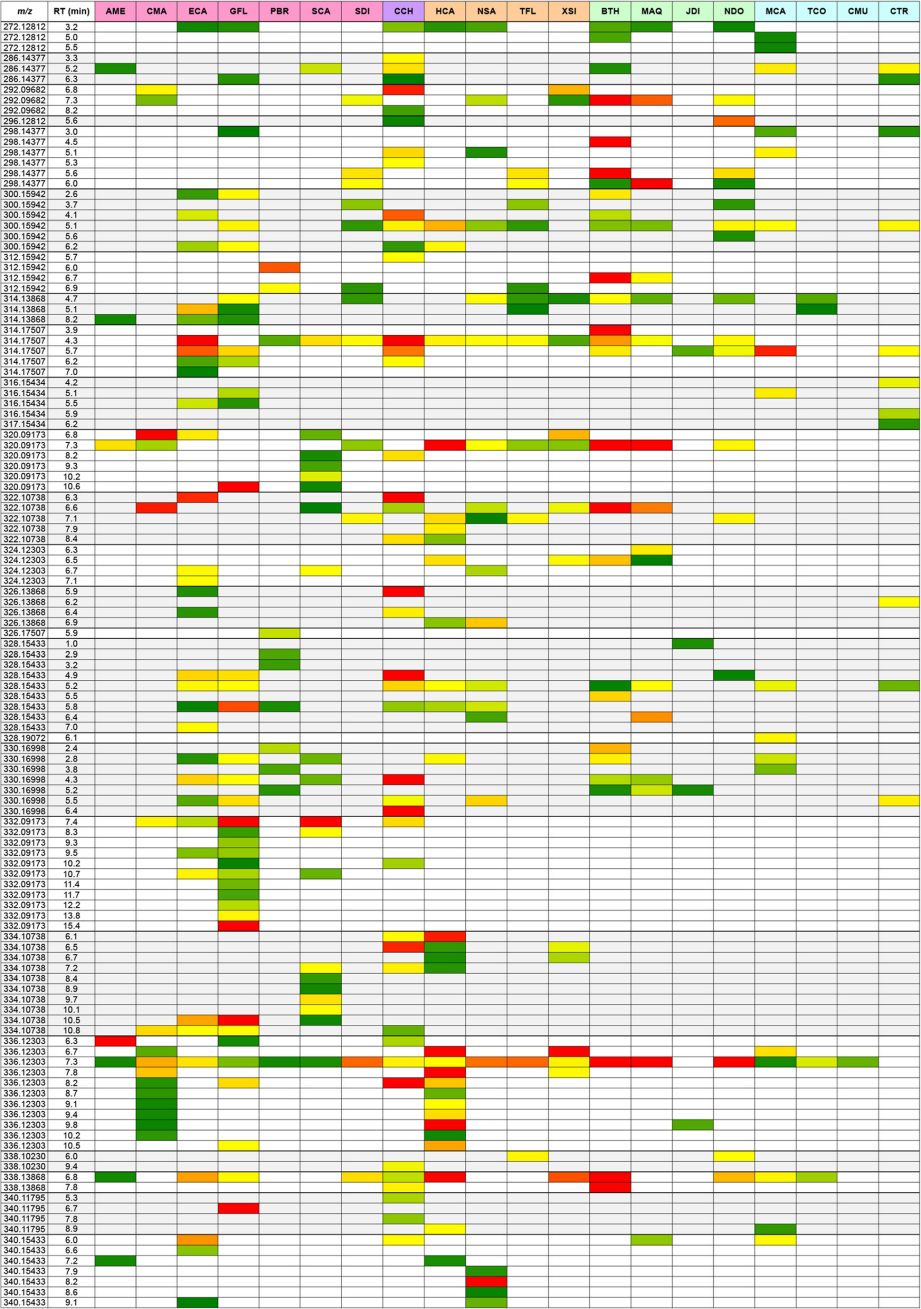
Relative ion abundances in 20 plant species. Only masses corresponding to those expected for alkaloids, extracted from positive mode FTMS data, are shown. UPLC retention times (R_t_) for each ion mass (*m*/*z*) are provided to distinguish identical masses, which presumably represent different structural isomers. Variability in ionization efficiencies should be considered when comparing abundances between different ions. Relative abundance scale (green-yellow-red) highlights quantitative differences for each mass across different species. Species abbreviations are defined in Table 1. Species are grouped according to family or tribe: (pink = Papaveroideae; purple = Fumarioideae; orange = Ranunculaceae; green = Berberidaceae; blue = Menispermaceae)

**Fig. 6:**
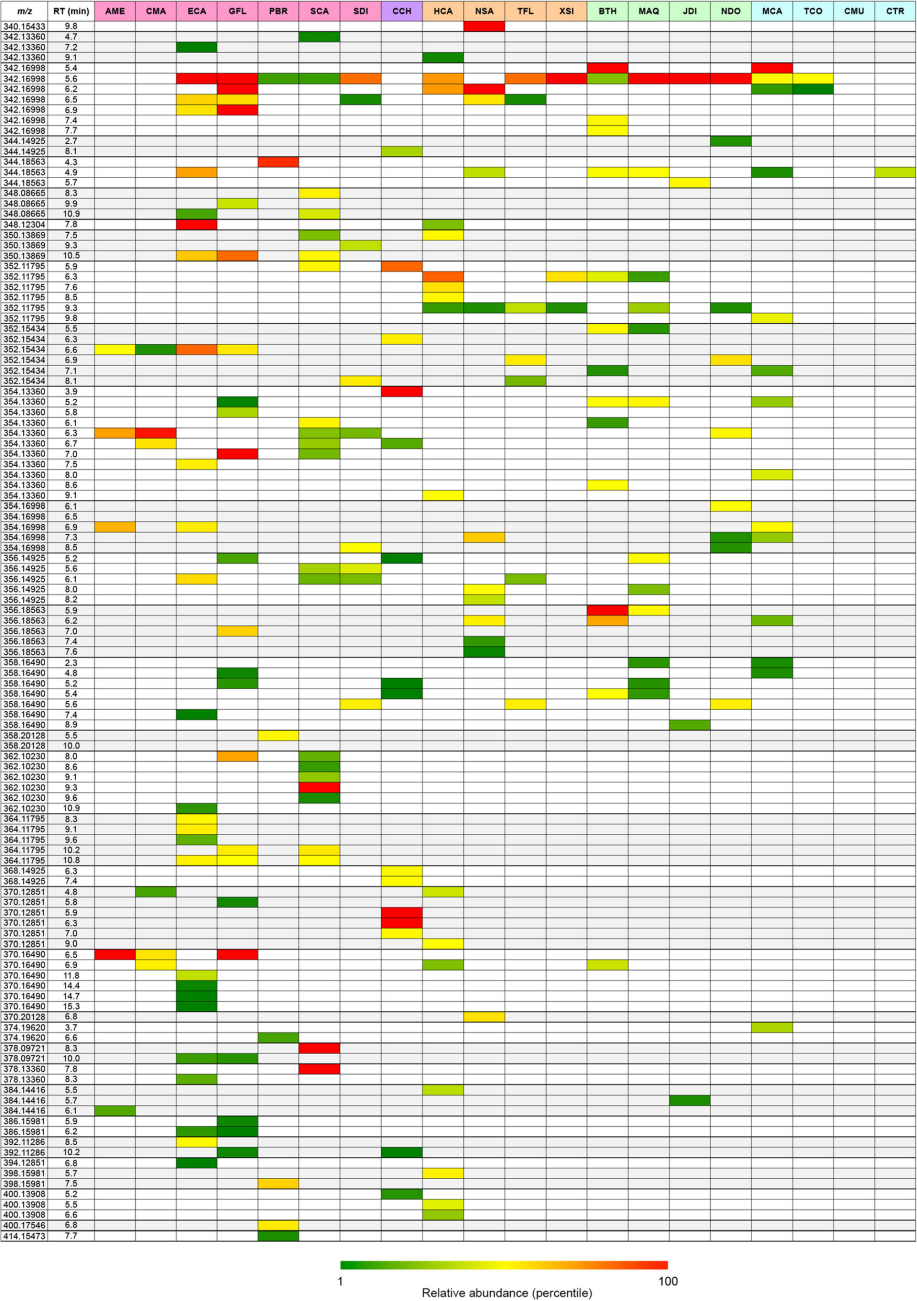
Relative ion abundances between *m/z* 340.15433 and *m/z* 414.15473 in 20 plant species. Masses extracted from positive mode FTMS data and corresponding to those expected for alkaloids are shown. UPLC retention times (R_t_) for each ion mass (*m/z*) are provided to distinguish identical masses, which presumably represent different structural isomers. Variability in ionization efficiencies should be considered when comparing abundances between different ions. The relative abundance scale (green-yellow-red) highlights quantitative differences for each mass across different species. Species abbreviations are defined in Table 1. Species are grouped according to family or tribe: (pink = Papaveroideae; purple = Fumarioideae; orange = Ranunculaceae; green = Berberidaceae; blue = Menispermaceae).

Standards were not included as part of the UPLC-FTMS profiling, disallowing absolute quantification. However, comparison of ion counts provided a snapshot of relative BIA abundances. Ion counts formed the basis for PCA as shown in Fig. 4a (scores plot) and 4B (loadings plot). Although variability in ionization efficiencies between structurally different BIAs likely impacted observed abundances to some extent and caution should be exercised when making head-to-head comparisons, general conclusions can still be drawn from multivariate analysis. These results are presented as heat maps in Fig. 5 and 6, enabling comparison of relative alkaloid abundances across species. PC1, which accounted for more than 45 % of the variance in the dataset, clearly distinguished callus tissue (CMU, TCO, CTR) from root, rhizome and stem samples (Fig. 4a). Visual inspection of revealed a comparative lack of masses corresponding to BIAs in callus tissue, especially in CMU (Fig. 5 and 6). Only a single mass (*m*/*z* 336.12303) eluting at R_t_ = 7.3 min was detected in CMU, compared to an average of 32 putative alkaloids per species for differentiated tissues. Situations where alkaloid is abundant in whole plants but absent in cultured cells occur frequently, although alkaloid biosynthetic machinery (e.g. mRNA, enzymes) is often still present [68]. For example, opium poppy cell cultures are devoid of morphinan alkaloids, despite the plethora of these compounds in whole plants. Yet, resources derived from opium poppy cell cultures have lead to the discovery of several enzymes participating in morphine biosynthesis [17].

### Putative identification of BIAs reveals leads for gene discovery

Masses occurring most frequently across different species include *m*/*z* 342.16988 (R_t_ = 5.6 min), 314.17507 (R_t_ = 4.3 min) and 336.12303 (R_t_ = 7.3 min). The last of these masses likely corresponded to berberine, a common alkaloid present throughout the Ranunculales [12]. As standards were not available during UPLC-FTMS analysis, we performed an additional triple quadrupole LC-MS/MS study on identical tissues using 23 available alkaloid standards, which allowed definitive identification of several unknowns. The presence of berberine was confirmed in several species and compound identity was ascertained through collision-induced dissociation (CID) analysis. LC-MS/MS results are summarized as representative, annotated chromatographs (Additional file 13) and CID peak lists (Additional file 14). Although many BIAs share the formula C_20_H_24_NO_4_ (*m*/*z* 342.16988) (Additional file 12), a metabolite with this composition running at 5.6 min occurred frequently (14/20 species) and could be magnoflorine (Fig. 6). Like berberine, magnoflorine is a common BIA found across all families of the Ranunculales and occurring sporadically in unrelated orders [21, 32, 37, 40]. PCA suggested that loadings putatively corresponding to berberine and magnoflorine (Fig. 4b) contributed to the distinction observed between alkaloid-rich and alkaloid-depleted plant tissues.

*Corydalis chelanthifolia* (CCH) was rich in several alkaloids found in few or no other species, a result likely contributing to a clear separation of CCH from other plants along PC2 (Fig. 4a). Also, certain masses abundant in other plants (e.g. *m*/*z* 342.16998, R_t_ = 5.6 min) were absent in CCH. Masses unique to CCH included those potentially corresponding to phthalideisoquinoline alkaloids. For example, *m*/*z* 370.12851 (R_t_ = 5.9 and 6.3 min) could potentially represent corledine, corlumidine or severtzine, all with the empirical formula C_20_H_20_NO_6_ and so far found exclusively in *Corydalis* species [4]. Other compounds with this formula include the phthalideisoquinoline egenine and the secoberbine (*R*)-canadaline, which are largely restricted to the Fumarioideae tribe (Table 1) [16, 36, 64]. (*S*)-Canadaline, a potential precursor to the phthalideisoquinoline hydrastine (*m*/*z* 384.14416) [36] is known to occur in *Hydrastis canadensis*. Masses corresponding to these alkaloids were found in HCA rhizome (Fig. 6). Unlike phthlideisoquinoline alkaloids of the model plant opium poppy (e.g. noscapine), hydrastine, corledine, corlumidine, severtzine and egenine all lack a 4′-hydroxyl or 4′-methoxyl group (Additional file 15). The presence of this group is an absolute requirement for noscapine biosynthesis in opium poppy, as a key CYP82 enzyme (1-hydroxy-*N*-methylcanadine 13-hydroxylase) will not accept substrates lacking a hydroxyl function at this position [9]. The presence of non-hydroxylated phthalideisoquinoline alkaloids in CCH and HCA could signify a CYP82 variant with a different substrate acceptance profile or an alternative biosynthesis. Eleven and eight CYP82 homologues are found in HCA and CCH transcriptomes, respectively [19]. Testing these enzymes for involvement in the biosynthesis of non-hydroxylated phthalideisoquinoline alkaloids could help elucidate pathways in HCA and CCH.

Interestingly, FTMS results revealed that PBR could be producing phthalideisoquinoline alkaloids, albeit in low amounts. A single mass putatively representing noscapine (*m*/*z* 414.15473) was found in PBR stem (Fig. 6). These results are important to the process of gene discovery, especially when considered together with transcriptomics data. For example, several uncharacterized genes expressed in PBR stem have significant homology to those with established involvement in noscapine biosynthesis in opium poppy [19]. Identification of a mass corresponding to noscapine in PBR adds weight to the hypothesis that these PBR genes are functional homologues with roles in phthlideisoquinoline biosynthesis. Further, the appearance of unique masses in species such as CCH enables ‘hypothesis-driven mining’ whereby suspected occurrence of target alkaloids enables rational candidate gene selection and assay design. For instance, the presence of masses possibly corresponding to phthlideisoquinoline alkaloids such as egenine in CCH could form the basis for testing uncharacterized, noscapine-synthase (NOS)-like genes from CCH, using phthlideisoquinoline substrates such as bicuculline, a suspected product of egenine oxidation [16]. An analogous reaction in opium poppy involves NOS-catalyzed oxidation of narcotine hemiacetal to noscapine [9]. Six CCH genes with substantial homology to opium poppy NOS were identified in CCH transcriptome [19]. Essentially, this metabolite list (Fig. 5 and 6) can be used as a guide when choosing novel genes to test for hypothesized activity.

## Conclusions

An important goal of metabolomics is to acquire information regarding as many metabolites as possible, which requires the use of more than one analytical platform. The multi-faceted approach taken in this study combined five different techniques in order to gain a broader and more accurate snapshot of primary and secondary metabolism within 20 different BIA-accumulating plant species. Differences in the profile of primary metabolites were observed between different source tissues (e.g. callus versus differentiated organs, stem versus root/rhizome) that could relate to variation observed in alkaloid content. Factors such as UV light and the presence of fungi in the rhizosphere are among myriad elements contributing to the overall biochemistry of plants. Environmental factors impact both primary and secondary metabolism, and strong evidence suggests that plant responses are highly coordinated [17, 70]. The production of alkaloids as defense metabolites is likely underpinned by biochemical events that can only be visualized through broad-scope metabolite profiling. Elucidation of these complex mechanisms depends on persistent and iterative metabolomics studies, which in turn rely on continuously improving analytical technologies.

## Methods

### Plant material and tissue preparation

Selected tissues were harvested from *Hydrastis canadensis*, *Sanguinaria canadensis*, *Nigella sativa*, *Mahonia aquifolium*, *Menispermum canadense*, *Stylophorum diphyllum*, and *Xanthoriza simplicissima* plants cultivated outdoors at the Jardin Botanique de Montréal (Montréal, Québec; http://espacepourlavie.ca). *Jeffersonia diphylla* and *Berberis thunbergii* plants were purchased from Plant Delights Nursery (Raleigh, North Carolina; www.plantdelights.com) and Sunnyside Greenhouses (Calgary, Alberta; www.sunnysidehomeandgarden.com), respectively. *Chelidonium majus*, *Papaver bracteatum*, *Argemone mexicana*, *Eschscholtzia californica*, *Nandina domestica*, *Glaucium flavum*, *Thalictrum flavum* and *Corydalis chelanthifolia* were grown from seed germinated in potted soil under standard open air greenhouse conditions. Seeds were obtained from B and T World Seeds (http://b-and-t-world-seeds.com) with the exception of *T. flavum* and *P. bracteatum*, which were obtained from Jelitto Staudensamen (www.jelitto.com) and La Vie en Rose Gardens (www.lavieenrosegardens.com), respectively. Callus cultures of *Cissampelos mucronata*, *Cocculus trilobus*, and *Tinospora cordifolia* were purchased from Deutsche Sammlung von Mikroorganismen und Zellkulturen (DSMZ, Braunschweig, Germany; http://www.dsmz.de) and maintained as described previously [13]. Tissues were flash-frozen in liquid nitrogen and stored at −80 °C. Four biological replicates were processed per species for all analyses except targeted HPLC-MS/MS, in which case only one replicate was performed. Tissues for each biological replicate were lyophylized, ground to a fine powder, and partitioned for extraction using water (NMR, HPLC-UV analyses), organic solvent (LC/DI-MS/MS) or methanol (UPLC-FTMS).

### NMR spectroscopy

Lyophylized tissue powder was extracted three times with water, and reduced to dryness. This dry, water-soluble fraction was reconstituted in 600 μL sodium phosphate buffer (50 mM, pH 7). Seventy microliters D_2_O and 30 μL of a standard buffer solution [3.73 mM DSS (disodium 2,2-dimethyl-2-silapentane-5-sulfonate), 0.47 % (w/v) sodium azide] were added. Samples were vortexed for 1 min, sonicated for 30 min, and transferred to a standard Shigemi microcell NMR tube. All ^1^H-NMR spectra were acquired on a 500 MHz Inova (Varian Inc., Palo Alto, California) spectrometer equipped with a 5-mm HCN Z-gradient pulsed-field gradient (PFG) cold probe. Data was collected at 25 °C using the first transient of the NOESY-presaturation pulse sequence, which was chosen for its high degree of quantitative accuracy. Spectra were collected with 256 transients using an 8-second acquisition time and a 1-second recycle delay.

### NMR compound identification and quantification

All FIDs (free induction decays) were zero-filled to 64 k data points and subjected to line broadening of 0.5 Hz. The singlet produced by DSS methyl groups was used as an internal standard for chemical shift referencing (set to 0 ppm) and for quantification purposes. All ^1^H-NMR spectra were processed and analyzed using the Chenomx NMR Suite Professional software package v. 6.0 (Chenomx Inc., Edmonton, Alberta). This software allows for qualitative and quantitative analysis of an NMR spectrum by manually fitting spectral signatures from an internal database of reference spectra to the full NMR spectrum [59]. The spectral fitting for each metabolite was done using the standard Chenomx 500 MHz metabolite library. For most samples, 90 % of all visible peaks were assigned to a compound and more than 90 % of the spectral area could be routinely fit using the Chenomx spectral analysis software. Most of the visible peaks were annotated with a compound name. It was shown previously that this fitting procedure provides absolute concentration accuracies of 90 % or better [59]. Each spectrum was processed and analyzed by at least two NMR spectroscopists to minimize compound misidentification and misquantification. Where necessary, sample spiking was employed to confirm the identities of assigned compounds. Sample spiking involved the addition of 20–200 μM of the candidate compound followed by observations of whether NMR signal intensities changed as expected.

### DFI-MS/MS analysis

Lyophilized tissue powder was processed for targeted quantitative metabolomics analysis using the commercially available kit AbsoluteIDQ 150 (Biocrates Life Sciences AG, Innsbruck, Austria). This kit assay employs direct flow injection (DFI)-MS/MS technique, and is specifically adapted for an ABI 4000 QTrap (Applied Biosystems/MDS Sciex, Foster City, California). Using this method, up to 163 different metabolites, including amino acids, acylcarnitines, glycerophospholipids, sphingolipids, and sugars can be identified and quantified. The kit assay relies on selective derivitization and extraction, and resulting analytes are profiled using pre-defined multiple reaction monitoring (MRM) pairs, neutral loss measurements and precursor ion scans. Isotope-labeled internal standards are included as part of the kit plate filter for metabolite quantification. The AbsoluteIDQ 150 kit contains a 96 deep-well plate with a filter attachment, along with reagents and solvents used to prepare the plate assay. For standardization and calibration purposes, the first 8 wells in the kit were used for the following: one blank, three zero samples, seven standards and three quality control samples. Plant samples were processed and analyzed according to manufacturer’s instructions. Briefly, lyophilized tissue was extracted with organic solvent and 10 μL of each organic fraction was loaded onto the center of the filter on the upper 96-well kit plate and dried with a stream of nitrogen. Subsequently, 20 μL of derivatizing agent 5 % phenylisothiocyanate was added. Following an incubation period, filter spots were dried again and subjected to extraction with 300 μL methanol containing 5 mM ammonium acetate. Extracts were obtained by centrifugation into the lower 96-well plate and diluted with kit MS running solvent. MS analysis was performed according to manufacturer instructions on an ABI 4000 QTrap (Applied Biosystems/MDS Sciex, Foster City, California). byDFIBiocrates MetIQ software was used to control assay workflow, from sample registration to automated calculation of metabolite concentrations to data export.

### HPLC-UV analysis

To determine polyphenol content, lyophilized plant tissue was subjected to extraction [31] and high performance liquid chromatography with (HPLC)-UV analysis [48] as described previously, with some modifications. Dried powder was homogenized in 8–10 mL of water and incubated in a boiling water bath for 30 min with intermittent vortexing. Extracts were cooled and centrifuged at 3000 rpm for 20 min. The extraction was repeated and supernatants were pooled and filtered through a 0.45 μm nylon membrane (EMD Millipore, Billerica, Massachusetts). Filtrate was lyophilized, dissolved in HPLC running buffer (Solvent A; 50 mM sodium phosphate pH 2.5) and passed once more through a 0.45 μm nylon filter prior to injection. Analysis was performed using an Agilent 1100 series HPLC system consisting of an Agilent G1311A quaternary pump equipped with an Agilent G1315B diode array detector (Agilent Technologies, Santa Clara, California). Separation was achieved using a Synergi RP-polar C18 column (Phenomenex, Torrance, California) and a gradient elution profile of Solvent A and Solvent B (100 % methanol) as follows: 0 min, 5 % B; 15 min, 30 % B, 40 min, 40 % B; 60 min, 50 % B; 65 min, 55 % B; 90 min, 100 % B. Flow rate was 1.0 mL/min and injection volume was 40 μL. Absorbance was monitored at 254, 280, 306 and 340 nm. Phenolic compounds were identified by comparison of retention time (Rt) and UV spectral data with those of known standards and quantification was achieved using routine procedures based on calibration curves.

### UPLC-FTMS analysis

Lyophilized plant tissue was extracted twice with methanol, and supernatants were pooled and re-lyophilized. Residues were precisely weighed (~4-5 mg each sample) and dissolved in 20 % methanol to a concentration of 5.0 mg/mL with the aid of vortex mixing and sonication. Each solution was then diluted 1:5 with 5 % methanol and centrifuged at 10,000 g to remove insoluble matter. A Dionex Ultimate 3000 RSLC ultrahigh-performance liquid chromatography (UPLC) system coupled to a Thermo LTQ-Orbitrap Velos mass spectrometer (MS) equipped with heated electrospray ionization (ESI) source was used. The plant metabolites were separated on a BEH C_18_ UPLC column (2.1 × 100 mm, 1.7 μm, 130 Å). The mobile phase was 0.01 % formic acid in water (solvent A) and 0.01 % formic acid in isopropanol (solvent B) for binary gradient elution. The gradient was 2 % to 100 % B over 16 min; 100 % B for 2 min before the column was equilibrated for 4 min between injections. The column flow rate was 0.3 mL min^−1^ and the column temperature was set to 40 °C. The injection volume was 10 μL. MS detection was in the Fourier transform (FT) full mass-scan mode (i.e. FTMS) within a range of *m*/*z* 100 to 1000. The mass resolution was set at 30,000 FHMW and the automatic gain control (AGC) target was 1×10^6^ with an allowable maximum injection time of 500 ms. Two UPLC-FTMS runs per sample were performed in each of the positive-ion and negative-ion detection modes, and the LC-MS data files were recorded in centroid mode. To ensure mass accuracy, real-time internal mass calibration was applied in addition to standard external calibration procedures throughout all runs by using two reference masses from two ubiquitous background ions, i.e., *m*/*z* 391.28429 from the (M + H)^+^ ion of bis(2-ethylhexyl) phthalate for the (+) ion mode detection and *m*/*z* 112.98563 from the (2 M + Na-2H)^−^ ion of formic acid in (−) ion mode detection. In this way, all the measured mass errors, as checked, were within ±2.5 ppm. Typical ESI parameters were as follows: ion source spray voltage, 3500 V for (+) ESI and 3000 V for (−) ESI; sheath gas flow 40 arbitrary units (AU); auxiliary gas flow 15 AU; heated nebulizer temperature 350 °C; and capillary temperature 325 °C. The (+) and (−) ion mode datasets from each sample were respectively processed with the freely available XCMS suite [52, 55] downloadable at http://xcmsonline.scripps.edu/ for automatic peak extraction, retention time shift correction, peak grouping and alignment. The detected metabolites from 4 samples for each plant species were saved in individual CSV peak tables in the format of mono-isotopic *m*/*z* values, and retention times (R_t_ in min) versus corresponding peak areas (ion counts), for detected metabolites across samples. Manual peak de-isotoping was applied for each of the resultant peak tables and those chemical and electronic noises were also removed during this step. Positive ion mode mass lists were mined for ionic masses (*m*/*z*) corresponding to those predicted for known BIAs based on established empirical formulae. Matching observed ionic masses with predicted BIA ionic masses was performed using an allowable error range of ± 2.0 ppm.

### Triple quadrupole LC-MS/MS analysis

LC-MS/MS was performed using a previously described method [13] with minor modifications. Briefly, tissue was extracted with Bieleski’s solution (15:1:4 methanol:formic acid:water, v:v) and centrifuged at 14 000 g for 10 min at 4 °C. Supernatant was filtered through 22 μm Millex filters (EMD Millipore, Billerica, MA), lyophylized and reconstituted (5 mg/mL) in Solvent A (10 mM ammonium acetate, 5 % acetonitrile, pH 5.5). Dilutions of 1:10 and 1:100 were prepared for LC-MS/MS analysis, which was performed using an Agilent 1200 series HPLC coupled to an Agilent 6410B triple quadrupole MS analyzer (Agilent, Santa Clara, California). Separations were achieved with a Zorbax Eclipse Plus C18 column at a flow rate of 0.5 ml/min using the following gradient of Solvent A and Solvent B (100 % acetonitrile): 0 min, 100 % Solvent A; 10 min, 50 % Solvent A; 12 min, 1 % Solvent A; 13 min, 1 % Solvent A. Eluent was introduced to the MS operating in positive ion mode via an ESI source. Source and interface conditions were optimized for BIAs (gas temperature 350 °C, gas flow rate 10 L/min; nebulizer gas pressure 50 psi, fragmentor 100 V, capillary 4000 V). Quadrupole 1 and 2 were set to RF only with quadrupole 3 scanning from 200–700 *m*/*z*. These wide scans were used to select BIAs whose identities could be confirmed by comparing 1) retention times and 2) collision-induced dissociation (CID) spectra with those of authentic standards. For CID spectra, 25 eV was applied to quadrupole 2 and resulting fragments were detected in quadrupole 3 by scanning from *m*/*z* 40 to 2 units greater than the precursor ion *m*/*z*.

### Multivariate analysis

To compare metabolite compositions and concentration differences between samples derived from different plant species, principal component analysis (PCA) was performed using MetaboAnalyst v. 2.0 [65], a web-based metabolomics data processing tool which accepts a wide variety of input data such as NMR or MS peak lists and compound/concentration information (http://www.metaboanalyst.ca/MetaboAnalyst/). Metabolite quantities formed the basis for PCA of NMR, LC/DFI-MS/MS, and HPLC-UV data. Relative abundances (i.e. ion counts) of ionic masses (*m/z)* corresponding to those predicted for known BIAs formed the basis for PCA of extracted UPLC-FTMS data. Data treatment was performed essentially as described previously [15]. Briefly, data normalization was performed using MetaboloAnalyst’s built-in normalization protocols [65] including row-wise and column-wise normalization and auto-scaling procedures. These original variables were summarized into much fewer variables (scores) using their weighted averages (loadings). Graphical summaries were provided as two-dimensional scores and loadings plots, respectively.

## Additional files

Additional file 1:
**Phylogenetic relationships among the Ranunculales as evidenced by molecular loci and morphological data.** Adapted from [58]. (PDF 602 kb) Additional file 2:
**Selected examples of BIA structural subgroups derived from the basic benzylisoquinoline subunit.** (PDF 1198 kb) Additional file 3:
**Metabolites detected and quantified by NMR-based profiling.** Compound abundances (mg/g dry weight) are provided for each of 4 replicates (labeled A, B, C, D) for all 20 plant species. Means ± SD are provided for each metabolite. Species abbreviations are defined in Table 1. (XLS 213 kb) Additional file 4:
**Metabolites detected and quantified by LC/DFI-MS/MS-based profiling.** Compound abundances are provided for each of 4 replicates (labeled A, B, C, D) for all 20 plant species. Means and standard deviations (SD) are provided for each metabolite. Species abbreviations are defined in Table 1. A complete listing of full compound names and abbreviations is available online: http://www.biocrates.com/products/research-products/absoluteidq-p150-kit. (XLS 317 kb) Additional file 5:
**Two-dimensional principal component analysis (PCA) of metabolite quantities obtained using LC/DFI-MS/MS-based profiling.** Results are presented as scores (A) and loadings (B) plots. The percent variance accounted for by each principal component (PC) is indicated. For the scores plot, each dot represents a one of four replicates analyzed per plant species. Areas enclosed by 95 % confidence ellipses, containing dots of the same color, define statistically significant class separations [34]. Species abbreviations are defined in Table 1. Loadings representing individual metabolites are shown as black dots (B). Metabolites are indicated for select loadings. A complete listing of loadings data is found in Additional file 16. Abbreviations: C, acylcarnitine; SM, sphingomyelin; PC, phosphatidylcholine; aa, diacyl; ae, acyl-ester. A complete listing of full compound names and abbreviations is available online: http://www.biocrates.com/products/research-products/absoluteidq-p150-kit. (PDF 1591 kb) Additional file 6:
**Metabolites detected and quantified by HPLC-UV-based profiling.** Compound abundances (mg/g dry weight) are provided for each of 4 replicates (labeled A, B, C, D) for all 20 plant species. Means ± SD are provided for each metabolite. Species abbreviations are defined in Table 1. (XLS 218 kb) Additional file 7:
**Two-dimensional principal component analysis (PCA) of metabolite quantities obtained using HPLC-UV-based profiling.** Results are presented as scores (A) and loadings (B) plots. The percent variance accounted for by each principal component (PC) is indicated. For the scores plot, each dot represents a one of four replicates analyzed per plant species. Areas enclosed by 95 % confidence ellipses, containing dots of the same color, define statistically significant class separations [34]. Species abbreviations are defined in Table 1. Loadings representing individual metabolites are shown as black dots (B). Metabolites are indicated for all loadings. Complete loadings data is provided in Additional file 16. (PDF 1167 kb) Additional file 8:
**Individual metabolite quantities determined by HPLC-UV analysis for 20 BIA-accumulating plant species.** Plant species are designated by number, as defined in Table 1. Means ± SD were calculated using 4 replicates per species. (PDF 848 kb) Additional file 9:
**Exact mass list acquired using UPLC-FTMS analysis in positive ion mode.** UPLC retention time (R_t_) and total ion count is provided for each ionic mass (*m*/*z*) for all 4 replicates (labeled A, B, C, D) for each of 20 plant species. Species abbreviations are defined in Table 1. (XLSX 644 kb) Additional file 10:
**Exact mass list acquired using UPLC-FTMS analysis in negative ion mode.** UPLC retention time (R_t_) and total ion count is provided for each ionic mass (*m*/*z*) for all 4 replicates (labeled A, B, C, D) for each of 20 plant species. Species abbreviations are defined in Table 1. (XLSX 1216 kb) Additional file 11:
**Compilation of exact masses, acquired using UPLC-FTMS operating in positive ion mode, corresponding to empirical formulae of known benzylisoquinoline alkaloids (BIAs).** UPLC retention time (R_t_) and total ion count is provided for each putative BIA ionic mass (*m*/*z*) for all 4 replicates (labeled A, B, C, D) for each of 20 plant species. Species abbreviations are defined in Table 1. Each ionic mass (*m*/*z*) is putatively identified as an alkaloid, or group of alkaloids (‘mass group’) possessing an empirical formula matching the observed exact mass within an error range of ± 2.0 ppm. (XLSX 143 kb) Additional file 12:
**List of benzylisoquinoline alkaloids (BIAs), associated elemental compositions (i.e. empirical formula of singly charged ion) and theoretical ionic masses (either [M + H]** ^**+**^ **or [M]** ^**+**^ **).** In cases of structural isomerism wherein multiple BIAs possess the same composition and theoretical *m*/*z*, compounds are organized into ‘mass groups’. Only BIAs with *m*/*z* ranging from 270 to 430 are represented. (PDF 84 kb) Additional file 13:
**Triple quadrupole LC-MS/MS chromatographs representing 20 BIA-accumulating plant species.** Peak annotation was performed manually based on comparison with retention times (R_t_) and collision-induced dissociation (CID) spectra (Additional file 14) of authentic standards. Identified peaks are numbered in correspondence with those listed in Additional file 14. Species abbreviations are defined in Table 1. (PDF 1561 kb) Additional file 14:
**Compound names, ionic masses, retention times (R**_**t**_ **), structures and collision-induced dissociation (CID) spectral data acquired for authentic standards used to identify BIAs in plant samples analyzed by quadrupole LC-MS/MS.** (PDF 296 kb) Additional file 15:
**Selected examples of phthalideisoquinoline alkaloids.** (*R*,*S*)-Canadaline, a presumed precursor to certain phthalideisoquinoline alkaloids, is also shown. (PDF 1419 kb) Additional file 16:
**Principal Component Analysis (PCA) loadings data corresponding to metabolites quantified by NMR, LC/DFI-MS/MS, HPLC-UV, or UPLC-FTMS analyses.** Loadings for principal components (PC) 1 through 5 are shown. (XLS 114 kb)

## Abbreviations

BIA: Benzylisoquinoline alkaloid
CID: Collision-induced dissociation
DFI: Direct-flow injection
EI: Electrospray ionization
FTMS: Fourier-transform mass spectrometry
UPLC: Ultrahigh-performance liquid chromatography
ICR: Ion cyclotron resonance
LC: Liquid chromatography
MS: Mass spectrometry
NMR: Nuclear magnetic resonance
PCA: Principal component analysis
TOF: Time of flight
