# Supplementary figures and images for: Metabolome analysis of 20 taxonomically related benzylisoquinoline alkaloid-producing plants
Source: BMC Plant Biol. 2015 Sep 15;15:220. doi: 10.1186/s12870-015-0594-2 (PMC4570626; doi:10.1186/s12870-015-0594-2)

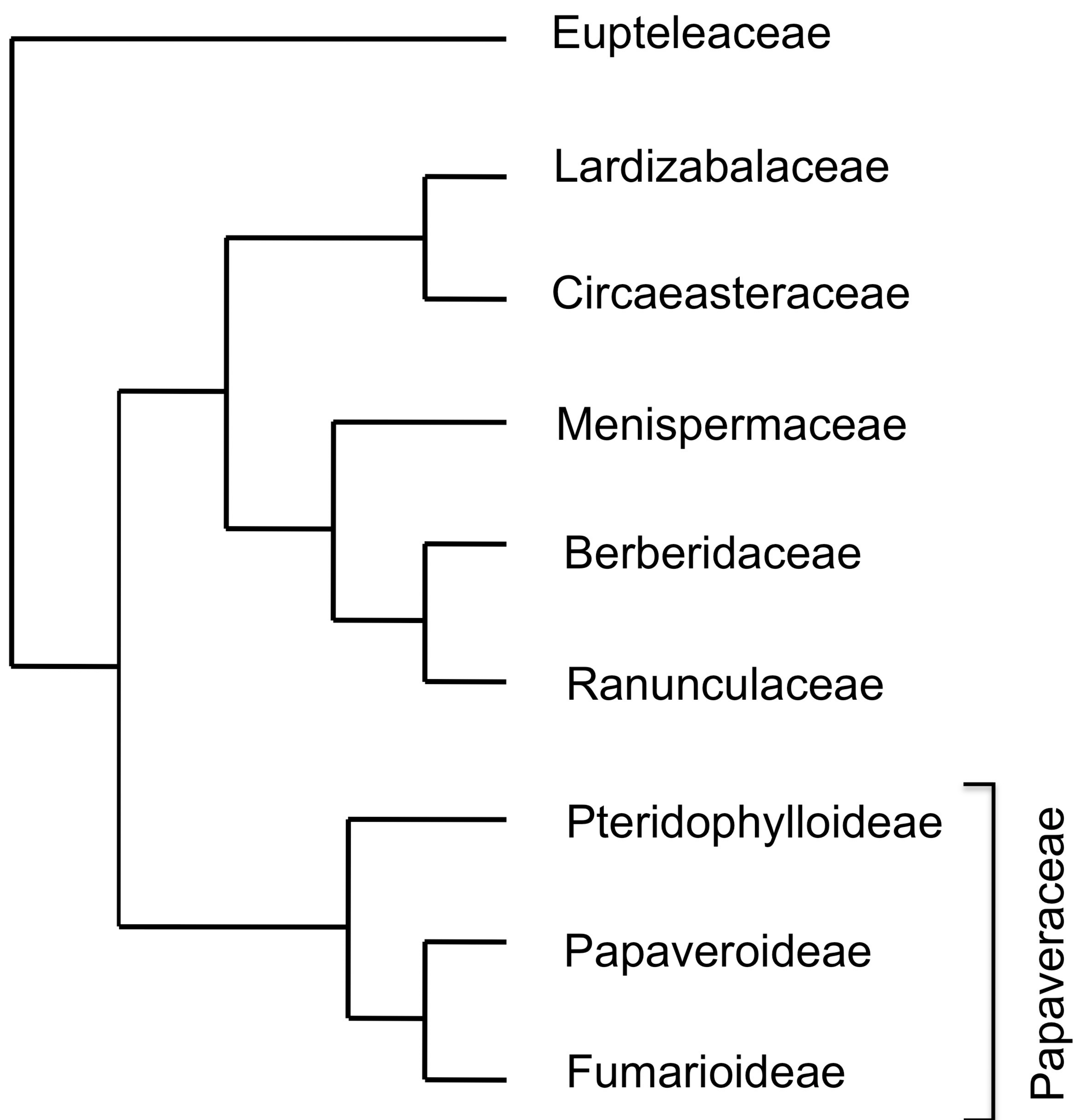

Supplement: Additional file 1: — Phylogenetic relationships among the Ranunculales as evidenced by molecular loci and morphological data. Adapted from [58]. (PDF 602 kb) [file 12870_2015_594_MOESM1_ESM.pdf]

A

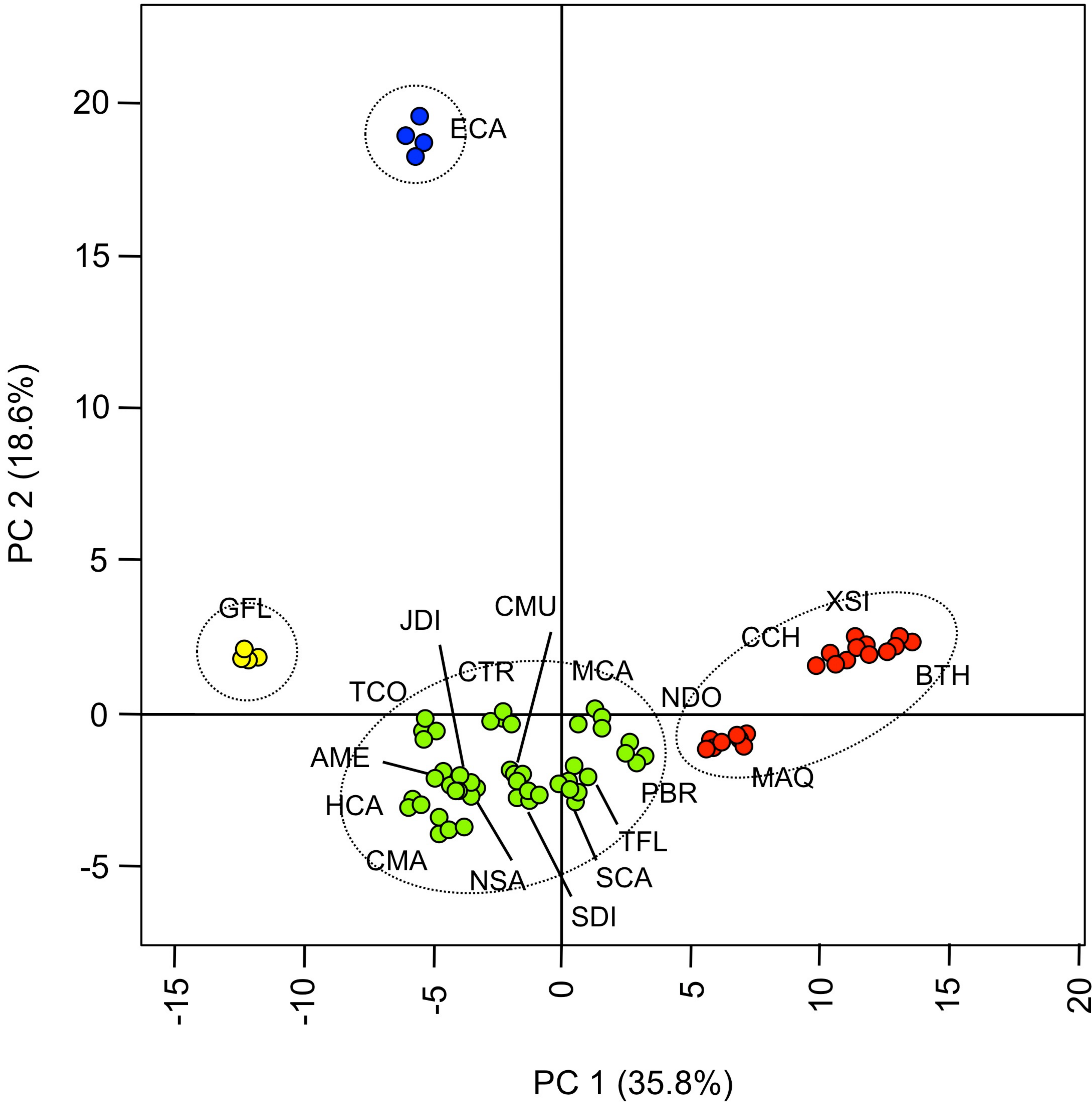

B

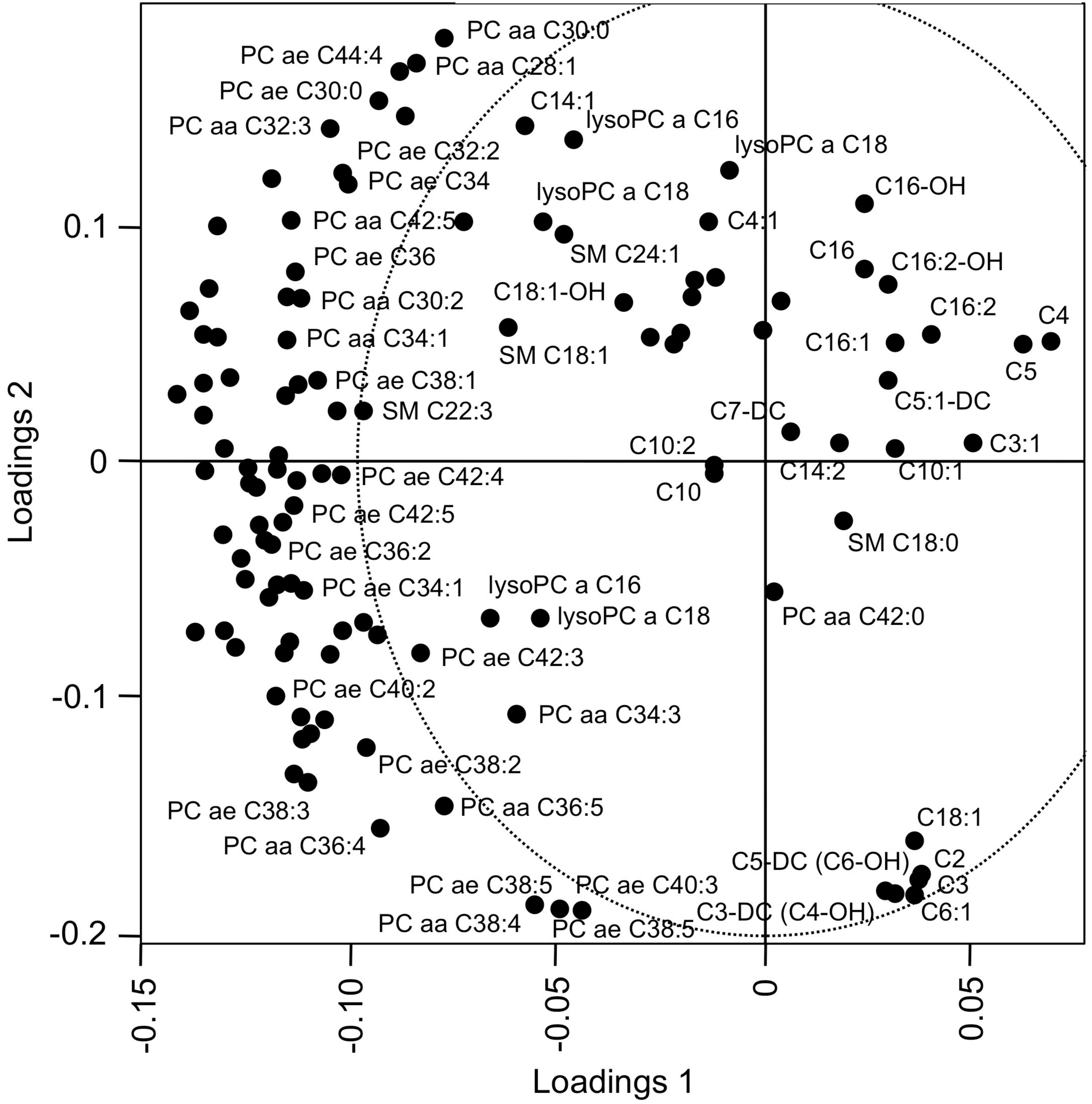

Supplement: Additional file 5: — Two-dimensional principal component analysis (PCA) of metabolite quantities obtained using LC/DFI-MS/MS-based profiling. Results are presented as scores (A) and loadings (B) plots. The percent variance accounted for by each principal component (PC) is indicated. For the scores plot, each dot represents a one of four replicates analyzed per plant species. Areas enclosed by 95 % confidence ellipses, containing dots of the same color, define statistically significant class separations [34]. Species abbreviations are defined in Table 1. Loadings representing individual metabolites are shown as black dots (B). Metabolites are indicated for select loadings. A complete listing of loadings data is found in Additional file 16. Abbreviations: C, acylcarnitine; SM, sphingomyelin; PC, phosphatidylcholine; aa, diacyl; ae, acyl-ester. A complete listing of full compound names and abbreviations is available online: http://www.biocrates.com/products/research-products/absoluteidq-p150-kit. (PDF 1591 kb) [file 12870_2015_594_MOESM5_ESM.pdf]

A

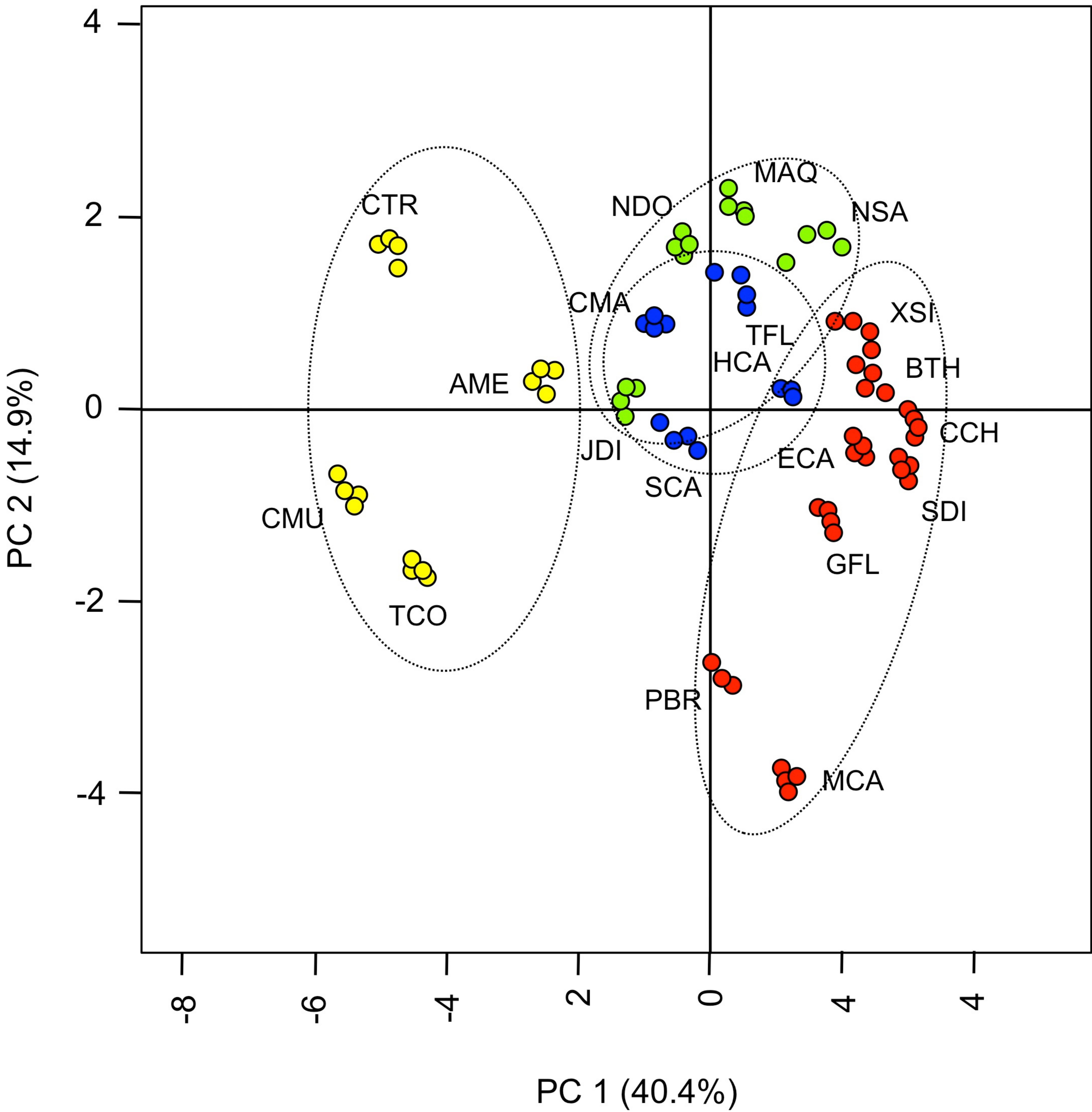

B

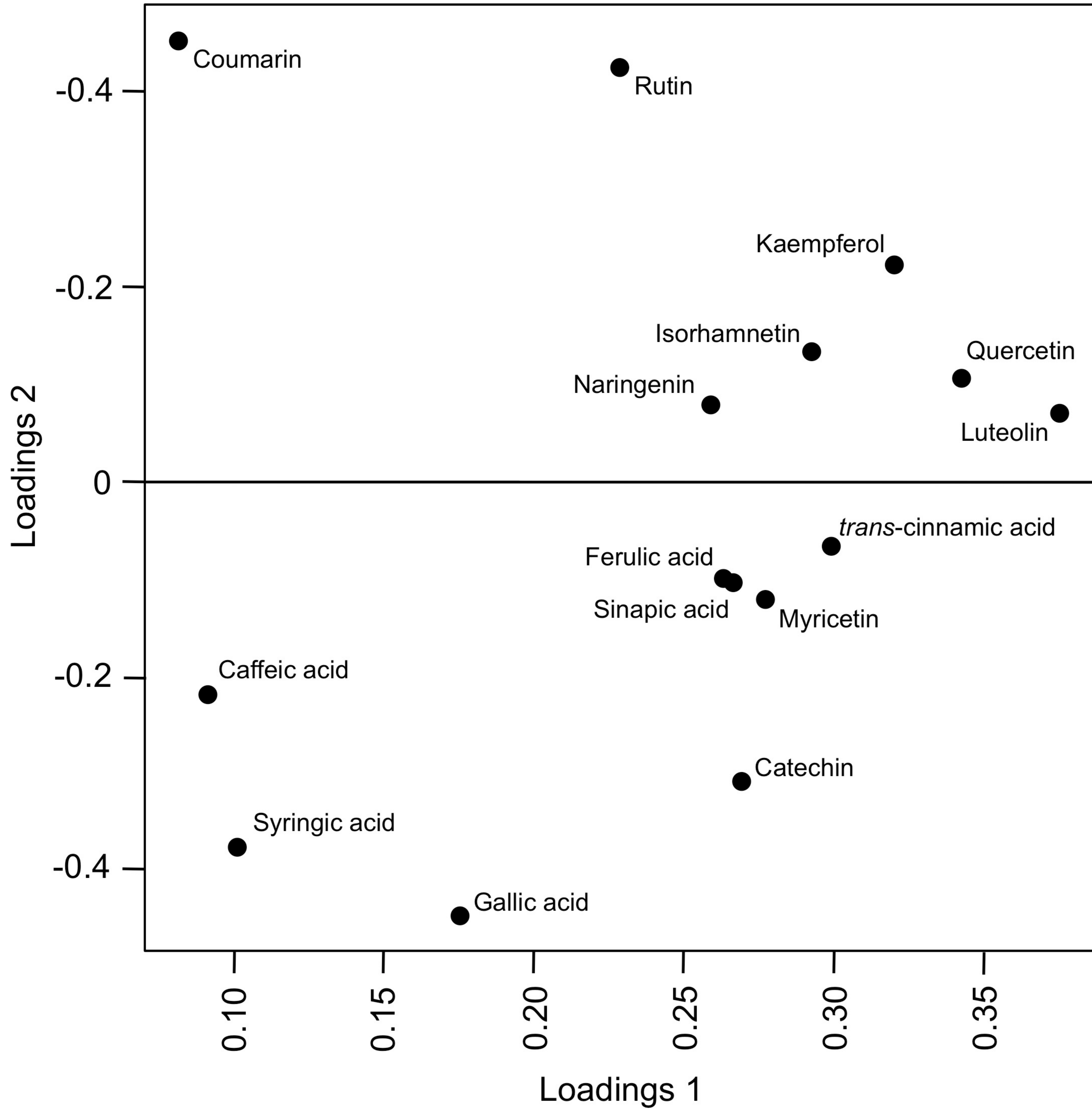

Supplement: Additional file 7: — Two-dimensional principal component analysis (PCA) of metabolite quantities obtained using HPLC-UV-based profiling. Results are presented as scores (A) and loadings (B) plots. The percent variance accounted for by each principal component (PC) is indicated. For the scores plot, each dot represents a one of four replicates analyzed per plant species. Areas enclosed by 95 % confidence ellipses, containing dots of the same color, define statistically significant class separations [34]. Species abbreviations are defined in Table 1. Loadings representing individual metabolites are shown as black dots (B). Metabolites are indicated for all loadings. Complete loadings data is provided in Additional file 16. (PDF 1167 kb) [file 12870_2015_594_MOESM7_ESM.pdf]

Content (mg g<sup>-1</sup> dry weight)

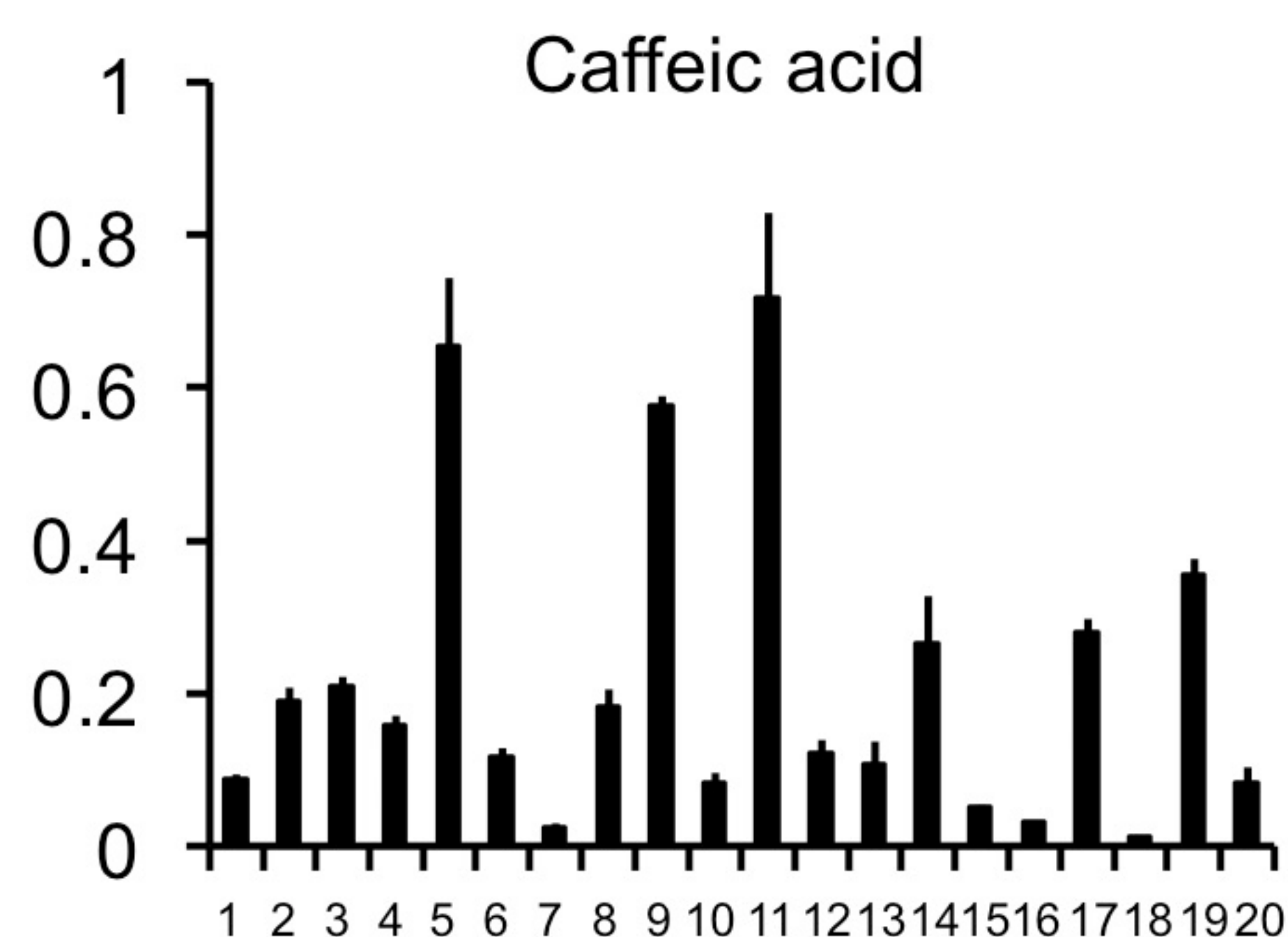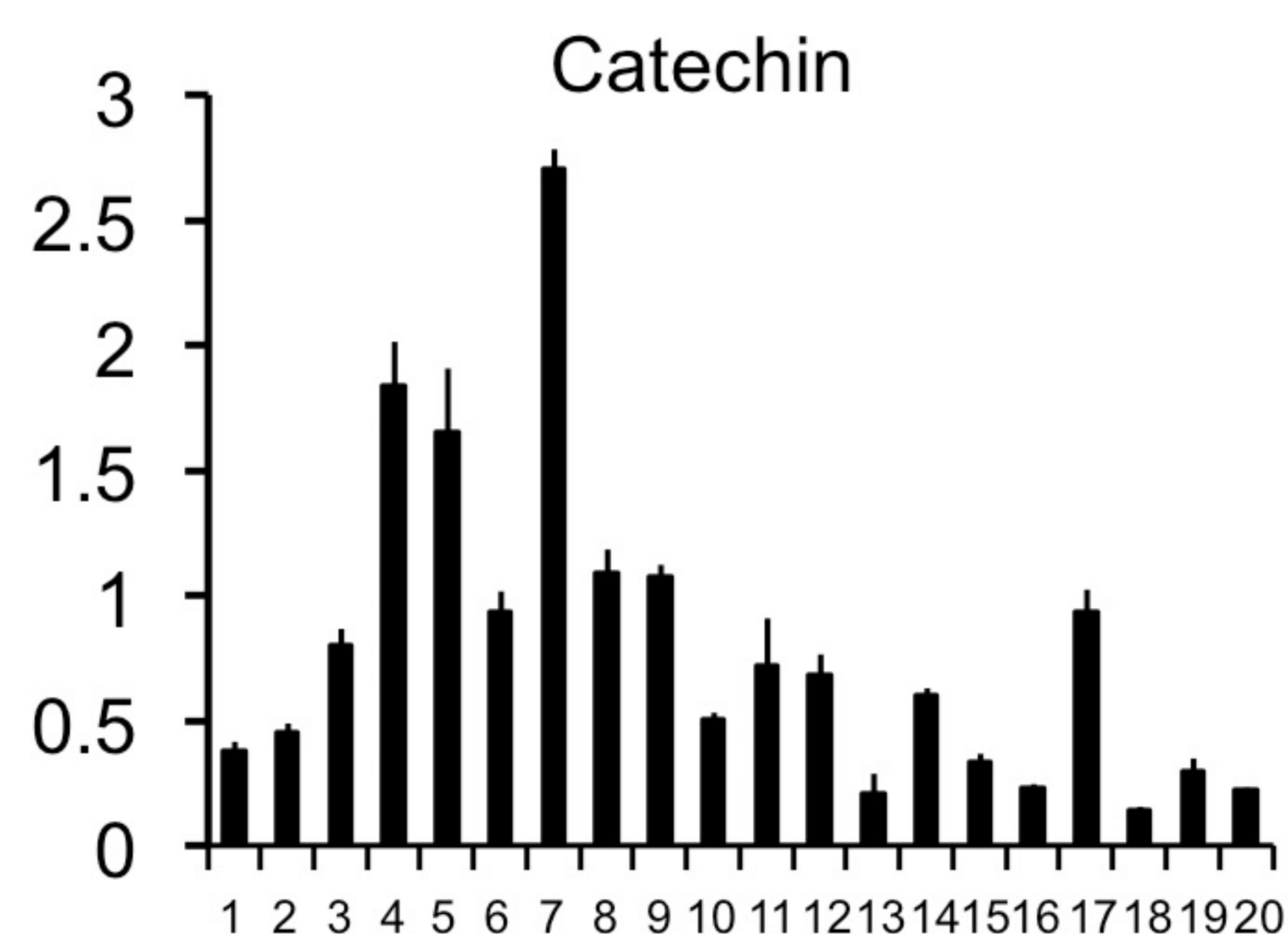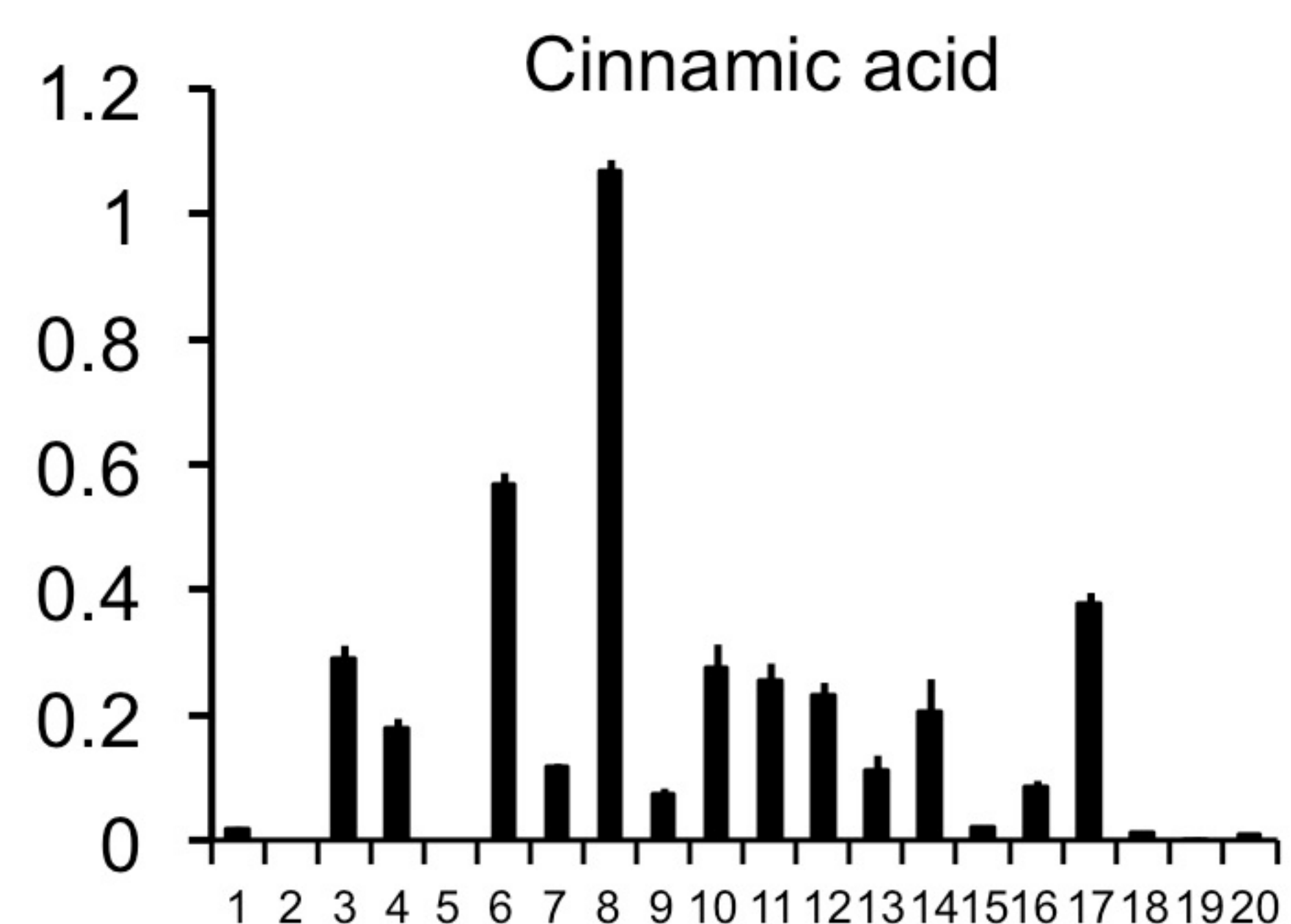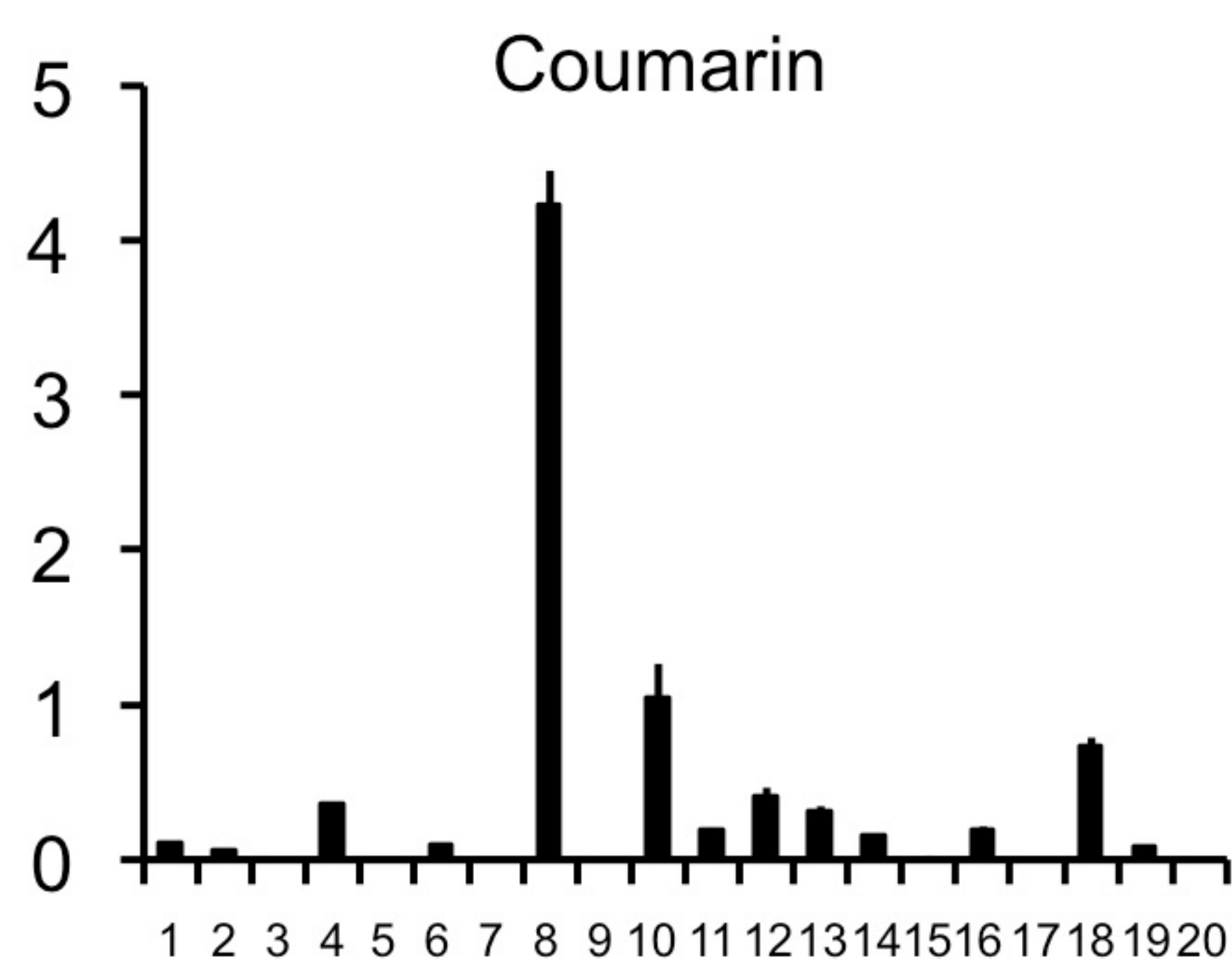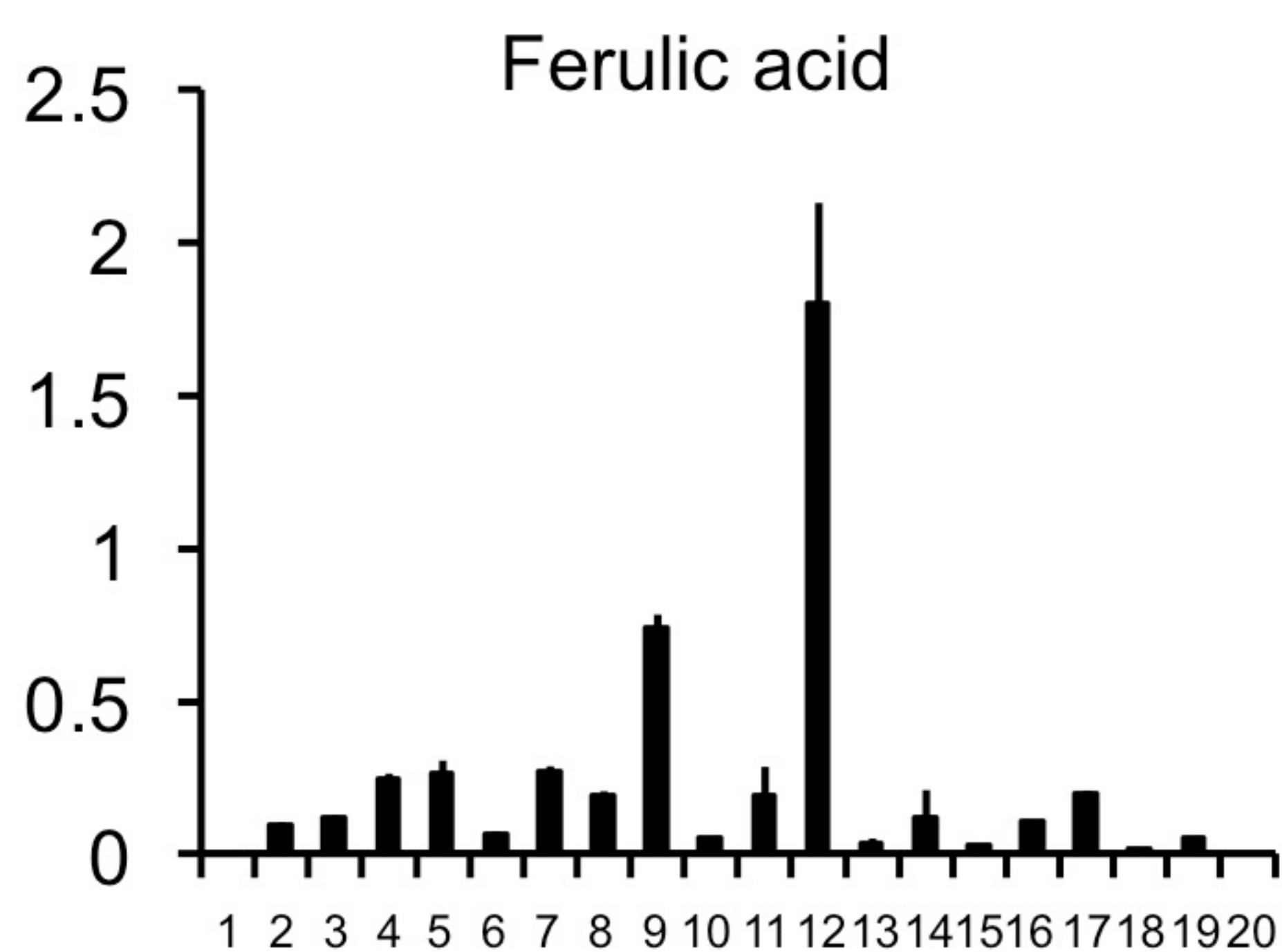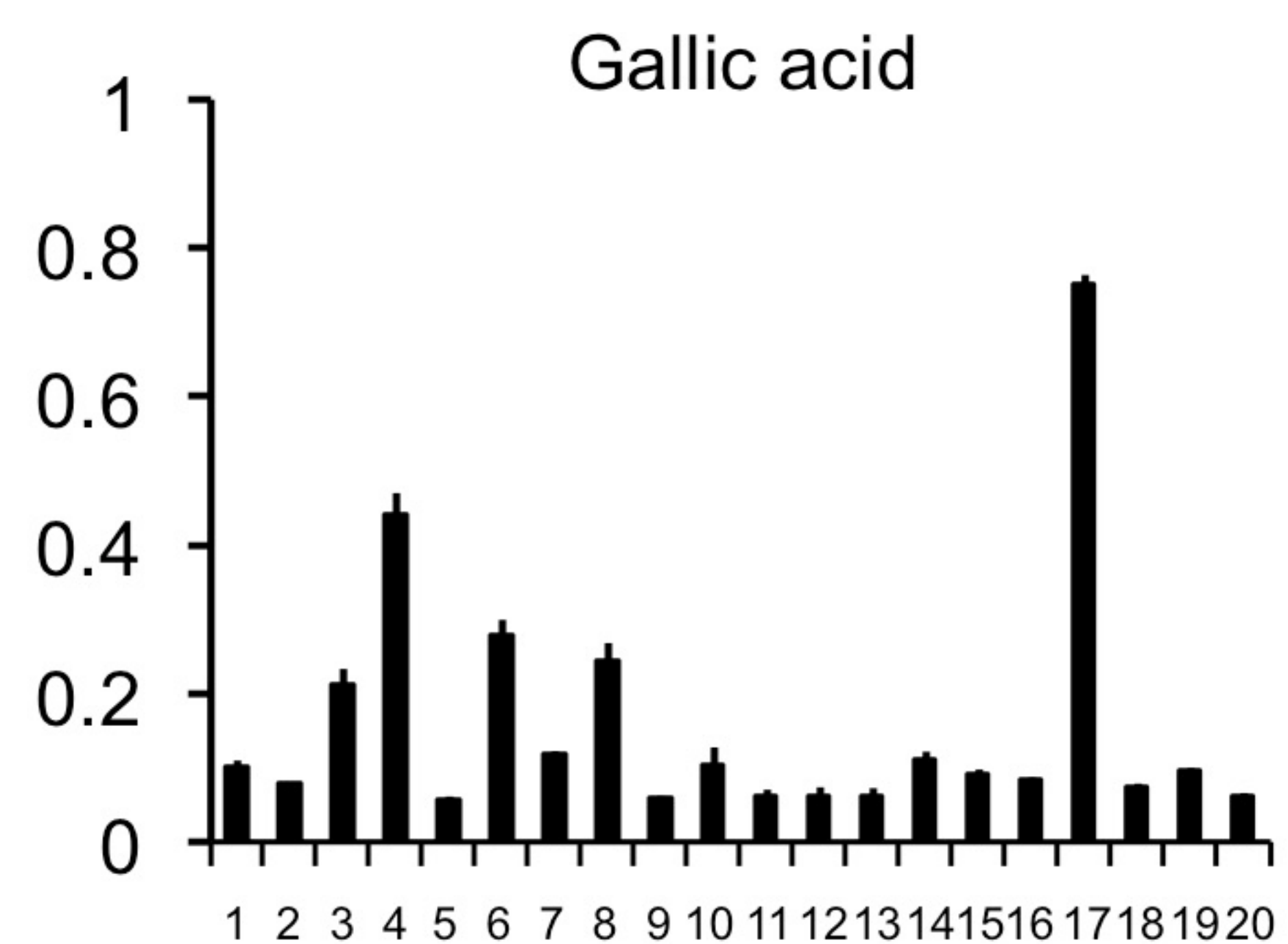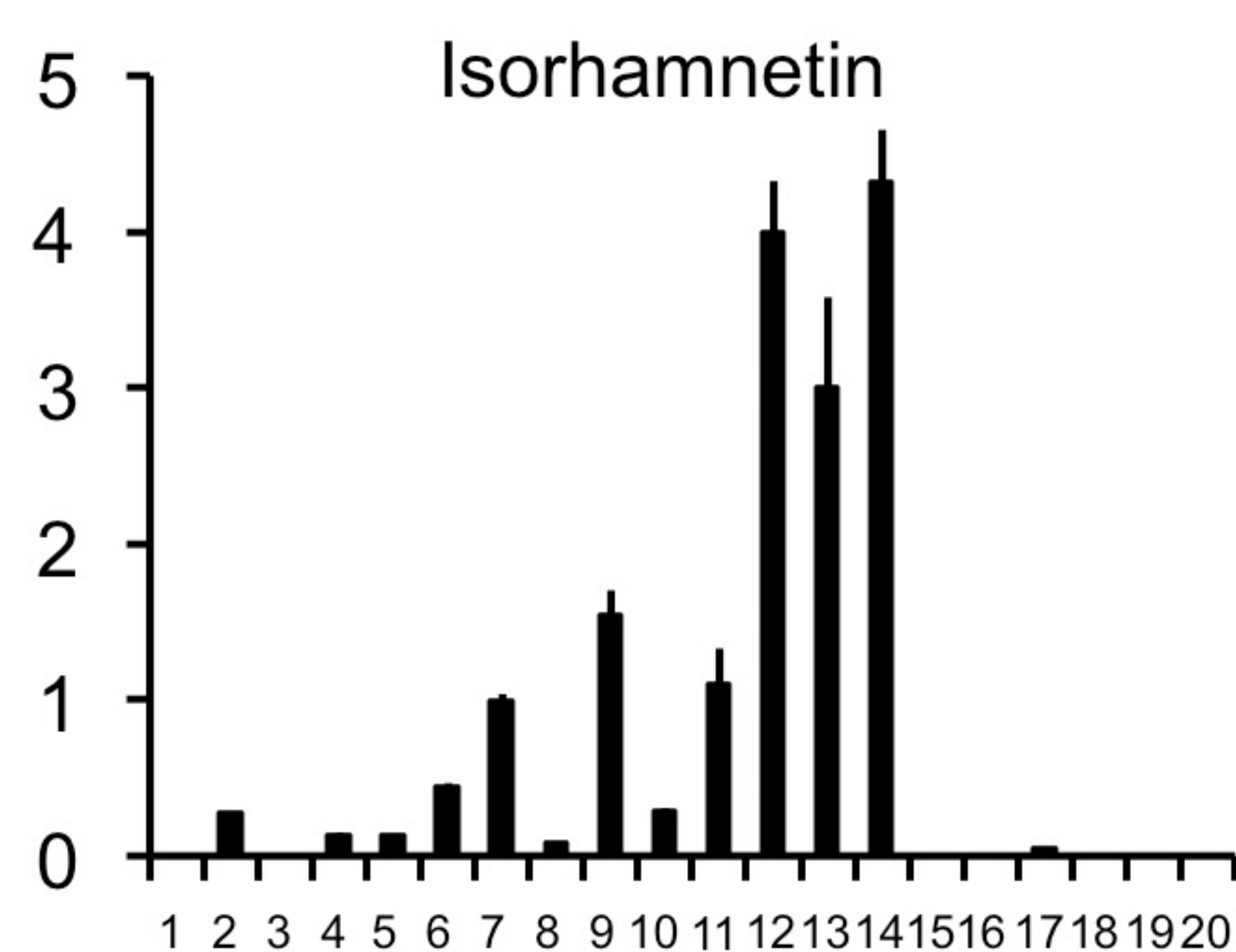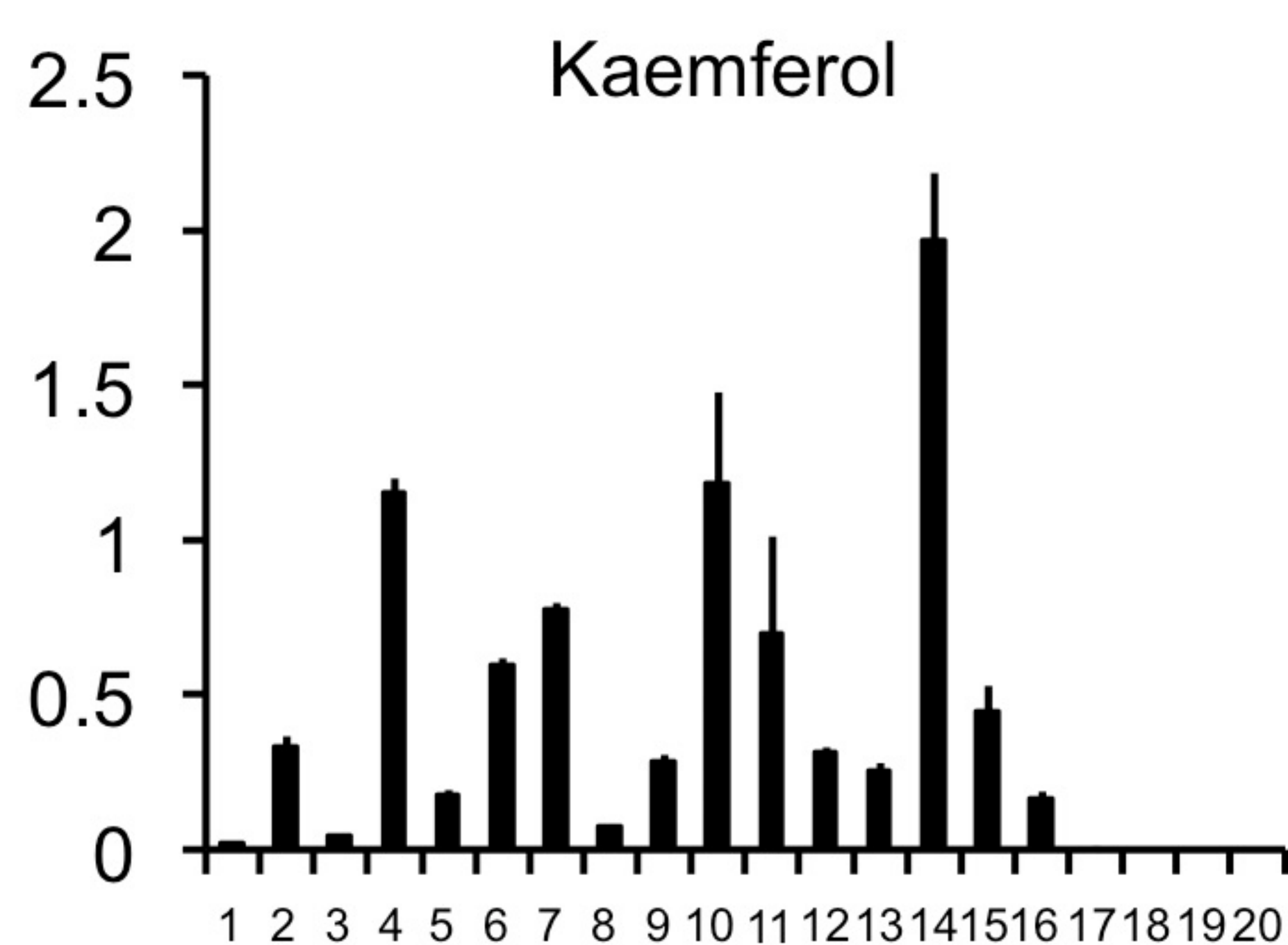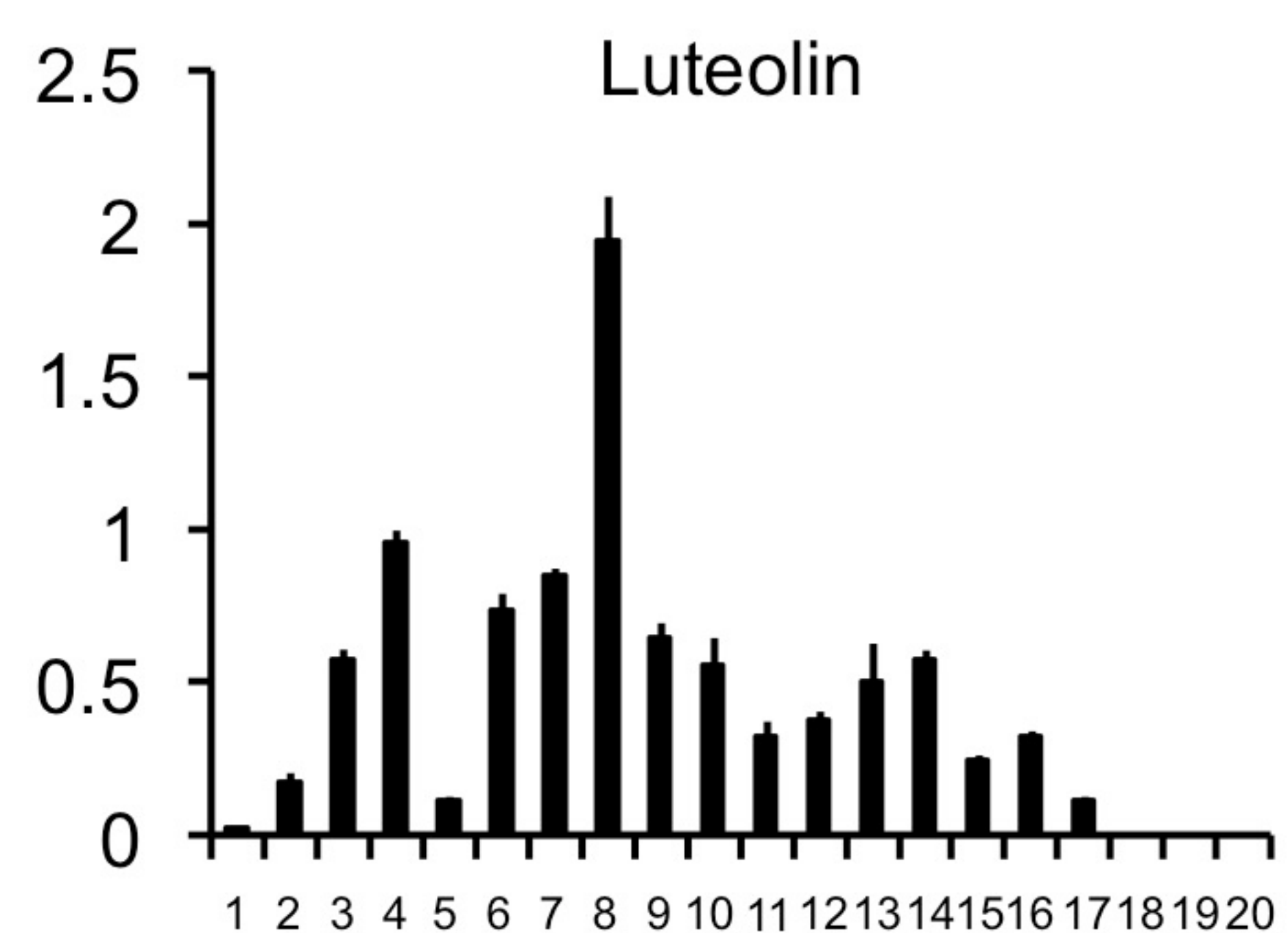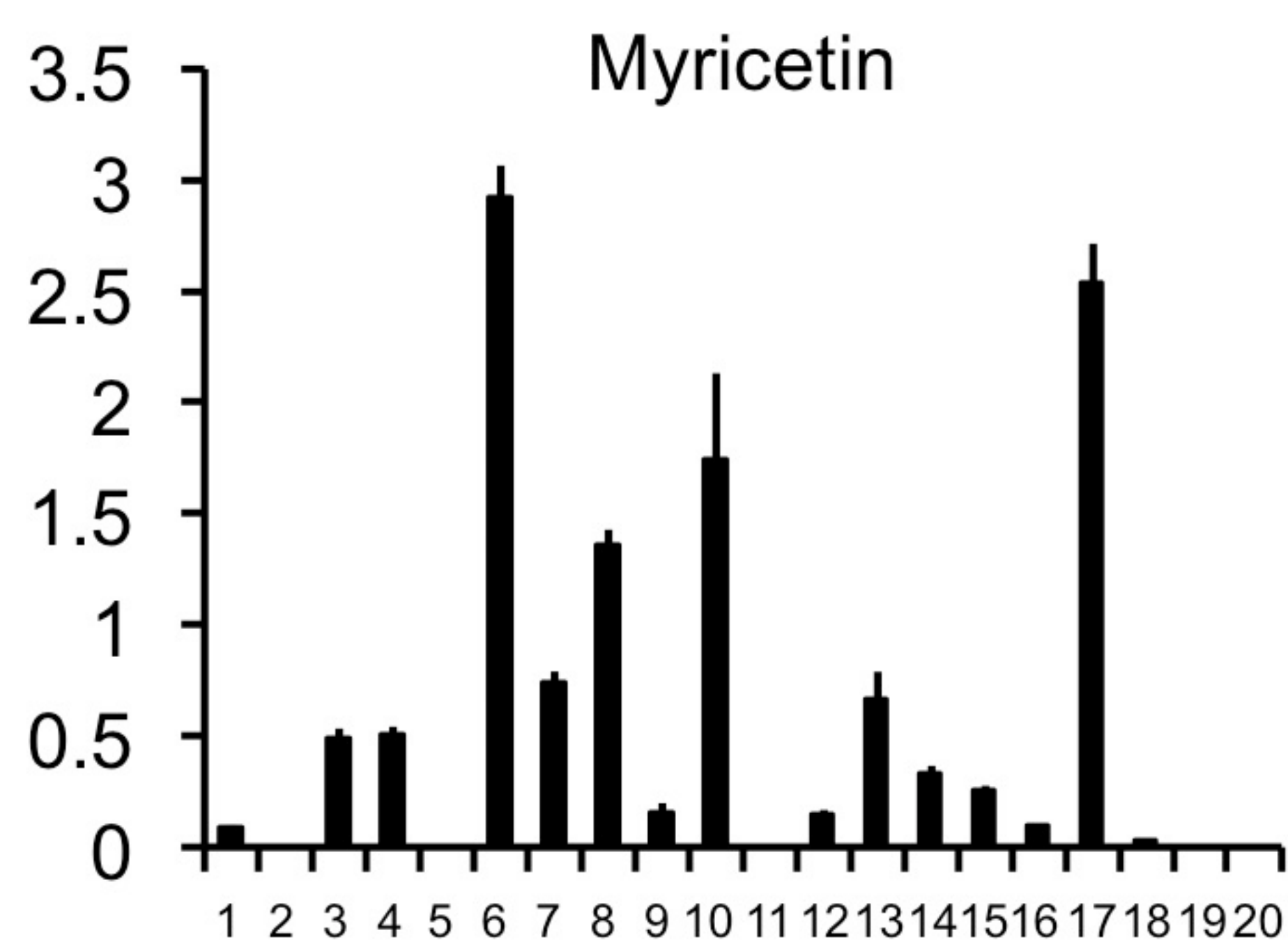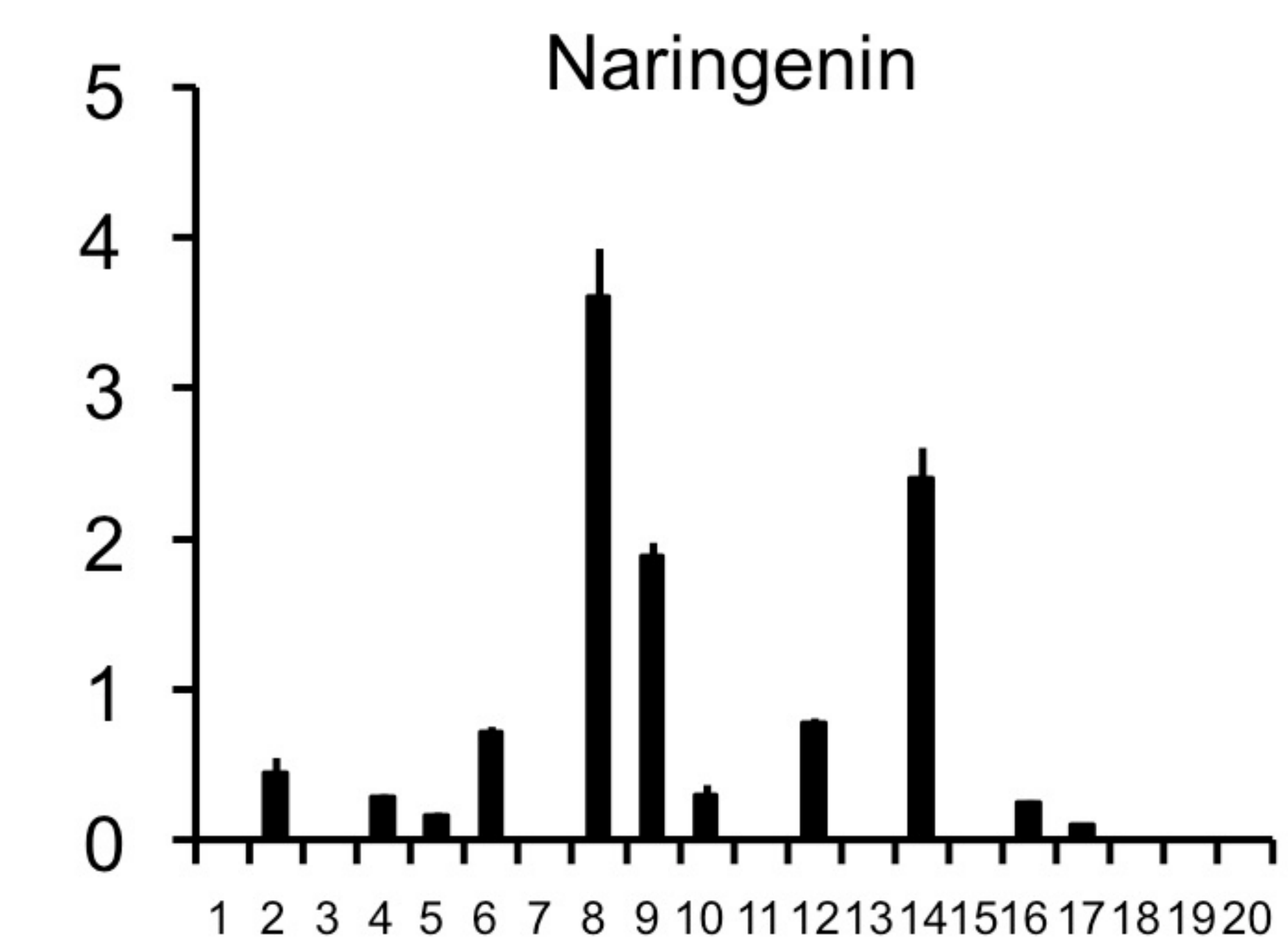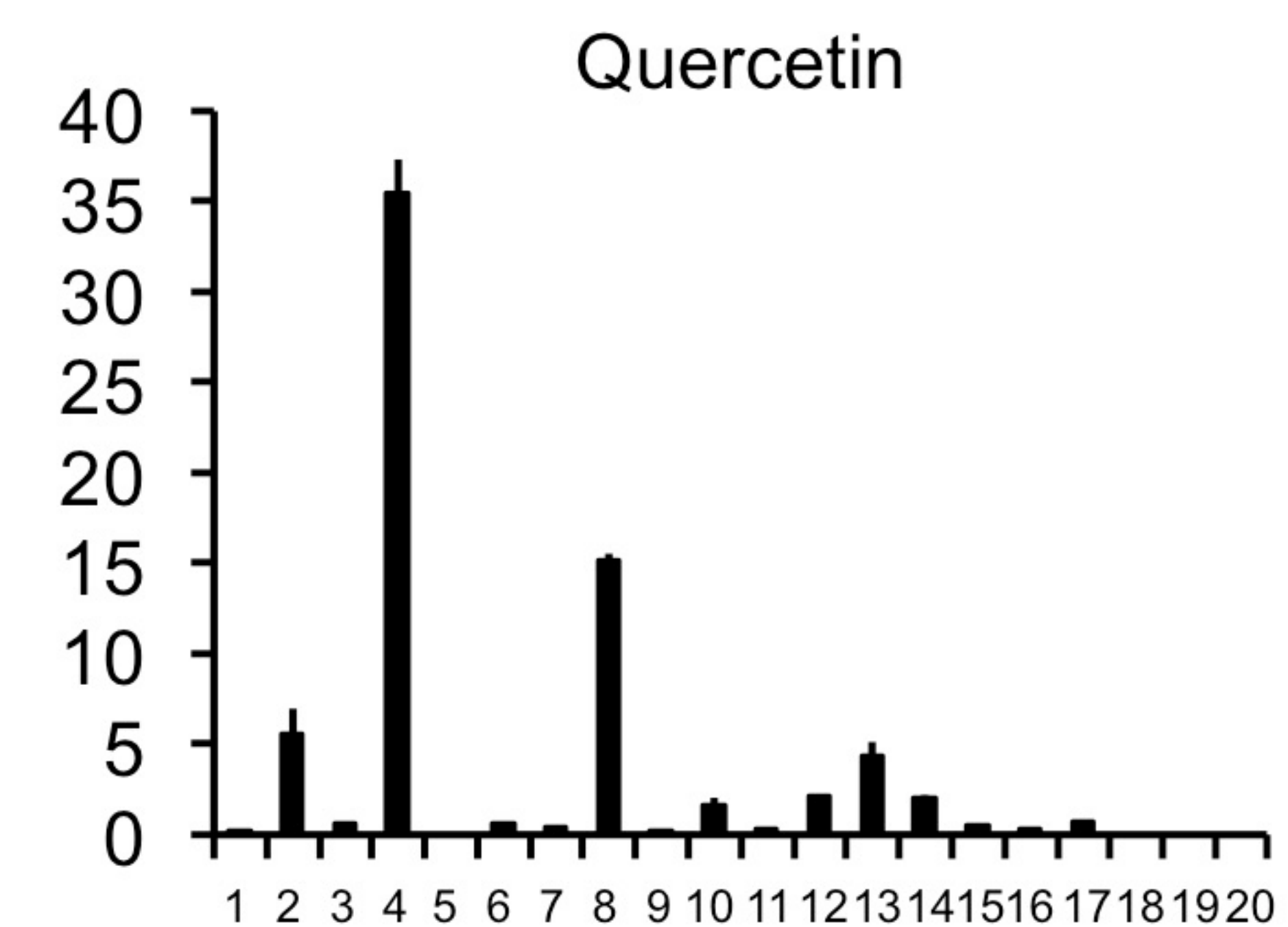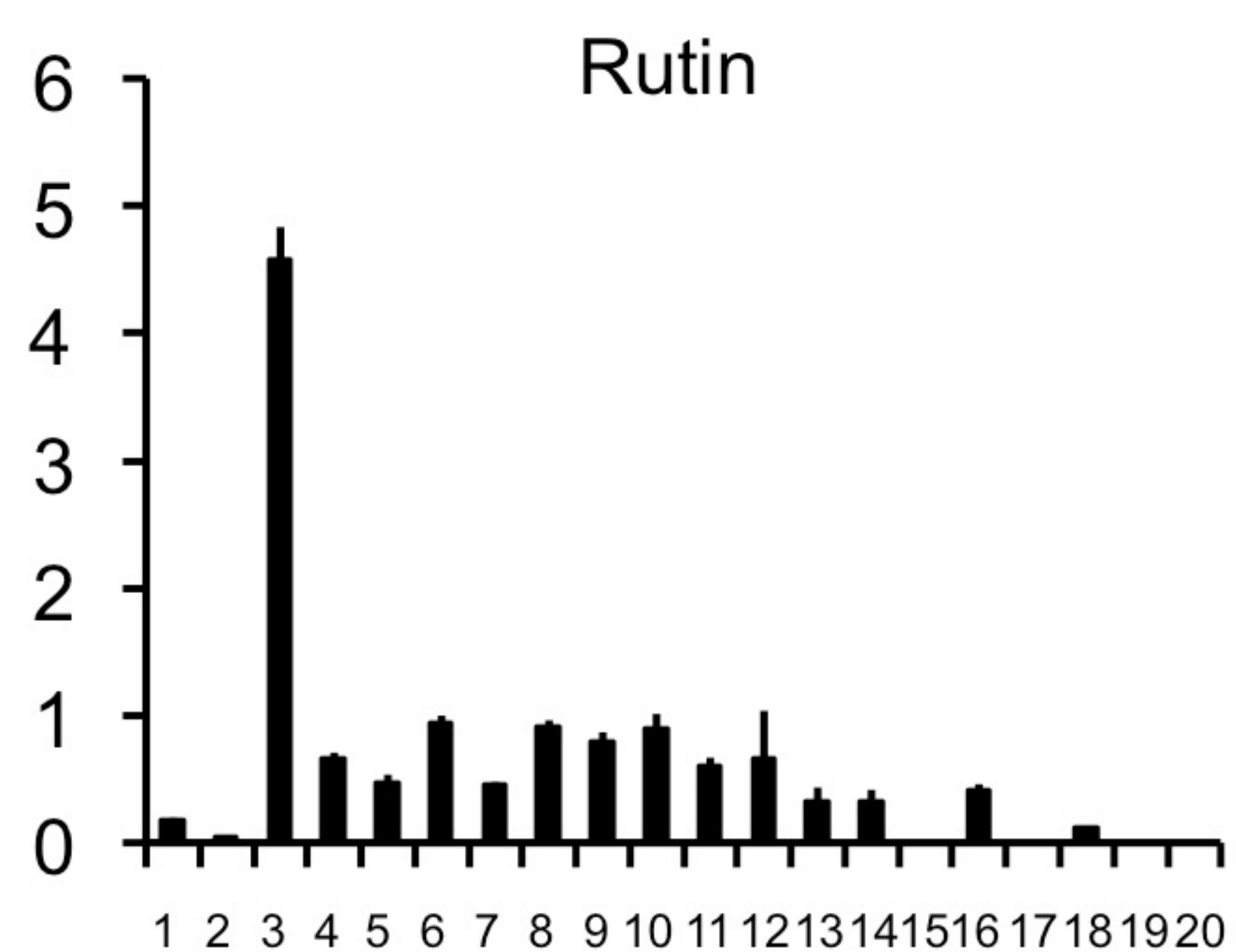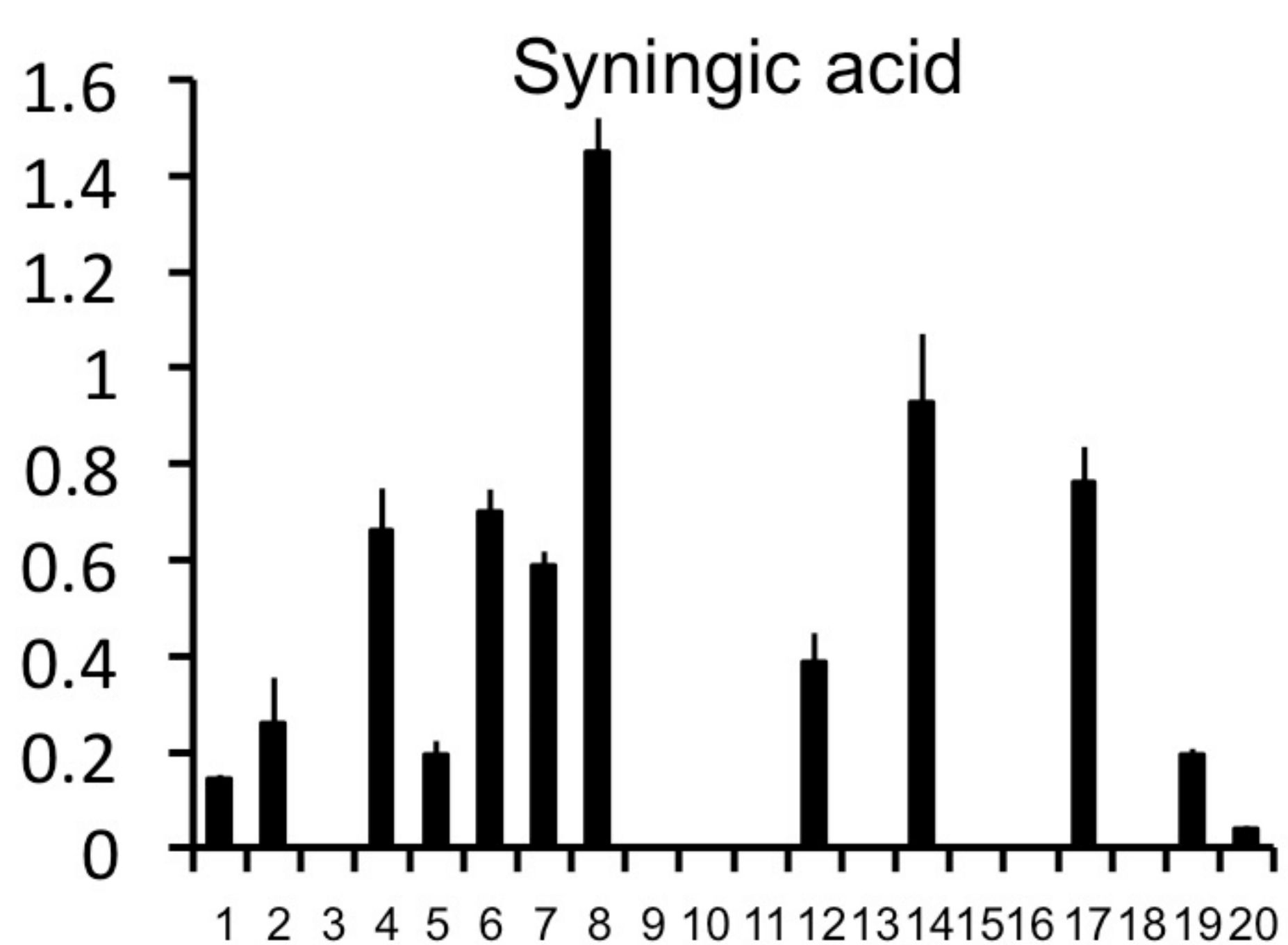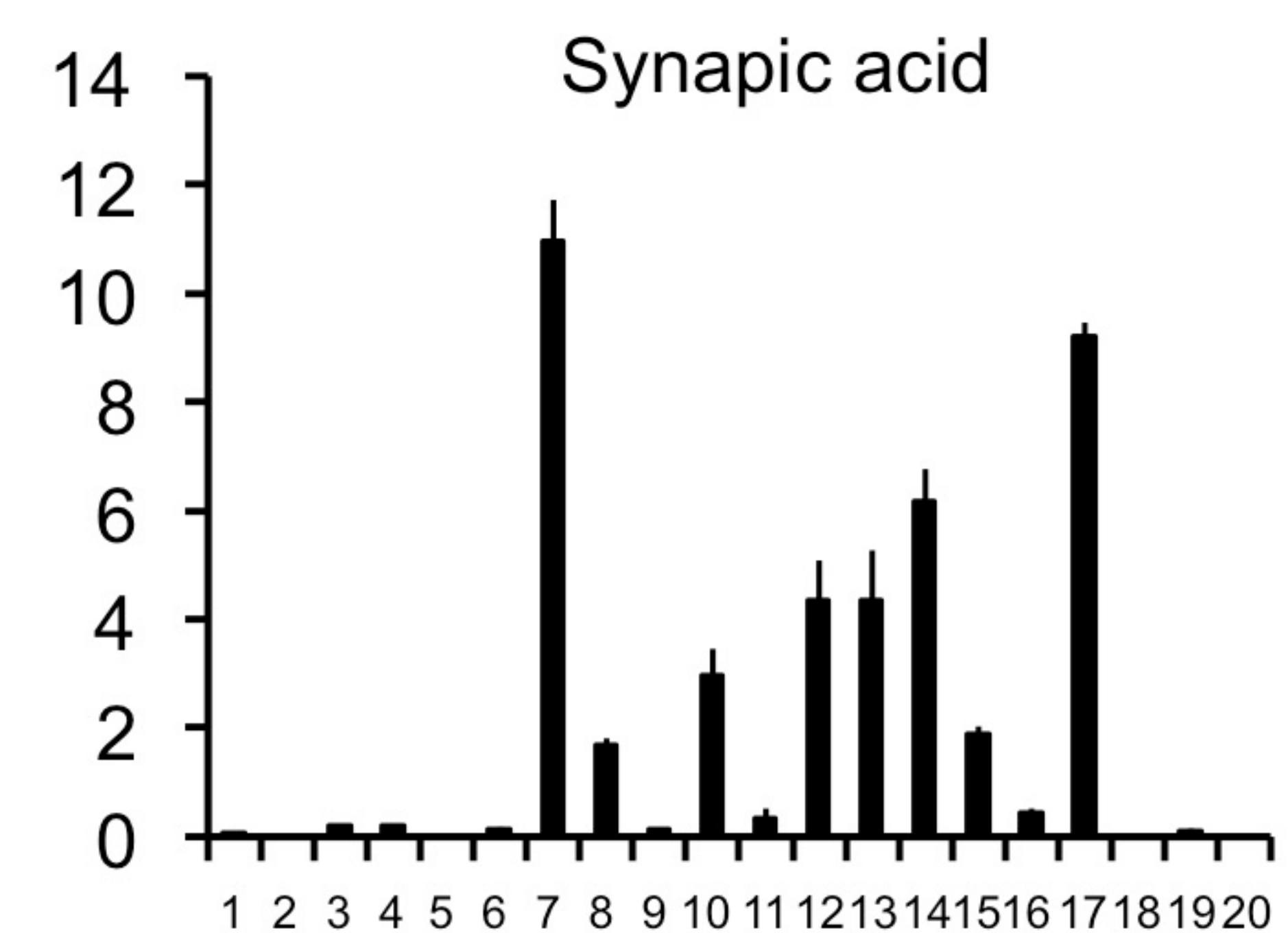

Species number

Supplement: Additional file 8: — Individual metabolite quantities determined by HPLC-UV analysis for 20 BIA-accumulating plant species. Plant species are designated by number, as defined in Table 1. Means ± SD were calculated using 4 replicates per species. (PDF 848 kb) [file 12870_2015_594_MOESM8_ESM.pdf]

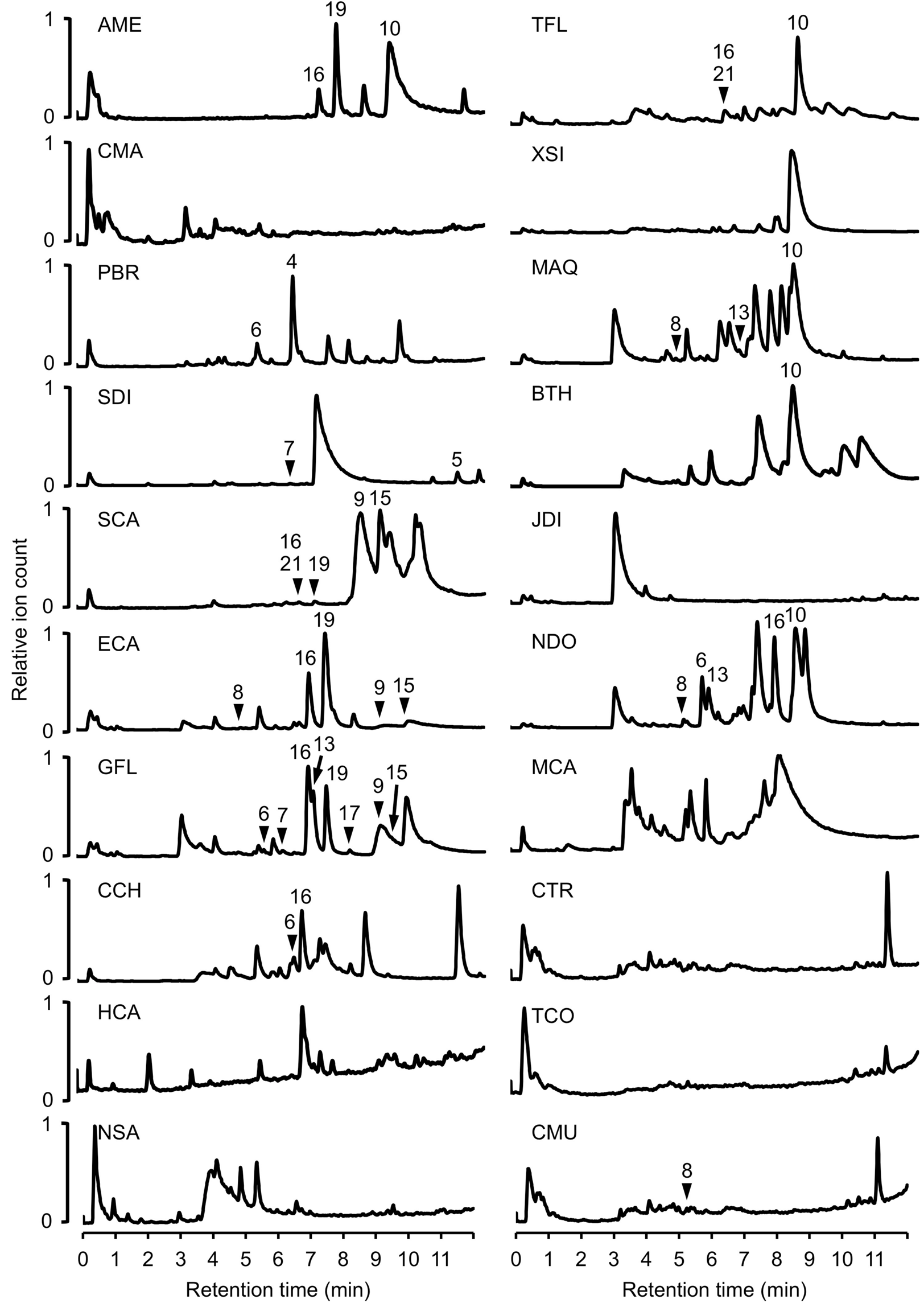

Supplement: Additional file 13: — Triple quadrupole LC-MS/MS chromatographs representing 20 BIA-accumulating plant species. Peak annotation was performed manually based on comparison with retention times (Rt) and collision-induced dissociation (CID) spectra (Additional file 14) of authentic standards. Identified peaks are numbered in correspondence with those listed in Additional file 14. Species abbreviations are defined in Table 1. (PDF 1561 kb) [file 12870_2015_594_MOESM13_ESM.pdf]
