# Supplementary material for: Metabolome analysis of 20 taxonomically related benzylisoquinoline alkaloid-producing plants
Source: BMC Plant Biol. 2015 Sep 15;15:220. doi: 10.1186/s12870-015-0594-2 (PMC4570626; doi:10.1186/s12870-015-0594-2)
Supplement: Additional file 2: — Selected examples of BIA structural subgroups derived from the basic benzylisoquinoline subunit. (PDF 1198 kb) [file 12870_2015_594_MOESM2_ESM.pdf]

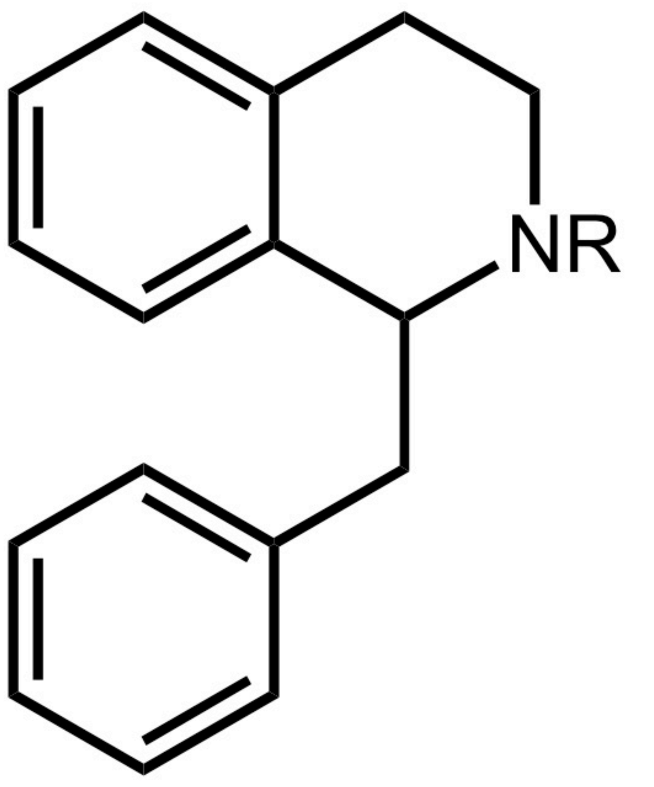

1-Benzylisoquinoline

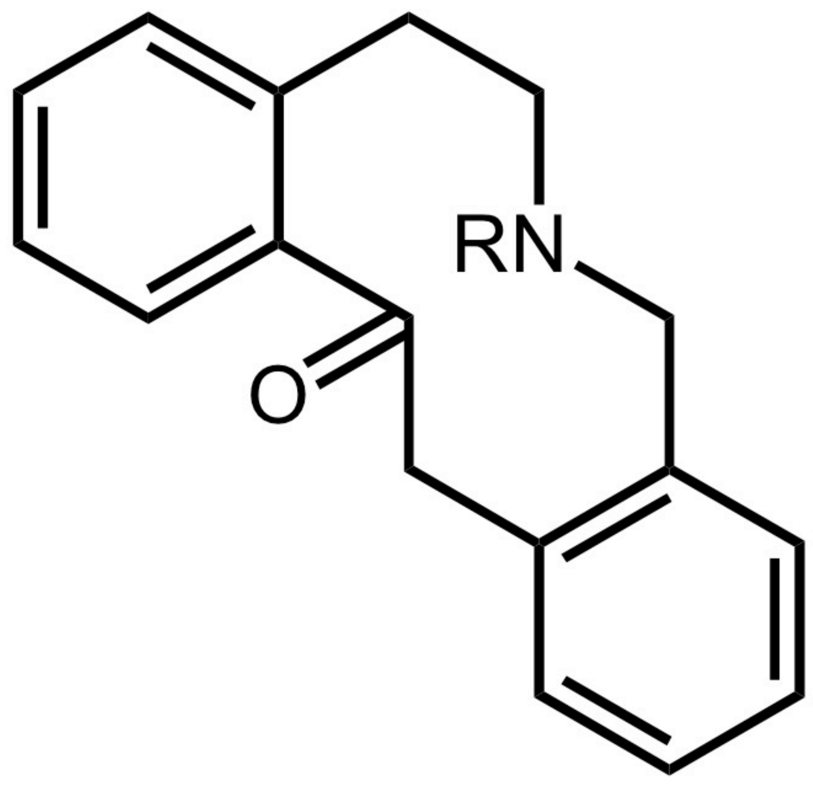

Protopine

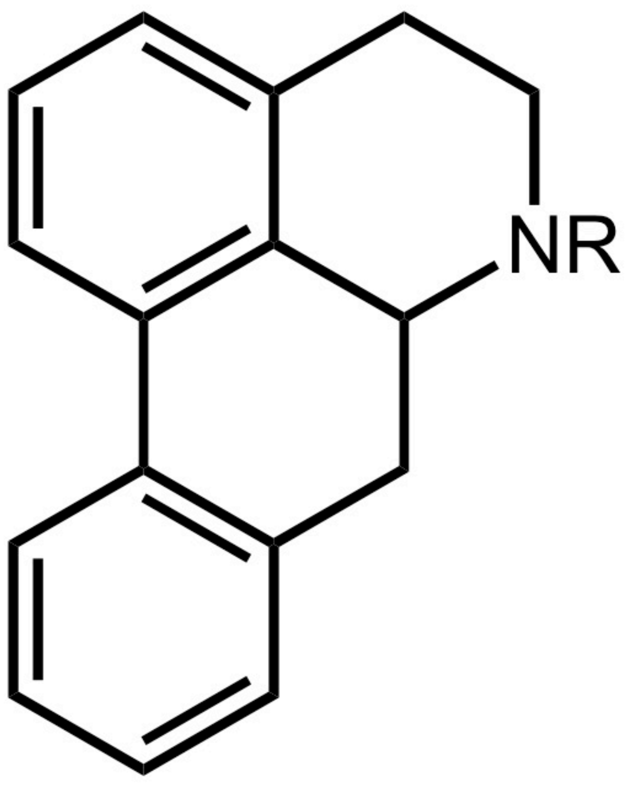

Aporphine

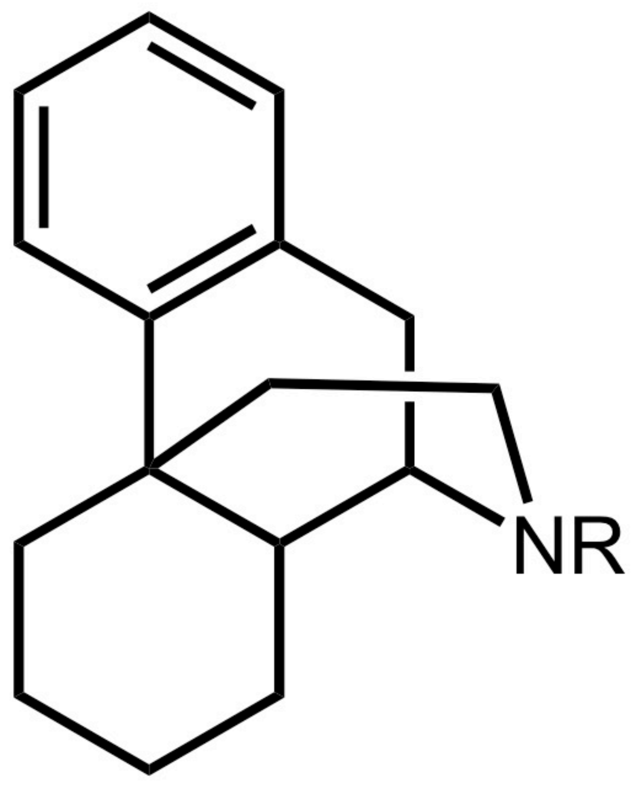

Promorphinan

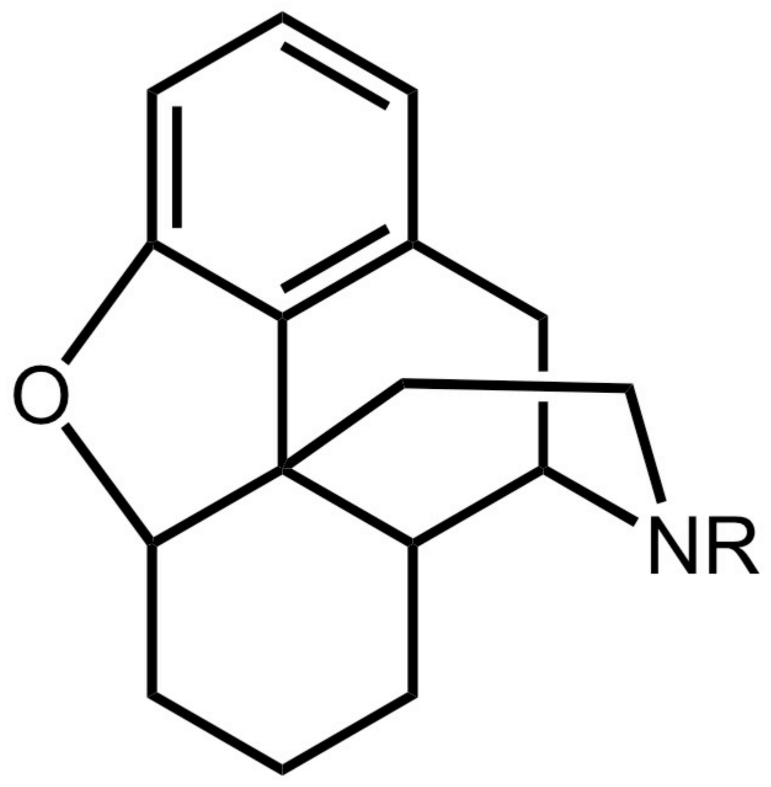

Morphinan

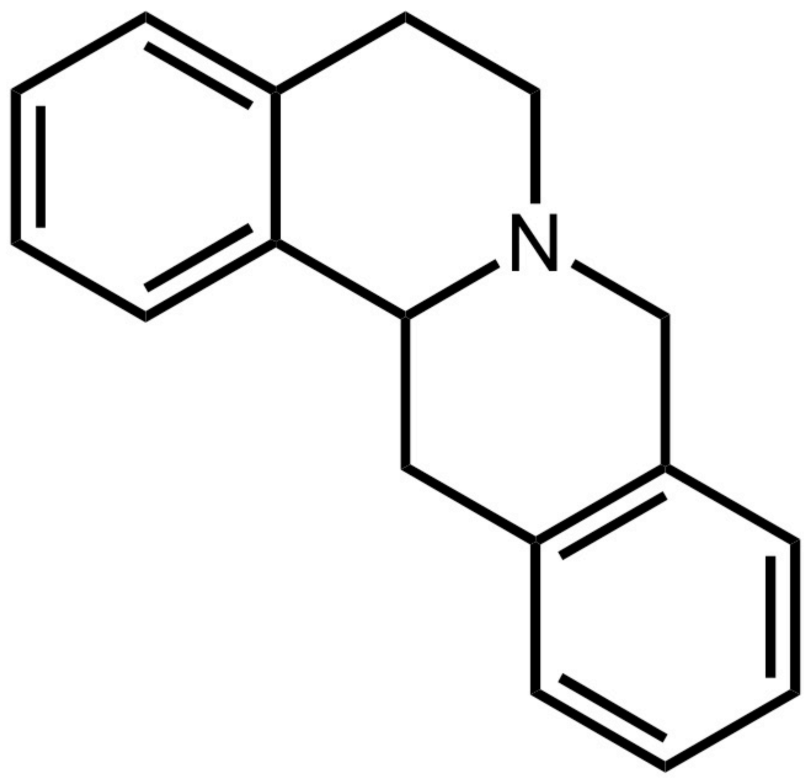

Protoberberine

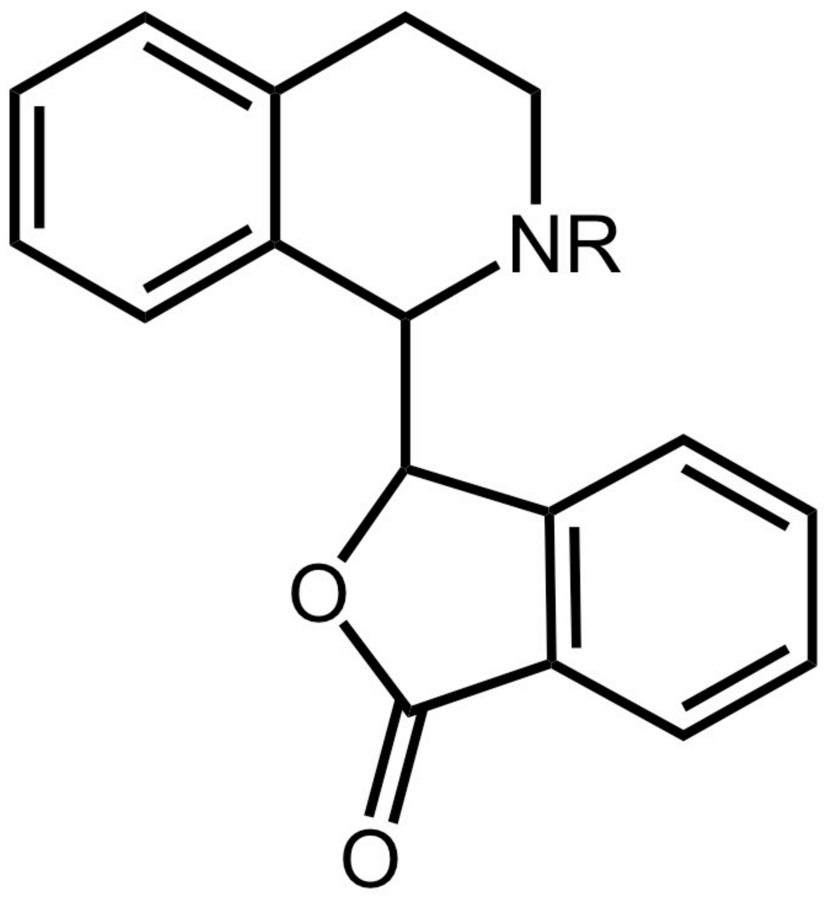

Phthalideisoquinoline

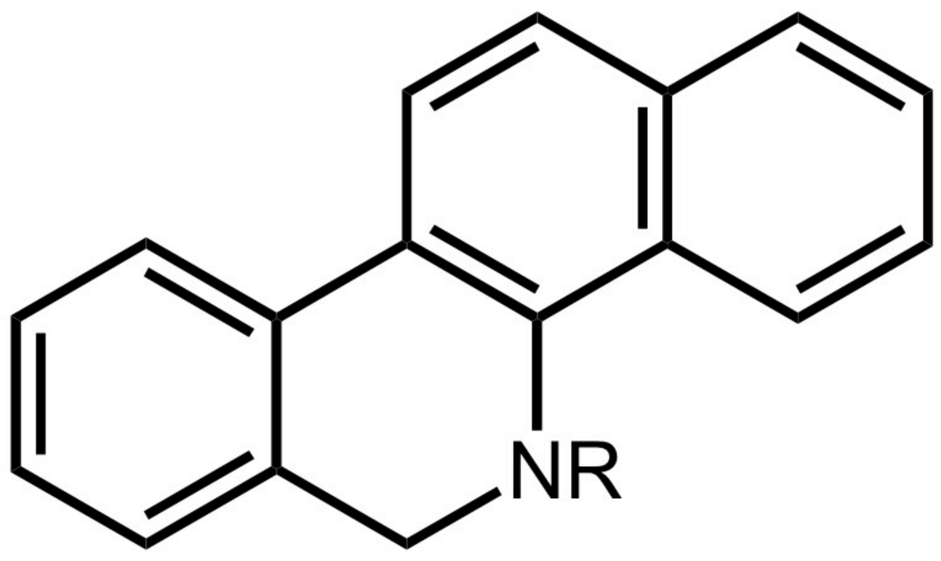

Benzo[*c*]phenanthridine

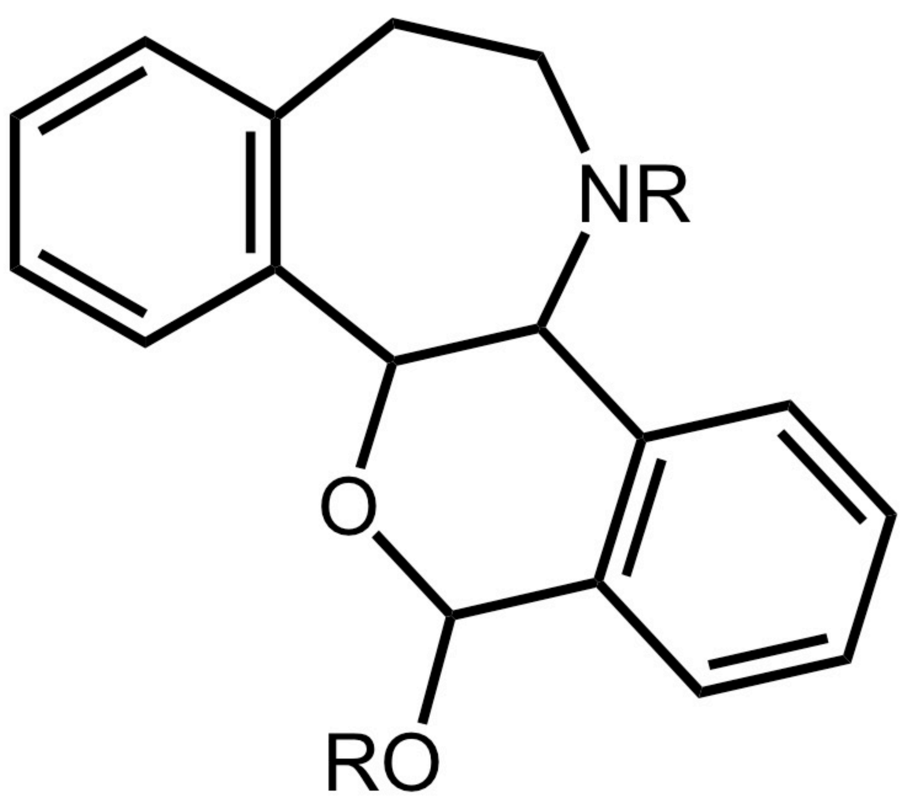

Papaverubine

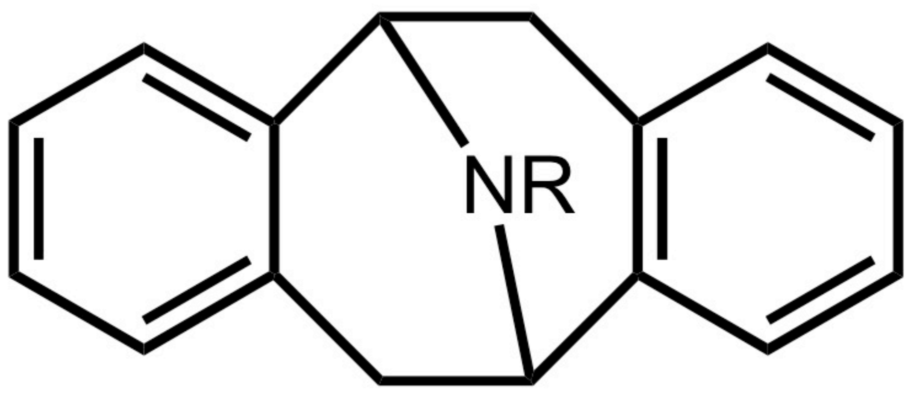

Pavine

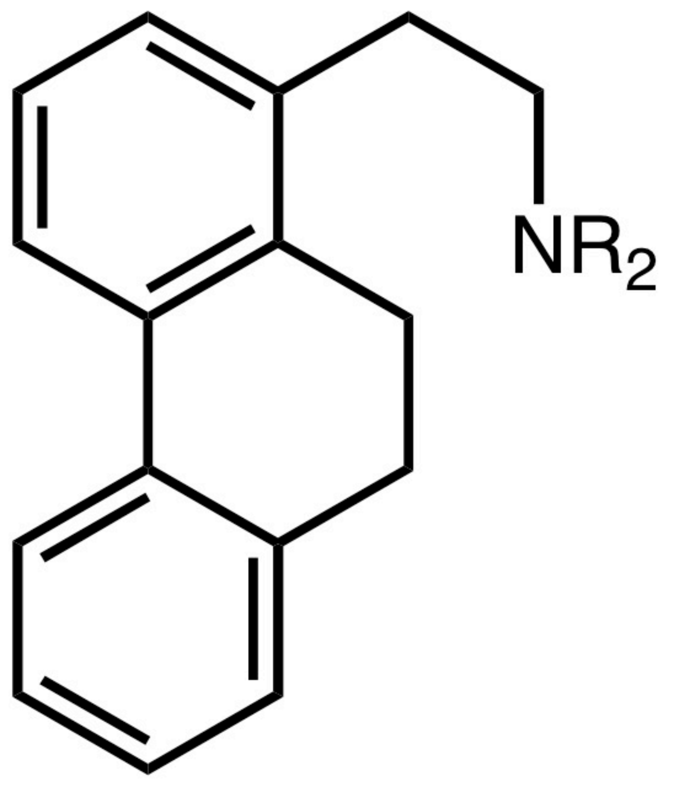

Secoisoquinoline

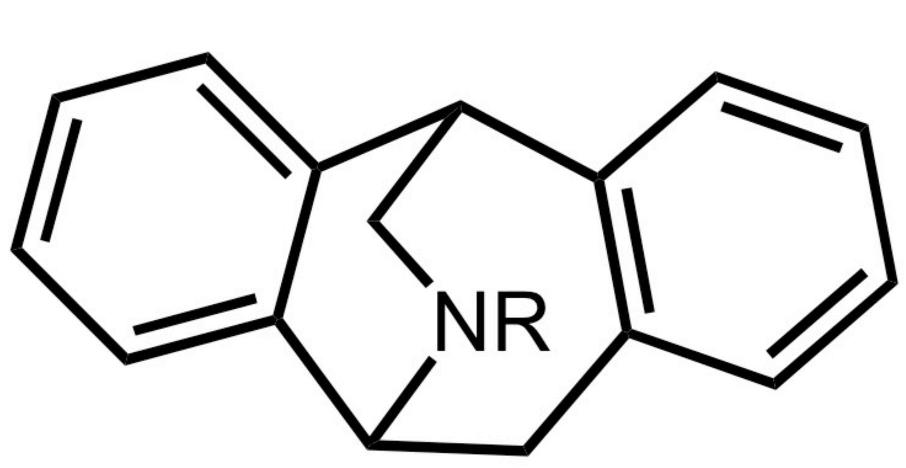

Isopavine
