# Supplementary material for: Metabolome analysis of 20 taxonomically related benzylisoquinoline alkaloid-producing plants
Source: BMC Plant Biol. 2015 Sep 15;15:220. doi: 10.1186/s12870-015-0594-2 (PMC4570626; doi:10.1186/s12870-015-0594-2)
Supplement: Additional file 12: — List of benzylisoquinoline alkaloids (BIAs), associated elemental compositions (i.e. empirical formula of singly charged ion) and theoretical ionic masses (either [M + H] + or [M] + ). In cases of structural isomerism wherein multiple BIAs possess the same composition and theoretical m/z, compounds are organized into ‘mass groups’. Only BIAs with m/z ranging from 270 to 430 are represented. (PDF 84 kb) [file 12870_2015_594_MOESM12_ESM.pdf]

m/z ([M+H]+ or [M]+	Composition	Compounds
272.12812	$C_{16}H_{18}NO_3$	Higenamine / Demethylcoclaurine / Norcoclaurine
286.14377	$C_{17}H_{20}NO_3$	Coclaurine / Morphine
292.09682	$C_{18}H_{14}N_3O_4$	*N,O*-Dimethylliriodendronine
296.12812	$C_{18}H_{18}NO_3$	Fugapavine / Mecambrine / Roemerine *N*-oxide
298.14377	$C_{18}H_{20}NO_3$	Codeinone / Isothebaidine / Oripavine
300.15942	$C_{18}H_{22}NO_3$	Codeine / *N*-Methylcoclaurine / Neopine
312.15942	$C_{19}H_{22}NO_3$	Isothebaine / Orientinine / Pronuciferine / Thebaine
314.13868	$C_{18}H_{20}NO_4$	Breogindine / Claviculine / Culacorine / Norcularidine / Norcularidine
314.17507	$C_{19}H_{24}NO_3$	Armepavine
316.15433	$C_{18}H_{22}NO_4$	Codeine *N*-oxide / 14β-Hydroxycodeine / Nororientaline / Norreticuline /
320.09173	$C_{19}H_{14}NO_4$	Coptisine / Pseudocoptisine
322.10738	$C_{19}H_{16}NO_4$	Berberrubine / Dihydrocoptisine / Dehydrocheilanthifoline / Deoxythalidastine / Escholamine / Thalifaurine / Thalifendine
324.12303	$C_{19}H_{18}NO_4$	Eschscholtzine / Stylopine
326.13868	$C_{19}H_{20}NO_4$	Amurine / Amurensine / Caryachine / Chelianthifoline / Dehydroisoboldine / *O,O*-Dimethyllongifolonine / Domesticine / Isodomesticine / Nandinine / Nornantenine / Palaudine / Pacodine
326.17507	$C_{20}H_{24}NO_3$	*N*-Methylisothebaine */ O*-Methylorientine
328.15433	$C_{19}H_{22}NO_4$	Bisnorargemonine / Bracteoline / Coreximine / Corytuberine / Dehydroreticuline / Discretamine / 16-Hydroxythebaine / Isoboldine / Munitagenine / Norcorydine / Orientalinone / Pallidine / Salutaridine / Scoulerine / Stepholidine / Thalidicine / Thalidine / Thebaine *N*-oxide
328.19072	$C_{20}H_{26}NO_3$	*N*-Methylarmepavine
330.16998	$C_{19}H_{24}NO_4$	Dihydroorientalinone / Ocobotrine / Orientaline / Reticuline / Salutaridinol
332.09173	$C_{20}H_{14}NO_4$	Sanguinarine
334.10738	$C_{20}H_{16}NO_4$	Dihydrosanguinarine / Norchelerythrine

336.12303	$C_{20}H_{18}NO_4$	Berberine / Berbericine / Epiberberine / Pseudoberberine
338.10230	$C_{19}H_{16}NO_5$	Arosinine / Thalidastine
338.13868	$C_{20}H_{20}NO_4$	Californidine / Columbamine / Dehydrodiscretine / Jatrorrhizine / *N*-Methylcalifornine / *N*-Methylescholzine / N-Methylstylopine / Palmatrubine / Pseudocolumbamine / Pseudojatrorrhizine
340.11795	$C_{19}H_{18}NO_5$	Eschscholtzine *N*-oxide / 13-Hydroxystylopine / Norchelidonine
340.15433	$C_{20}H_{22}NO_4$	Amurensinine / Canadine / Domestine / Eschscholtzidine / Escholdine / *N*-Methylpalaudium / Papaverine / Reframine / Sinactine
342.13360	$C_{19}H_{20}NO_5$	Bulbocapnine *N*-oxide / Hydroxybulbocapnine
342.16998	$C_{20}H_{24}NO_4$	Corydine / Corypalmine / Cyclanoline / Fuzitine / Isocorydine / Isocorypalmine / Isonorargemonine / Magnoflorine / *N*-Methyllaurotetanine / *O*-Methylisoboldine / *O*-Methylflavinanthine / Norargemonine / Platycerine / Tetrahydrocolumbamine / Tetrahydrojatrorrhizine / Tetrahydropapaverine / Thalisopavine / Trilobinine / Zizyphusine
344.14925	$C_{19}H_{22}NO_5$	Danguyelline / Salutaridine *N*-oxide
344.18563	$C_{20}H_{26}NO_4$	Codamine / Laudanidine / Laudanine / Norlaudanosine / Tembetarine / Thalifendlerine
348.08665	$C_{20}H_{14}NO_5$	Oxysanguinarine
348.12303	$C_{21}H_{18}NO_4$	Chelerythrine / Nitidine
350.13868	$C_{21}H_{20}NO_4$	Dihydrochelerythrine
352.11795	$C_{20}H_{18}NO_5$	Berberastine / Glauvine / Oxyglaucine / Oxyberberine
352.15433	$C_{21}H_{22}NO_4$	Palmatine / Pseudopalmatine / Thalphenine
354.13360	$C_{20}H_{20}NO_5$	Chelidonine / Papaveraldine / Protopine / Pseudoprotopine / Thalimicrinone / Thalprzewalskiinone
354.16998	$C_{21}H_{24}NO_4$	Dehydroglaucine / *N*-Methylcanadine / *N*-Methylnantenine / Takatonine

356.14925	$C_{20}H_{22}NO_5$	Baicaline / 2-Demethylthalimonine / 9-Demethylthalimonine / Dihydroprotopine / 4-Hydroxyeschscholtzidine / Hydroxynantenine / *N*-Hydroxynorthalictuberine / *N*-Methylcassythine / *O*-Methylcassythine
356.18563	$C_{21}H_{26}NO_4$	Argemonine / Escholinine / Glaucine / *O*-Methylplatycerinium / *N*-Methyltetrahydrocolumbamine / Tetrahydropalmatine / Thalictuberine / Xanthoplanine
358.16490	$C_{20}H_{24}NO_5$	Delporphine Menispermine / *N*-Methyldanguyelline / Noroconovine / Thalbaicaline / Thalicmidine *N*-oxide
358.20128	$C_{21}H_{28}NO_4$	Laudanosine / *N*-Methyltetrahydropapaverine
362.10230	$C_{21}H_{16}NO_5$	Chelirubine
364.11795	$C_{21}H_{18}NO_5$	Dihydrochelirubine / Oxochelerythrine
368.11341	$C_{21}H_{18}NO_5$	Bicuculline / Capnoidine
368.14925	$C_{21}H_{22}NO_5$	*N*-Methylpapaveraldine
370.12851	$C_{20}H_{20}NO_6$	Isorhoeagenine / Papaverrubine A / Papaverrubine E / Rhoeagenine
370.16490	$C_{21}H_{24}NO_5$	Allocryptopine / Cryptopine / Canadaline
370.20128	$C_{22}H_{28}NO_4$	Corydaline / *N*-Methyltetrahydropalmatine
374.19620	$C_{21}H_{28}NO_5$	2’-Hydroxylaudanosine
378.09721	$C_{21}H_{16}NO_6$	Oxochelirubine
378.13360	$C_{22}H_{20}NO_5$	Chelilutine
384.14416	$C_{21}H_{22}NO_6$	Hydrastine / Isorhoeadine / Oxocryptopine / Rhoeadine
384.18055	$C_{22}H_{26}NO_5$	Dehydrothalicsimidine / Thalihazine
386.15981	$C_{21}H_{24}NO_6$	Glaucamine / *N*-Methylporphyroxine / Papaverrubine B / Papaverrubine F / Preocoteine
392.11286	$C_{22}H_{18}NO_6$	Macarpine
394.12851	$C_{22}H_{20}NO_6$	Dihydromacarpine
398.15981	$C_{22}H_{24}NO_6$	Orientalidine
400.13908	$C_{21}H_{22}NO_7$	Narcotoline
400.17546	$C_{22}H_{26}NO_6$	Glaudine / Leucoxylonine / Macrantaldehyde / Mecambridine / N-Methylpapverubine / 13-Oxomuramine / Oreophiline
402.19111	$C_{22}H_{28}NO_6$	Macrantaline
414.15473	$C_{22}H_{24}NO_7$	Noscapine
416.17038	$C_{22}H_{26}NO_7$	Narcotine hemiacetal
428.17038	$C_{23}H_{26}NO_7$	*N*-Methylnarcotine
