# Supplementary material for: Metabolome analysis of 20 taxonomically related benzylisoquinoline alkaloid-producing plants
Source: BMC Plant Biol. 2015 Sep 15;15:220. doi: 10.1186/s12870-015-0594-2 (PMC4570626; doi:10.1186/s12870-015-0594-2)
Supplement: Additional file 14: — Compound names, ionic masses, retention times (R t ), structures and collision-induced dissociation (CID) spectral data acquired for authentic standards used to identify BIAs in plant samples analyzed by quadrupole LC-MS/MS. (PDF 296 kb) [file 12870_2015_594_MOESM14_ESM.pdf]

No.	Compound	$[M+H]^+$ or $[M]^+$	RT (min)	CE (eV)	ESI[+]-CID spectrum *m/z* (Relative intensity)	Structure
1	Morphine	286	1.01	25	286 (100), 229 (11.01), 211 (11.97), 209 (8.65), 201 (27.03), 193 (6.91), 185 (13.14), 183 (11.34), 180.9 (8.42), 173 (12.69), 165 (12.95), 157 (5.02), 155 (11.35), 147 (6.32), 145 (5.78), 58 (11.42), 44 (6.78)	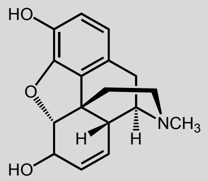
2	Oripavine	296	3.5	25	298 (2), 283 (1), 267 (1), 249 (3), 237 (1), 234 (4), 223 (1), 221 (1), 218 (8), 196 (5), 58 (100)	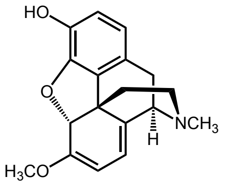
3	Codeine	300	3.52	25	300 (100), 282 (5.51), 243 (9.69), 241 (5.95), 225 (16.09), 215 (26.76), 209 (5.54), 199 (16.42), 193 (7.43), 187 (10.45), 183 (15.64), 181 (7.1), 165 (11.75), 161 (8.53), 155 (7.47), 58 (16.33), 44 (6.68)	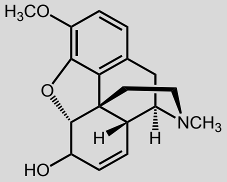
4	Thebaine	312	5.43	25	312 (2), 281 (2), 266 (4), 255 (1), 251 (11), 249 (2), 237 (1), 234 (2), 223 (2), 221 (7), 218 (4), 195 (2), 177 (1), 58 (100)	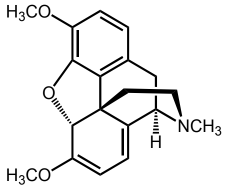
5	(*S*)-Stylopine	324	11.25	25	324 (20.4), 176 (100), 149 (39.94), 119 (6.39)	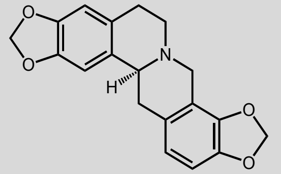
6	(*R,S*)-Boldine	328	4.98	25	297 (14.39), 282 (16.54), 267 (6.43), 266 (9.19), 265 (90.58), 237 (100), 233 (14.66), 222 (8.36), 205 (32.45), 177 (8.96), 44 (8.79)	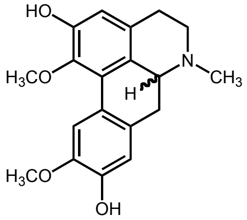
7	(*S*)-Scoulerine	328	6.21	25	328 (9.02), 178 (100), 151 (10.77)	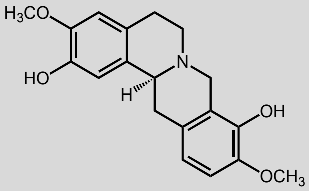
8	(*S*)-Reticuline	330	4.76	25	192 (100), 177 (8.09), 175 (19.32), 151 (5.45), 143 (25), 137 (41.49)	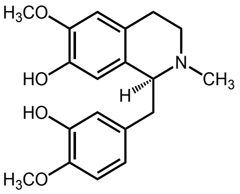

9	Sanguinarine	332	8.26	25	332 (100), 330 (6.14), 317 (15.91), 304 (22.86), 302 (7.52), 274 (14.07)	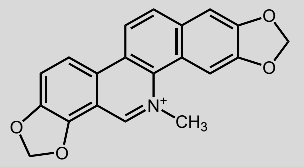
10	Berberine	336	8.02	25	336 (45.63), 321 (56.34), 320 (100), 306 (22.08), 304 (16.43), 292 (83.38), 278 (5.47), 275 (5.7)	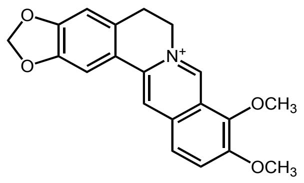
11	(*R,S*)-Canadine	340	10.28	25	340 (9.82), 176 (100), 149 (9.5)	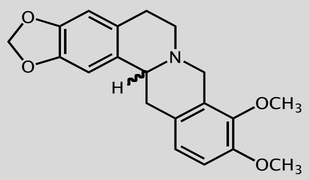
12	Papaverine	340	8.47	25	340 (70.91), 325 (7.91), 324 (74.12), 296 (11.54), 202 (100), 171 (15.27)	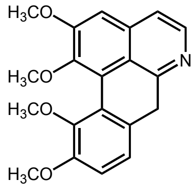
13	(*S*)-Isocorydine	342	6.97	25	311 (13.26), 296 (36.67), 281 (30.93), 280 (34.69), 279 (100), 267 (5.76), 265 (39.91), 264 (84.05), 251 (24.03), 248 (64.06), 247 (18.41), 236 (40.49), 235 (5.61), 219 (9.84), 191 (8.47)	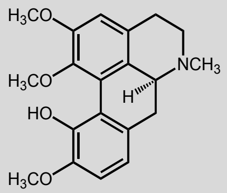
14	(*R,S*)-Tetrahydropapaverine	344	6.23	25	296 (6.8), 192 (100), 189 (33.2), 174 (16.15), 158 (11.77), 151 (51.84)	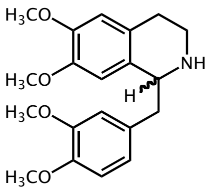
15	Chelerythrine	348	8.24	25	348 (45.51), 333 (37.27), 332 (100), 318 (31.94), 316 (8.2), 315 (8.48), 304 (56), 290 (8.73)	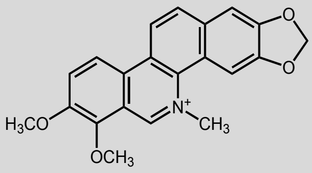
16	Protopine	354	6.81	25	354 (59.25), 336 (9.18), 271 (16.84), 265 (6.09), 247 (9.8), 206 (17.05), 189 (79.71), 188 (100), 177 (6.22), 175 (5.93), 165 (14.85), 149 (46.29), 135 (6.12)	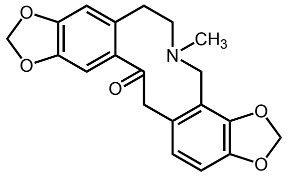

17	(*S*)-Glaucine	356	7.64	25	325 (8.04), 310 (37.78), 295 (30.25), 294 (100), 279 (27.57)	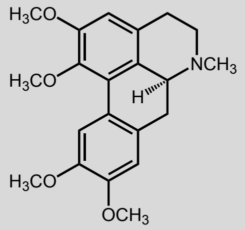
18	(*R*,*S*)-Tetrahydropalmatine	356	9.58	25	356 (10.45), 192 (100), 165 (22.38), 150 (5.5)	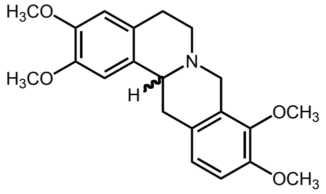
19	Allocryptopine	370	6.99	25	370 (36.41), 352 (18.72), 337 (5.03), 336 (7.29), 321 (5.84), 306 (7.59), 290 (31.63), 206 (27.8), 191 (6.21), 190 (8.31), 189 (34.38), 188 (100), 181 (18.47), 166 (6.37), 165 (13.62), 151 (9.34), 149 (9.71)	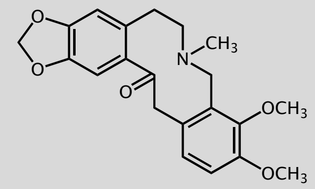
20	(*S*)-Canadaline	370	6.85	25	290 (12.64), 190 (100)	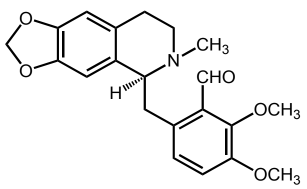
21	Cryptopine	370	6.65	25	370 (68.18), 352 (10.46), 339 (5.46), 321 (9.4), 311 (6.87), 291 (15.92), 290 (5.28), 283 (5.57), 263 (10.91), 222 (16.66), 206 (8.09), 205 (69.54), 204 (100), 194 (8.21), 193 (11.48), 190 (32.49), 175 (10.43), 165 (89.48), 151 (5.35), 150 (12.28), 149 (23.01), 135 (5.56)	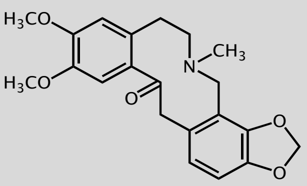
22	Hydrastine	384	7.68	25	369 (7.93), 366 (10.08), 354 (17.54), 351 (65.15), 336 (100), 333 (5.55), 308 (6)	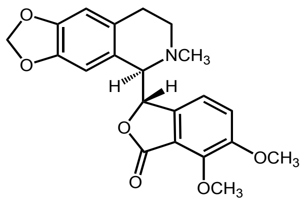
23	Noscapine	414	10.67	25	414 (5), 365 (18.39), 323 (5.23), 220 (100), 206 (5), 179 (6.43)	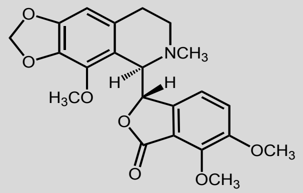
