# Supplementary material for: Metabolome analysis of 20 taxonomically related benzylisoquinoline alkaloid-producing plants
Source: BMC Plant Biol. 2015 Sep 15;15:220. doi: 10.1186/s12870-015-0594-2 (PMC4570626; doi:10.1186/s12870-015-0594-2)
Supplement: Additional file 15: — Selected examples of phthalideisoquinoline alkaloids. (R,S)-Canadaline, a presumed precursor to certain phthalideisoquinoline alkaloids, is also shown. (PDF 1419 kb) [file 12870_2015_594_MOESM15_ESM.pdf]

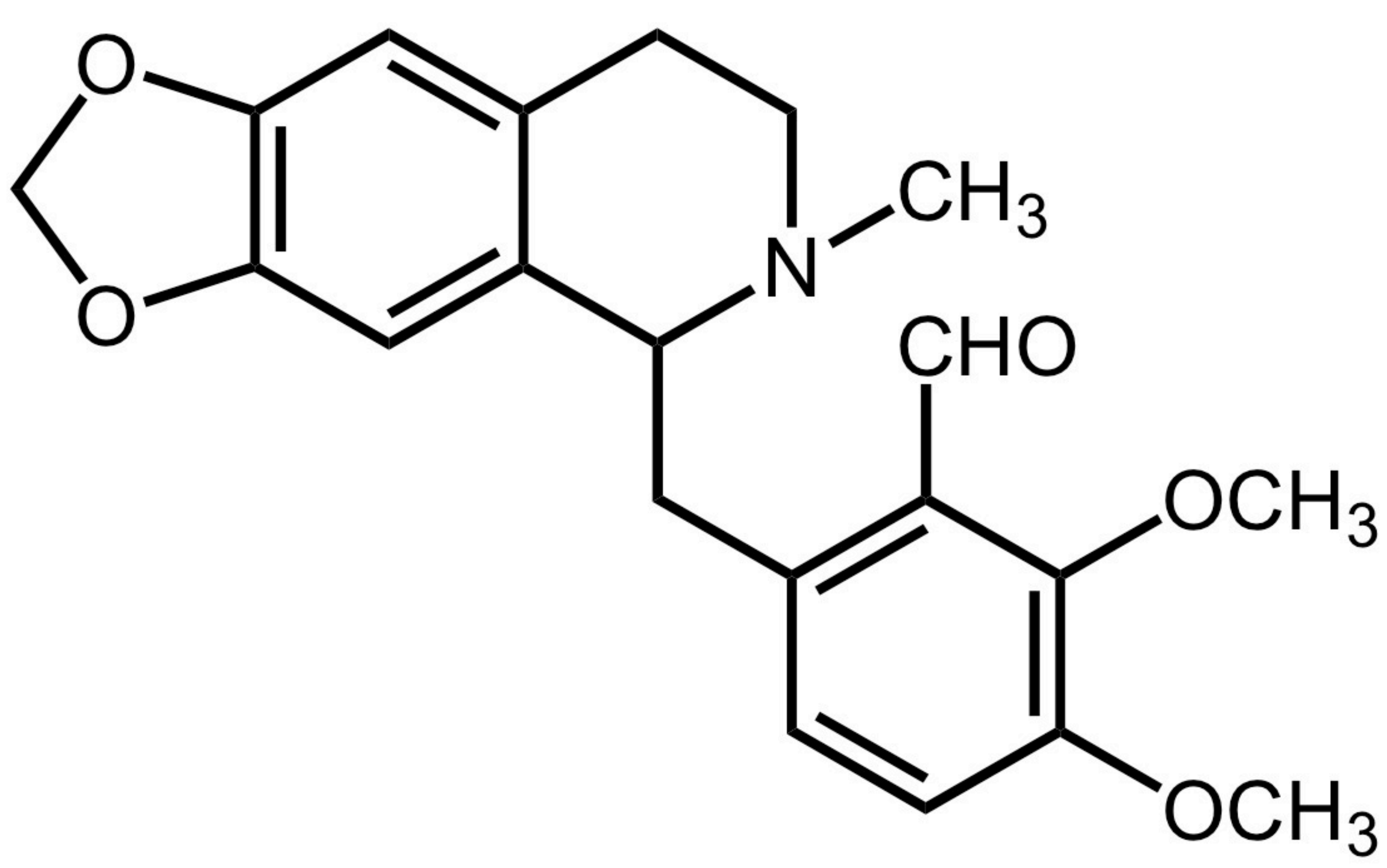

(*R,S*)-Canadaline [*m/z* 370]

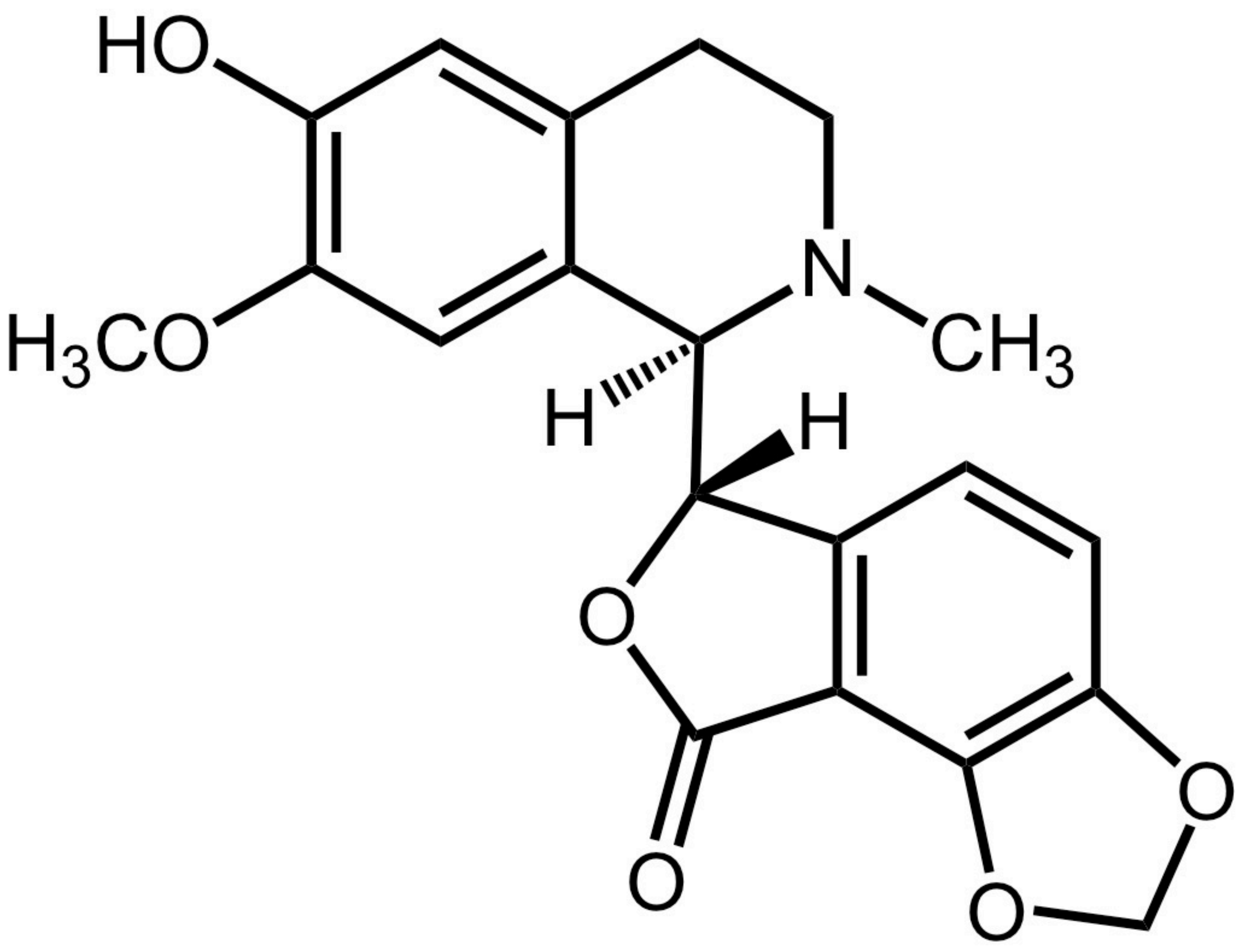

(-)-Corledine [*m/z* 370]

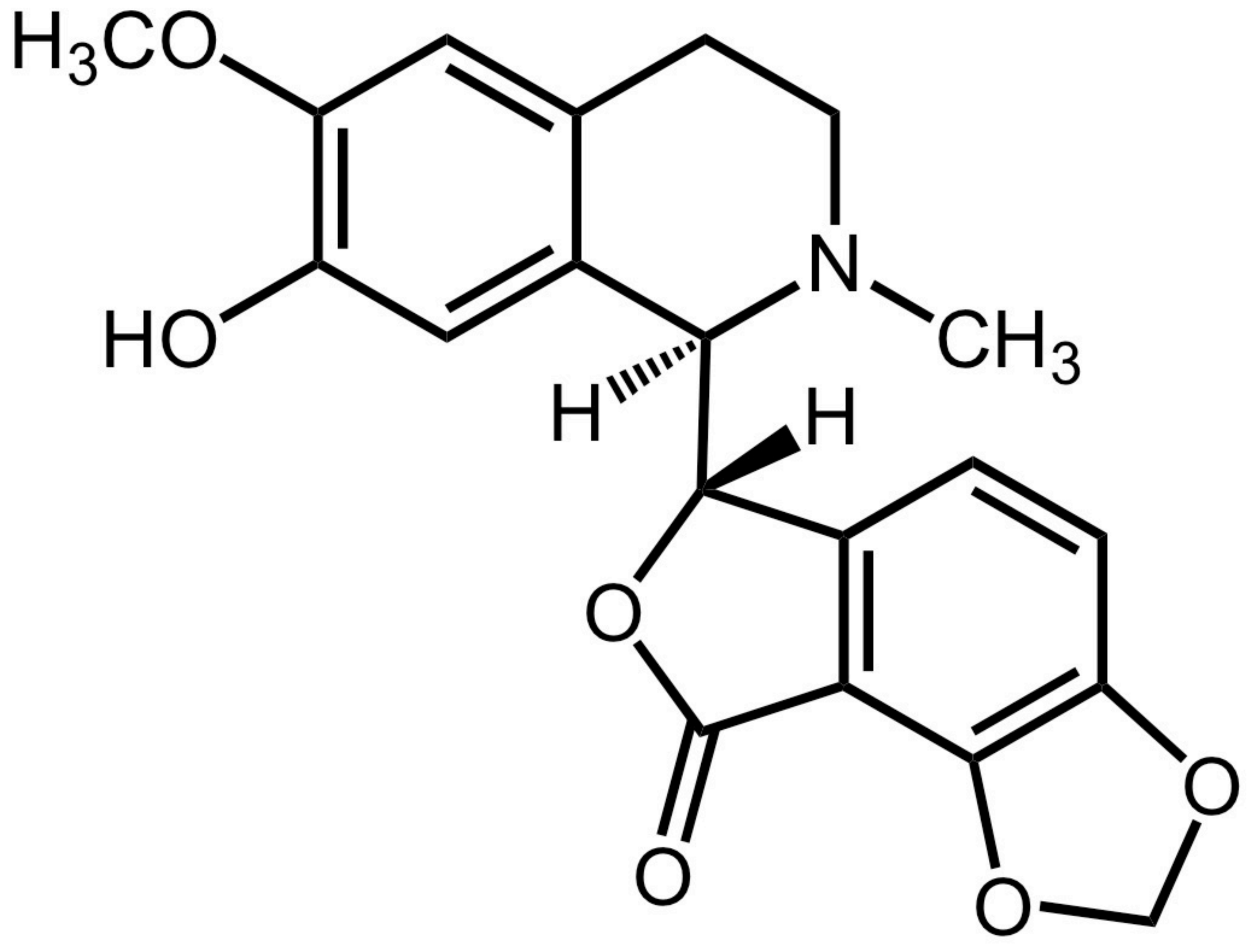

(-)-Severtzine [*m/z* 370]

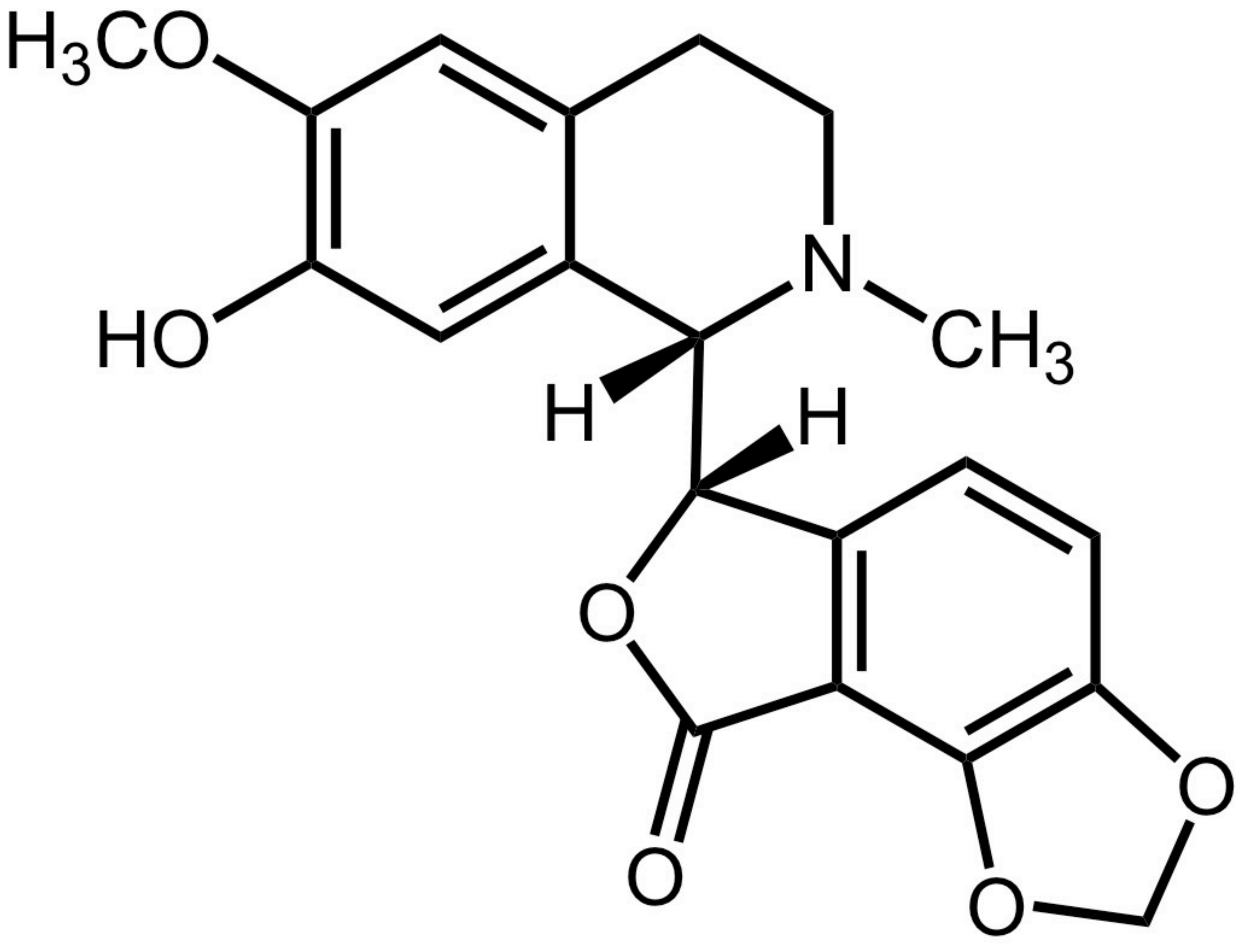

(+)-Corlumidine [*m/z* 370]

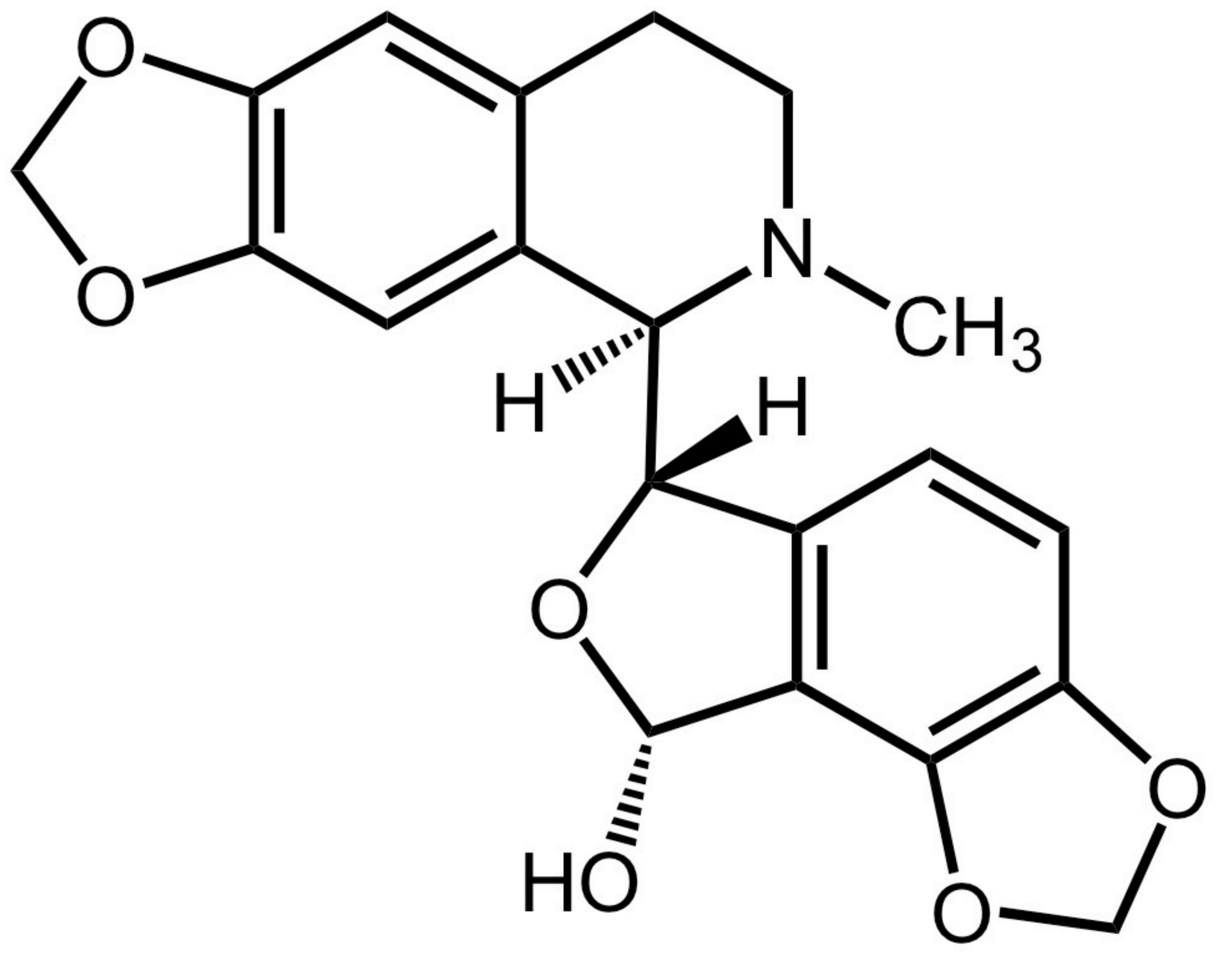

(+)-Egenine [*m/z* 370]

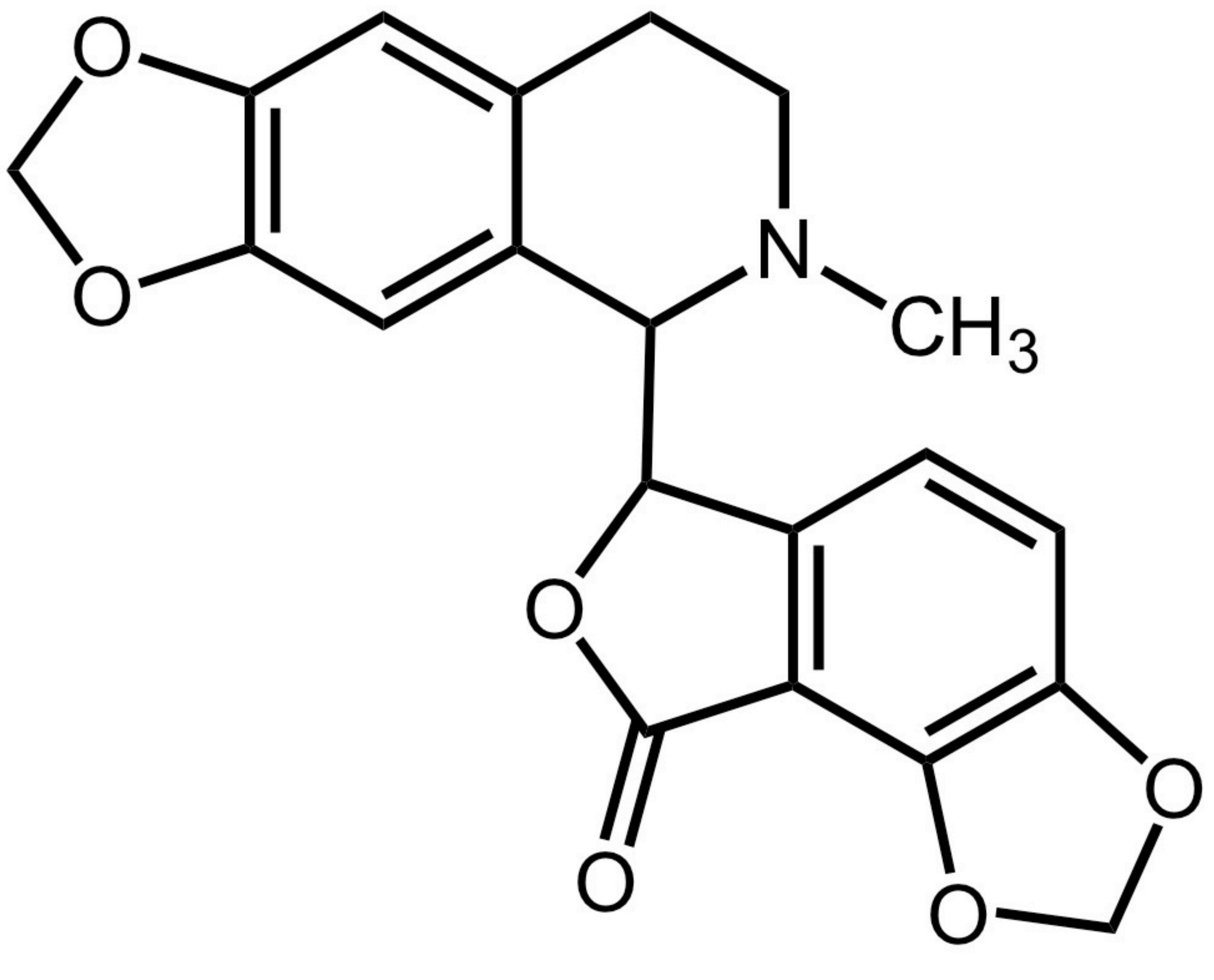

(+/-)-Bicuculline [*m/z* 368]

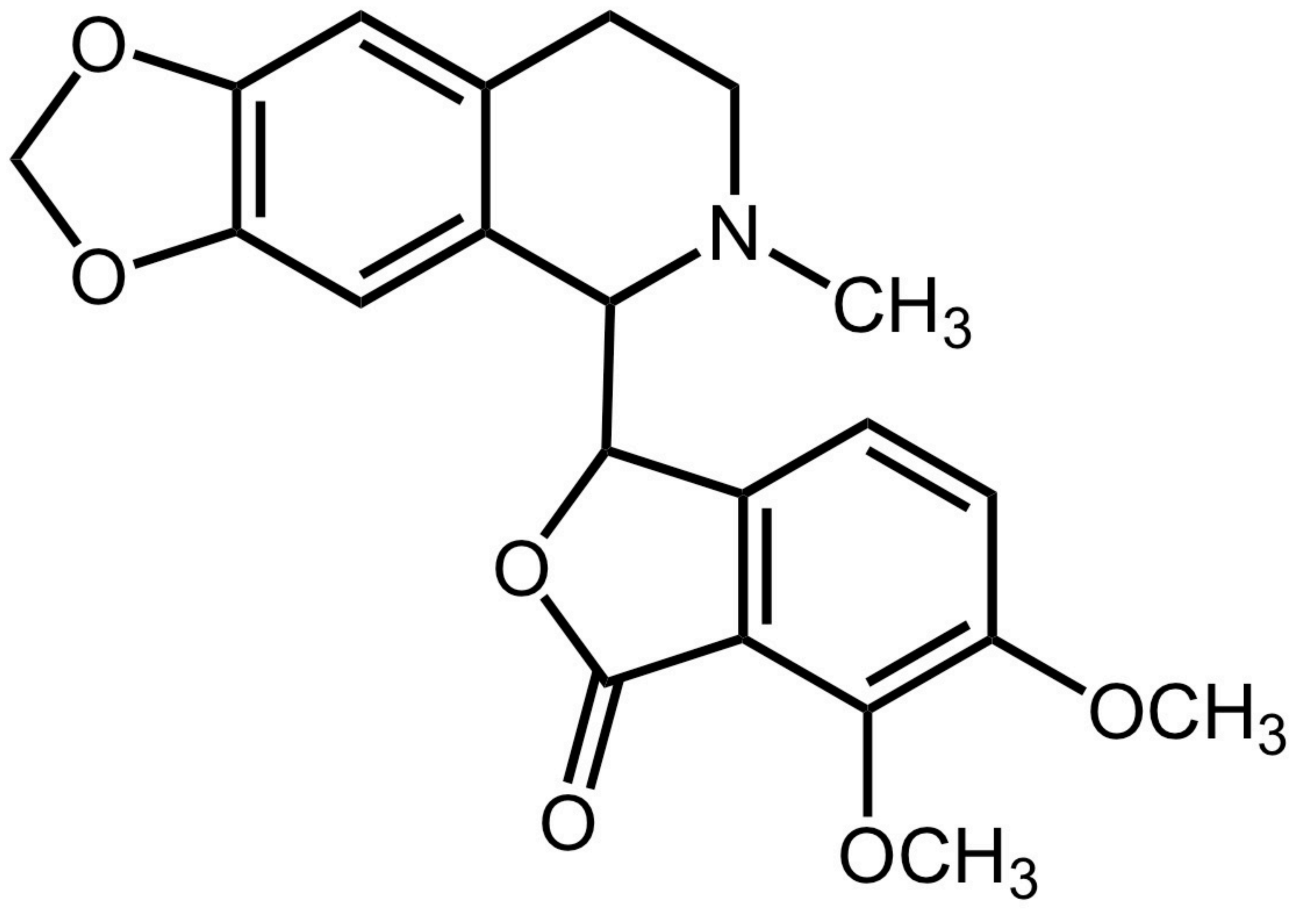

(+/-)-Hydrastine [*m/z* 384]

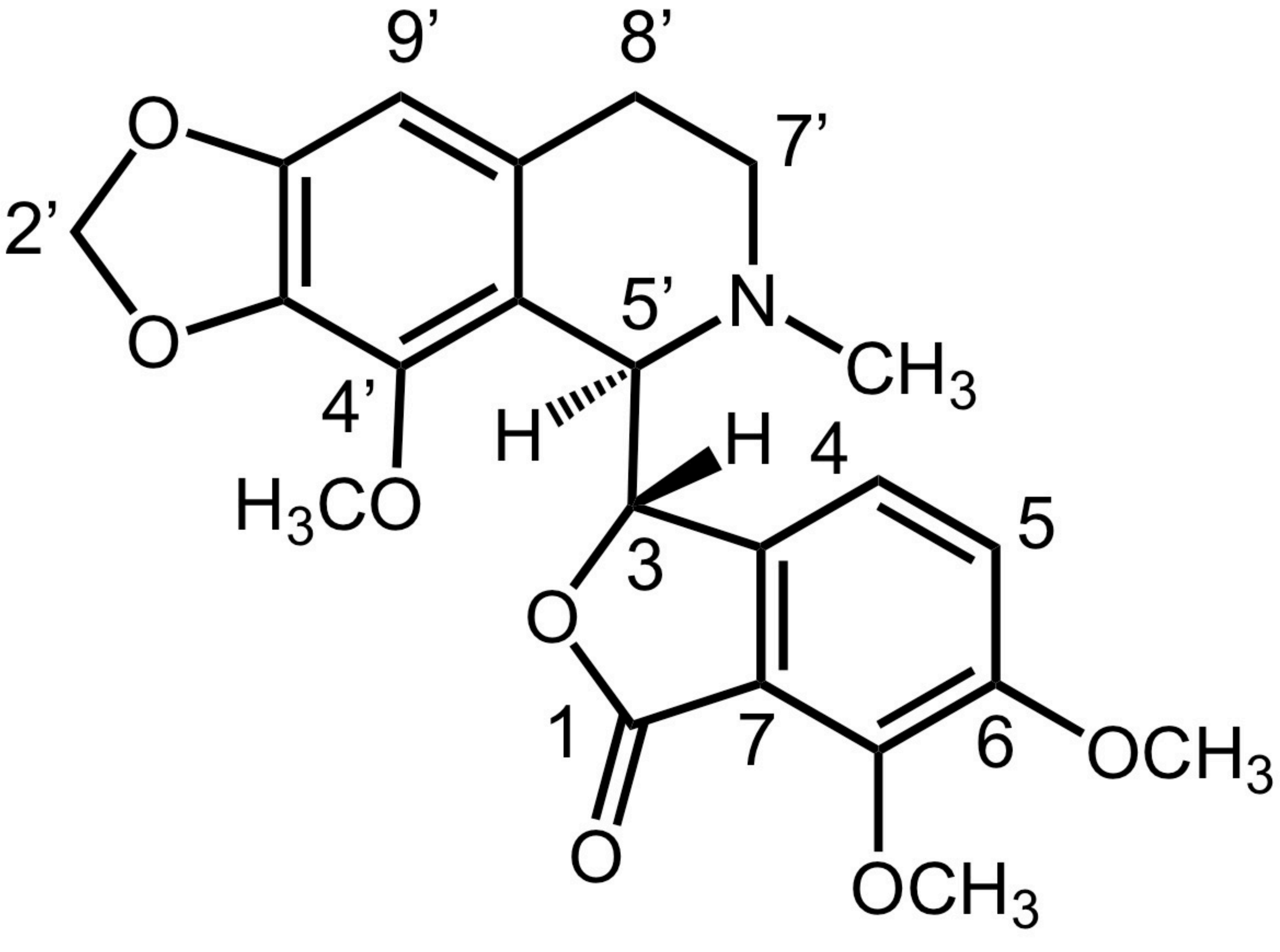

Noscapine [*m/z* 414]
